# An Ultra-Compact and Low-Cost LAMP-Based Virus Detection Device

**DOI:** 10.3390/s24154912

**Published:** 2024-07-29

**Authors:** Dong Guo, Zhengrong Ling, Yifeng Tang, Gen Li, Tieshan Zhang, Haoxiang Zhao, Hao Ren, Yajing Shen, Xiong Yang

**Affiliations:** 1 Shenzhen Research Institute, City University of Hong Kong, Shenzhen 518000, China; 2 Department of Biomedical Engineering, City University of Hong Kong, Hong Kong 999077, China; 3 Department of Electronic and Computer Engineering, Hong Kong University of Science and Technology, Hong Kong 999077, China

**Keywords:** portable molecular detection, LAMP, color recognition algorithm, viral detection

## Abstract

Timely and accurate detection of viruses is crucial for infection diagnosis and treatment. However, it remains a challenge to develop a portable device that meets the requirement of being portable, powerless, user-friendly, reusable, and low-cost. This work reports a compact ∅30 × 48 mm portable powerless isothermal amplification detection device (material cost ∼$1 USD) relying on LAMP (Loop-Mediated Isothermal Amplification). We have proposed chromatographic-strip-based microporous permeation technology which can precisely control the water flow rate to regulate the exothermic reaction. This powerless heating combined with phase-change materials can maintain a constant temperature between 50 and 70 °C for a duration of up to 49.8 min. Compared with the conventional methods, it avoids the use of an additional insulation layer for heat preservation, greatly reducing the size and cost. We have also deployed a color card and a corresponding algorithm to facilitate color recognition, data analysis, and storage using a mobile phone. The experimental results demonstrate that our device exhibits the same limit of detection (LOD) as the ProFlex PCR for SARS-CoV-2 pseudovirus samples, with that for both being $10^{3}$ copies/μL, verifying its effectiveness and reliability. This work offers a timely, low-cost, and easy way for respiratory infectious disease detection, which could provide support in curbing virus transmission and protecting the health of humans and animals, especially in remote mountainous areas without access to electricity or trained professionals.

## 1. Introduction

Alternating infections of different respiratory viruses (such as SARS-CoV-2, influenza, etc.) in humans and livestock have led to the rapid spread of diseases due to delayed detection of the infecting viruses [1,2,3], resulting in significant societal and economic losses [4,5,6,7]. The rapid detection of viruses is regarded as a significant method for early screening and preventing virus transmission [8,9]. Nowadays, PCR (Polymerase Chain Reaction) testing has become the gold standard for diagnosing respiratory viral infections owing its advantages of high sensitivity and specificity [10,11,12]. However, PCR’s limitations, including its large size and requirement for specialized laboratory conditions, restrict its usage only to hospitals and laboratories. In recent years, portable PCR devices for virus detection have attracted great attention [13]. Nevertheless, the PCR amplification process requires multiple temperature cycles to achieve sample amplification. This necessitates the use of temperature control chips, temperature sensors, and control circuits as key components [14]. While these devices may be smaller than traditional desktop PCR machines, their complex operation and cost still prevent their widespread use in economically disadvantaged areas and field settings without access to power sources [13,15,16,17]. Isothermal amplification techniques, such as LAMP, only require a constant temperature for the amplification reaction [18,19,20]. This simplifies temperature control and allows for the development of more portable devices that can even operate without an external power source and control circuitry, surpassing the limitations of portable PCR devices [21,22,23]. Previous studies have explored various miniaturized power-free detection devices. For instance, Li R. et al. proposed the use of chemical exothermic reaction packs to provide the necessary heat for isothermal amplification. They mixed a Mg-Fe alloy (commonly used for food heating) directly with phase-change materials as a heating pack. By effectively integrating the heat source and the phase-change materials and employing Styrofoam to reduce the heat dissipation, they were able to maintain a constant temperature within the amplification chamber for the detection of SARS-CoV-2 amplification [24]. Song J. et al. utilized a modified stainless steel thermos cup containing a one-time exothermic reaction of a Mg-Fe alloy with water. They introduced phase-change materials between the reaction sample and the heat pack, achieving temperature control through thermal coupling. Targeting Zika Virus in oral samples, they successfully amplified and detected five plaque-forming units within 40 min [25]. Although these portable PCR devices can meet the needs for their use in environments without a power supply, they typically employ centralized heating reactions, which require additional integration of temperature control components to maintain a relatively constant-temperature environment. To prevent heat loss, the thermal insulation layer commonly consists of double-layer vacuum stainless steel insulation bottles, which unavoidably increase the size of the devices, usually approximately 90 mm in diameter [26,27,28,29,30]. Fusion design of the phase-change materials and heating materials renders the phase-change materials non-reusable, resulting in significant waste and environmental pollution. Moreover, the devices usually rely on manual water addition from external sources, which is not suitable for field operations and may pose a risk of burns to users. In short, the current techniques still face challenges in terms of the large-sized equipment, their inability to be reused multiple times, their relatively high cost, their lack of portability, and the requirement for additional water sources. A comparative analysis of different portable thermal cycling amplification devices from recent years is shown in Table 1.

**Figure 1:**
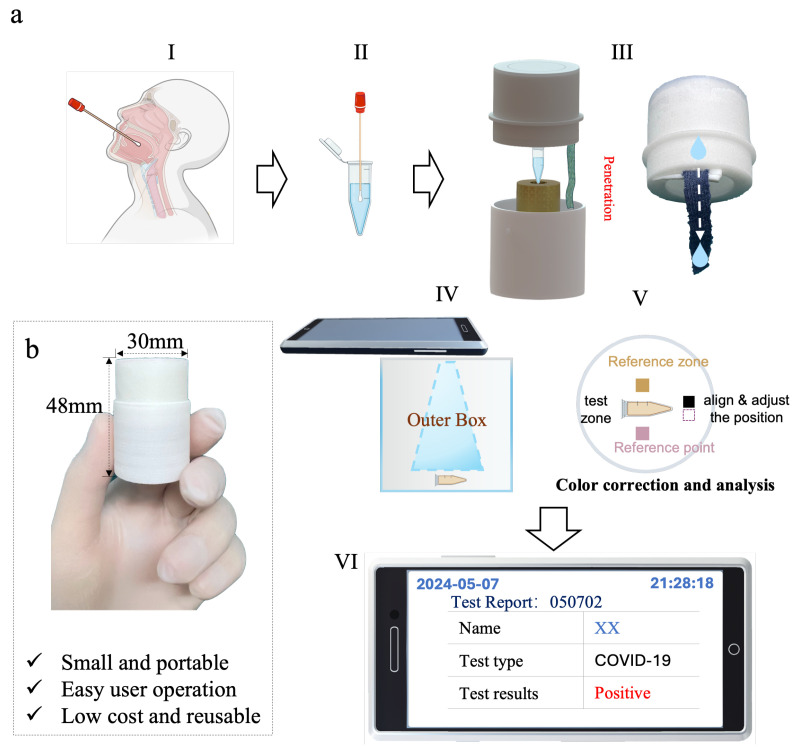
Overall concept diagram and physical diagram. (**a**) (I) Sample collection; (II) loading the sample into the reaction system (I and II created with BioRender.com); (III) covering the device to start the reaction, with a physical picture of the cover; (IV) capturing the detection result through a mobile application and uploading it to the cloud; (V) color card used to improve color recognition accuracy; (VI) finally displaying the test results. (**b**) The overall device size is 30 × 48 mm, which is easy to carry.

In this paper, we propose a method of controlling the heat in the amplification chamber by regulating the speed and duration of a controlled exothermic reaction. By eliminating the need for bulky vacuum insulation, our device can achieve a more portable size (∅48 × 30 mm) and lower material costs ($1 USD). The effectiveness and reliability of the device are verified using a mock virus experiment with four gradients, which shows the same results as a professional PCR device (ProFlex PCR 3 × 32) with LAMP. Moreover, we also developed a smartphone application to analyze and record the result, offering similar result recognition and documentation capabilities to professional equipment. The overall operating process is shown in Figure 1. This ultra-low-cost and highly portable detection device is reusable and provides an effective solution for personal testing at home, in impoverished areas, and in a field environment [36,37].

## 2. Working Principle and Device Design

### 2.1. Working Principle and Process

Our device does not employ an additional insulation layer to maintain a constant temperature. Instead, it utilizes control over the rate of the exothermic reaction to provide continuous heat and maintain a constant temperature, which allows for a smaller device size. The detection of viruses and other pathogens is achieved through the utilization of the LAMP technique. The required heat is generated by the exothermic reaction between water and CaO-Al, Na_2_CO_3_ [17,38]. By controlling the rate of the exothermic reaction, continuous release of heat is maintained. Where the amplification chamber is constructed using copper and phase-change materials, ensuring a constant internal temperature even when the heat source temperature fluctuates within a certain range.

To perform a test, the user simply needs to add the sampled specimen to a test tube containing the reaction reagents. The test tube is then placed in our device, and on tearing open the seal of the water inlet, the upper cover is closed, initiating the reaction. After 50 min, the reaction is complete, and the test tube can be removed. The color change in the sample can be visually observed to initially determine the presence of a positive result. Additionally, a mobile application integrated into our device allows users to capture images and utilize our built-in recognition algorithm to accurately identify their test results and retain their health data. This enables monitoring of the virus infection status of the individual over a certain period of time.

### 2.2. Device Design

**Figure 2:**
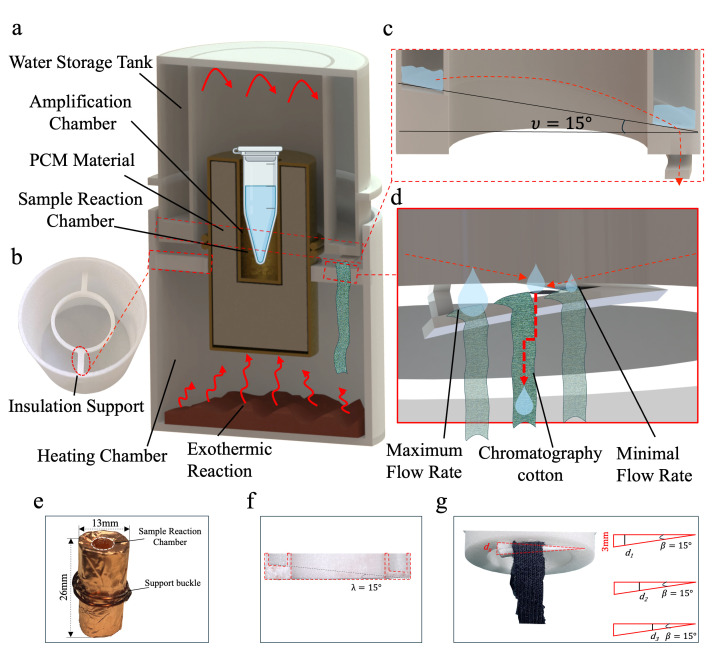
The overall structure of the device and a functional description of its key parts. (**a**) Exploded view of the portable amplification device. The device is composed of a cover (water storage tank), a heating chamber, an amplification chamber wrapped in PCM, and a heat source pack of CaO-Al and Na_2_CO_3_ reactions. (**b**) Physical picture of the heating chamber, showing its internal insulation support. (**c**) Exploded view of the interior of the cover (water storage tank); the highest and lowest points inside form a 15° slope. (**d**) Device outlet, composed of a micro-dropping hole and movable chromatographic cotton. (**e**) Physical picture of the phase-change reaction chamber; the phase-change material is wrapped around the test sample to the maximum extent. (**f**) Physical cross-sectional view of the cover (water tank). (**g**) Physical picture of the outlet; chromatographic cotton changes $d_{x}$ by moving left and right in the wedge-shaped opening with an angle of 15°, adjusting the water flow rate and achieving control of the reaction rate.

As depicted in Figure 1a, the entire device is a cylinder comprising three major components: an upper cover (water storage tank), a heating chamber, and an amplification chamber. The water storage tank is designed to be in the outer ring of the upper cover. This design prevents water from directly flowing into the amplification chamber and affecting the temperature of the sample reaction. To control the rate of the flow of water into the reaction pouch, we have devised a mechanism by which the droplets flow directly onto the chromatographic cotton. The chromatography cotton, measuring 5 × 60 mm, is cut from a fabric known as “220-mesh-precision twill Odele food hair”. Due to the water-absorbing and permeable properties of the chromatographic cotton, controlled water release is achieved [39,40,41]. The water storage tank, as depicted in Figure 2c,f, features an asymmetric funnel design with a slope gradient of 15°. This design ensures that the internal water flow is directed towards the lowest point, which is the designated outlet hole. To accommodate the requirements for different constant temperatures, we have designed a chromatographic cotton strip release clamp, as shown in Figure 2c. This clamp features a wedge-shaped hollow structure with a wedge angle of 15°, allowing for the movement of the chromatographic cotton within the structure. At different positions, as depicted in Figure 2g, the corresponding wedge-shaped space exhibits varying heights, represented as $d_{x}$, ranging from 3 mm to 0 mm. By altering the compression level of the chromatography cotton through these different heights of the wedge-shaped space, we can achieve different water flow velocities, thereby controlling the reaction rate and further controlling the temperature during the reaction process. Compared to manually adding water, this design of storing water in the upper cover allows the water flow rate to be controlled through the device’s water outlet design. Additionally, it eliminates the need to rely on external water sources, making the operation simpler and not restricted by external water availability. The heating compartment is where the heat source is generated. The heating pack (CaO-Al, Na_2_CO_3_) is placed in the bottom of the heating compartment. The internal design of the heating chamber includes insulation brackets to support the phase-change interlayer, reducing the contact between the amplification chamber and the outer shell and slowing down the dissipation of heat in the amplification chamber, as shown in Figure 2b. Simultaneously, the phase-change characteristics of the phase-change materials are utilized to maintain the temperature of the amplification chamber near its melting point. Due to the temperature range requirement for LAMP, which is typically between 50 and 70 °C, we have incorporated a PCM (phase-change material) layer around the amplification chamber. We have selected a PCM with a 64 °C melting point (PCM-A-64, Guangzhou Zhongjia New Material Technology Co., Ltd., Guangzhou, China). The phase-change material completely wraps around the reaction tube and is encapsulated with copper, which has a thermal conductivity of 401 W/(m·K), as shown in Figure 2e. The reaction tube is completely embedded within the copper-encased phase-change material. This design ensures efficient heat exchange between the phase-change material and the reaction tube. Additionally, the copper-encapsulated phase-change material can be reused multiple times.

### 2.3. Temperature Simulation and Control

**Figure 3:**
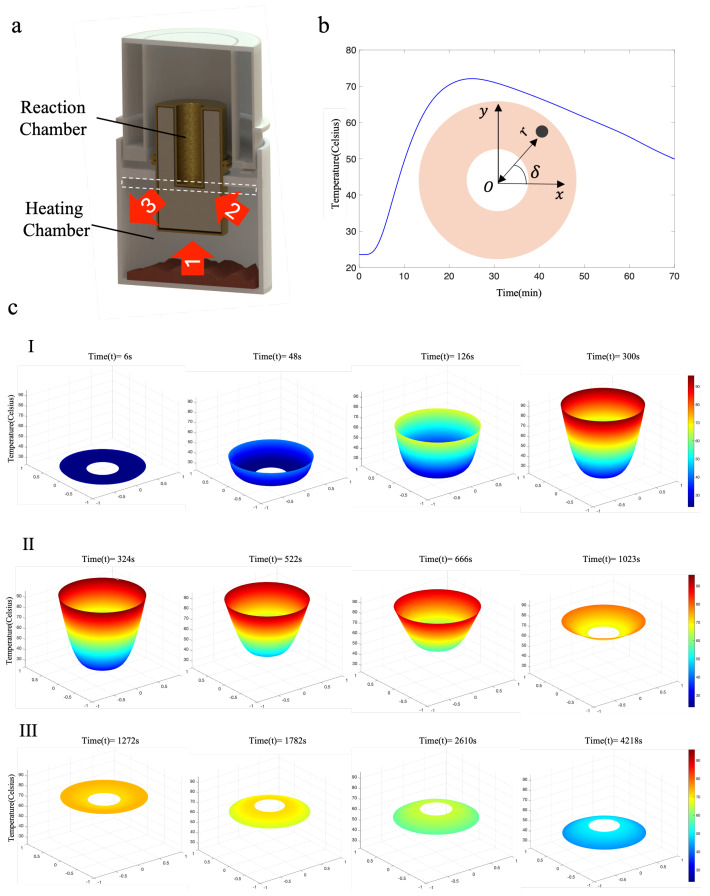
Thermal transfer simulation model. (**a**) During the heating process, heat is continuously transferred to the amplification chamber through exothermic reactions and phase-change materials. When the temperature exceeds the set temperature of the amplification chamber, the phase-change material absorbs excess heat to maintain a constant temperature inside the chamber. After the heating reaction is completed, the heat in the heating chamber dissipates slowly. When the temperature drops below the set temperature of the amplification chamber, the phase-change material releases heat. The entire process allows the amplification chamber to maintain a constant temperature for a long time, meeting the requirements for virus amplification reactions. (**b**) A simulation graph showing the temperature variation of the cylindrical model and the reaction chamber. (**c**) A three-dimensional schematic of the internal heat variation in the equipment.

In this section, a heat transfer model is described to simulate the temperature variations during the reaction process in the device. By utilizing this simulation model, we can reduce the number of subsequent experimental measurements, enabling us to rapidly determine the optimal droplet size for water deposition design [42]. As shown in Figure 3b, for a cylindrical geometry model, the heat transfer model can be described as follows:

(1) $$\frac{dT}{dt}=\sigma \nabla^{2}T=\sigma \left(\frac{1}{r}\frac{\partial }{\partial r}\left(r\frac{\partial T}{\partial r}\right)\right)+\frac{1}{r^{2}}\frac{\partial^{2}T}{\partial \delta^{2}}$$

where *r* and δ are described in polar coordinates, with *r* representing the distance between a point inside the cylinder and the center of the cylinder and δ representing the angle with respect to the *x*-axis. σ represents the thermal conductivity of the cylinder material. To solve the heat transfer model described by the partial differential equation mentioned above, we use the finite difference method to calculate the temperature distribution inside the cylinder over time. The detailed difference method is as follows:

(2) $$\frac{\partial T}{\partial r}=\frac{T(p+1,f,s)-T(p,f,s)}{\Delta r}$$

(3) $$\frac{\partial^{2}T}{\partial r^{2}}=\frac{T(p+1,f,s)-2T(p,f,s)+T(p-1,f,s)}{\Delta r^{2}}$$

(4) $$\frac{\partial^{2}T}{\partial \delta^{2}}=\frac{T(p,f+1,s)-2T(p,f,s)+T(p,f+1,s)}{\Delta \delta^{2}}$$

where *s* represents the serial number of time steps in the finite difference method, *p* represents the serial number of steps in the *r* direction, and *f* represents the serial number of steps in the δ direction. Finally, the numerical model of the system can be represented as follows:

(5) $$\begin{array}{c} T_{p,f,s+1}=T_{p,f,s}+\frac{\sigma \Delta t}{\Delta r^{2}}\left(T_{p+1,f,s}-2T_{p,f,d}+T_{p-1,f,s}\right)+\frac{\sigma \Delta t}{r_{l}\Delta r}\left(T_{p+1,f,s}-T_{p,f,s}\right)\\ +\frac{\sigma \Delta t}{r_{l}^{2}\Delta \delta^{2}}\left(T_{p,f+1,s}-2T_{p,f,s}+T_{p,f-1,s}\right) \end{array}$$

where Δt represents the time step, which is set to 0.01 s in the simulation. Δr represents the step size in the *r* direction, and Δδ represents the step size in the δ direction. Assuming the inner radius of the cylinder is around 3 mm and the outer radius is around 8 mm, we set up 10 grid points in the *r* direction and 45 grid points in the δ direction. The boundary conditions for the model are as follows: the initial temperature at the inner wall is 23 °C (room temperature), and the temperature at the outer wall is based on experimental measurements. Finally, we solve for the temperature distribution inside the cylinder as the temperature at the outer wall varies. The resulting temperature distribution plot inside the cylinder is shown in Figure 3c.

The entire heat transfer process can be divided into three stages. In the first stage, the initial reaction period is from 0 to 5 min. The heat reaction packet generates heat, causing the overall temperature of the heating chamber to rise. At the same time, heat is gradually transferred from the amplification chamber, which undergoes a phase change, to the reaction tube. The temperature of the heating chamber reaches 90 °C within the first 5 min of the reaction. This stage is illustrated in Figure 3c(I). In the second stage, the mid-reaction period is from 5 to 20 min, and the phase-change material starts absorbing heat due to thermal coupling, while the temperature of the external heating chamber gradually decreases. After about 20 min, the temperatures of the heating chamber and the amplification chamber reach equilibrium at 70 °C. This stage is depicted in Figure 3c(II). The third stage is the late reaction period from 21 to 70 min. As the exothermic reaction gradually subsides, heat transfers from the heating chamber to the outer boundary, causing the temperature of the heating chamber to decrease. At the same time, the phase-change material begins to release heat into the heating chamber. Ultimately, at 70 min, the temperature of the amplification chamber decreases to 50 °C, while the temperature of the heating chamber drops to 40 °C. This stage is shown in Figure 3c(III).

To validate the effectiveness of the water flow rate for the heat reaction and temperature control, we designed a set of comparative experiments to examine the temperature changes in the amplification chamber. We used a thermocouple (Keithley 2110 5 1/2) (Beaverton, OR, USA) to measure the temperature of the reaction tube. The reaction tube was placed inside the amplification chamber, and water was used instead of the reaction reagent in the tube. The tube lid had a hole with a diameter of 0.6 mm, allowing the thermocouple to be inserted. Before conducting the experiments, we calibrated the readings of the thermocouple using a kerosene thermometer and ensured the accuracy of the calibration. In the experiment, we used regular tap water and a food heating package (Zhejiang Xindan Industry and Trade Co., Ltd.) (JInhua, China) consisting of CaO-Al and Na_2_CO_3_. The reaction of CaO-Al, Na_2_CO_3_, and water facilitates sustained heat release. This is widely used in food heating and does not produce any toxic substances. The reaction process is as follows:

(6) $$\mathrm{CaO}+H_{2}O=\mathrm{Ca}\left({\mathrm{OH})}_{2}\right.$$

(7) $$\mathrm{Ca}\left({\mathrm{OH})}_{2}+{\mathrm{Na}}_{2}{\mathrm{CO}}_{3}={\mathrm{CaCO}}_{3}↓+2\mathrm{NaOH}\right.$$

(8) $$\left.2\mathrm{AI}+2H_{2}O+2\mathrm{NaOH}=2{\mathrm{NaAIO}}_{2}+3H_{2}↑\right.$$

**Figure 4:**
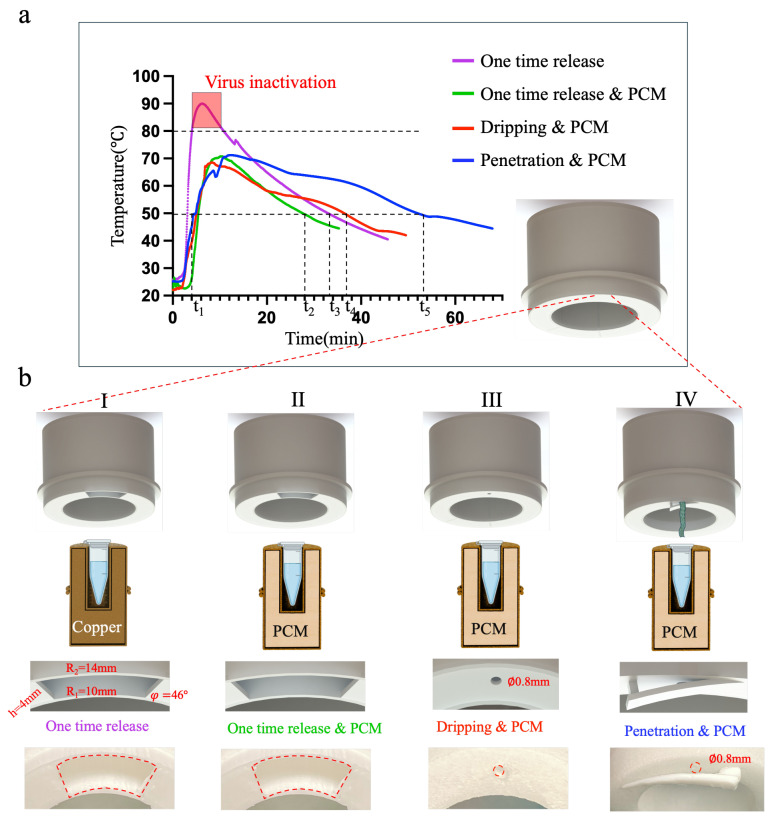
Temperature variation of the amplification chamber under four different conditions. (**a**) Experimental temperature variation curves of the reaction chamber under four different conditions: one-time release, one-time release and PCM, dripping and PCM, and penetration and PCM. (**b**) Structural changes and physical illustrations of the equipment under the four different conditions.

In the first group, we initially used a larger annular sector ($R_{1}=10$ mm, $R_{2}=14$ mm, h=4 mm, $\varphi =46^{\circ }$) as the water outlet, as shown in Figure 4b(I). This was combined with a pure copper amplification chamber (without the phase-change material), and the temperature changes during the reaction process were measured. The temperature variations are represented by the purple curve in Figure 4a. It was observed that the maximum temperature exceeded 80 °C, which would directly inactivate the virus and prevent effective amplification. The overall temperature dropped below 50 °C after 34 min $\left(t_{3}\right)$. In the second group, we used the same-sized water outlet as in the first group, as shown in Figure 4b(II). This was combined with an amplification chamber containing the phase-change material, and the temperature changes throughout the process were measured. The temperature variations are depicted by the green curve in Figure 4a. The maximum temperature reached 70 °C, and the overall temperature dropped below 50 °C after 28 min $\left(t_{2}\right)$. The effective temperature duration was $t_{2}-t_{1}=24$ min. Due to the incorporation of the phase-change materials, the overall effective time has increased, while the maximum temperature has been effectively suppressed, demonstrating the regulating effect of the phase-change material on temperature. However, the effective temperature duration was still too short, resulting in insufficient amplification for samples with low copy numbers and inaccurate detection results. In the third group, we used a water outlet with a diameter of ∅0.5 mm, as shown in Figure 4b(III), combined with an amplification chamber containing the phase-change material. The temperature changes throughout the process were measured, represented by the red curve in Figure 4a. It can be observed that the maximum temperature reached 68 °C, and the effective reaction time was $t_{4}-t_{1}=33$ min. In the fourth group, we utilized a water outlet with a diameter of ∅0.5 mm, combined with chromatographic cotton for controlled release. This configuration, as shown in Figure 4b(IV), was paired with an amplification chamber containing phase-change material. The temperature changes during the process were measured, and the temperature variation is represented by the blue curve in Figure 4a. The maximum temperature reached 70 °C, and the effective reaction time was determined to be $t_{5}-t_{1}=49.8$ min.

The results indicate that by adjusting only the water flow rate in the amplification chamber containing the same phase-change material, the effective temperature duration in the third group was extended by 9 min compared to the second group experiment with one-time water release. In the fourth group, the effective temperature duration was extended by 16.8 min compared to the third group experiment without chromatographic-cotton-controlled release and by 25.8 min compared to the second group experiment with one-time water release. This provides ample evidence that controlling the water flow rate can further regulate the reaction duration, allowing for sustained exothermic reactions and achieving temperature consistency even without insulation shells.

## 3. Virus Experiments

**Figure 5:**
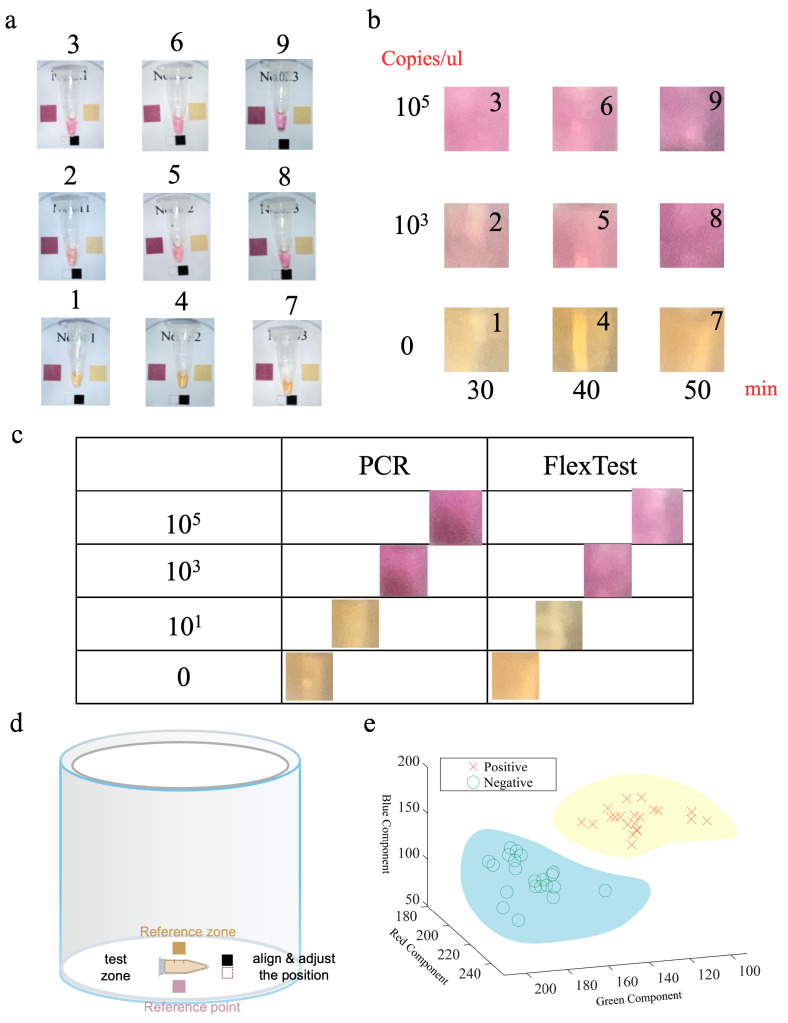
Experimental results with pseudovirus. (**a**) Real images of nine samples after reaction in the device. (**b**) Arrangement and comparison of the results for the nine samples based on gradient and reaction time. (**c**) Comparison of the reaction results between the portable reaction device and the professional PCR device (ProFlex PCR 3 × 32). (**d**) Illustration of the photographed light-shielding cover (device packaging) and colorimetric card. (**e**) Region plots of positive and negative data after machine learning training.

To further verify the reliability and accuracy of the device, we conducted a COVID-19 pseudovirus virus experiment. We used a visual LAMP reaction reagent kit (COVID-19 N-Gene Visualization Nucleic Acid Test Kit, Beijing Mylab Medical Science and Technology Co., Ltd., Beijing, China) specifically for a performance verification of the LAMP constant-temperature amplification equipment. A positive reaction result with this reagent kit is pink, negative is yellow, and a failed reaction is translucent and turbid. The minimum effective concentration in a positive control group for this reagent is $10^{3}$ copies/μL. Therefore, in the experiment, we used a minimum virus concentration of $10^{3}$ copies/μL. We added virus samples with gradients of 0, $10^{3}$, and $10^{5}$ copies/μL to the reaction tubes containing the reaction system and initiated the reaction using the device. The reaction times were 30, 40, and 50 min. As shown in Figure 5a,b, the three negative samples (with a gradient of 0 copies/μL and sample numbers 1, 4, and 7) exhibited a yellow color at different time points of 30, 40, and 50 min, and the intensity of the yellow color increased with the reaction time. The three positive samples (with a gradient of $10^{3}$ copies/μL and sample numbers 2, 5 and 8) exhibited a pink color at different time points of 30, 40, and 50 min, and the intensity of the pink color increased with the reaction time. Similarly, the three positive samples (with a gradient of $10^{5}$ copies/μL and sample numbers 3, 6, and 9) exhibited a pink color at different time points of 30, 40, and 50 min, and the intensity of the pink color increased with the reaction time. Additionally, comparing the results of the reactions at the same time point for gradients of $10^{3}$ and $10^{5}$ copies/μL, such as samples 2 and 3, 5 and 6, and 8 and 9, the pink color observed in the $10^{5}$ copies/μL gradient was darker than that in the $10^{3}$ copies/μL gradient. This not only demonstrates the effectiveness of the device but also confirms that the device can complete the required amplification reaction within the 30–50 min time range to achieve the desired detection effect. Taking into account the virus amplification efficiency, detection effect, and user experience, we set the response time standard to 50 min. To further verify the consistency with professional gold-standard equipment, we added the samples of the four gradients to the reaction system and put them into the device we designed and the commercial PCR equipment (ProFlex PCR 3 × 32) for a 50 min reaction. The experimental results are shown in Figure 5c. It was found that at the $10^{3}$ gradient and the $10^{5}$ copies/μL gradient, the results of our device were consistent with those of the professional PCR equipment. In detail, the color concentration displayed after the reaction was almost the same, and all showed as positive. The color for the 0 gradient and the 10 copies/μL gradient showed as negative, which is the same as the result wwih the professional PCR equipment. These results further prove the reliability and accuracy of our equipment.

## 4. Result Analysis Using the Mobile App

Because of its portability, our device can be applied in remote areas with limited medical facilities. However, most people there are not trained in PCR operations. To make our device user-friendly, we further standardized and normalized the discrimination of the test results and enabled these results to be uploaded. To address this, we designed a mobile application that allows users to collect and analyze the test result using their own smartphone. This makes it easy for individuals, including those with color vision impairments, to accurately identify and save the test results through the application, ensuring precise recognition and record-keeping.

### 4.1. Color Calibration Using the Color Card

To mitigate the impact of different phone models on color recognition, we designed a camera calibration system, as shown in Figure 5d, along with a corresponding calibration algorithm. The result display unit is designed as a circle tube with a diameter of 30 mm, which is more suitable for image capture compared to square or rectangular shapes. At the bottom of the tube, there are fixed-size black and white blocks that aid in adjusting the position of the test results within the camera frame [43]. On the left and right sides of the test tube, there are fixed-size positive control color blocks (in pink) and negative control color blocks (in yellow). These four color blocks are used together to calibrate the colors of the captured pictures. By comparing the captured colors of the reference color blocks with the standard colors, the color calibration algorithm can determine color distortion and deviation in results from different phone models and lighting conditions. As the images are modeled using RGB, the calibration parameters are defined as follows:

(9) $$\Theta ={\left[\begin{array}{cccccc} \theta_{r1} & \theta_{r2} & \theta_{g1} & \theta_{g2} & \theta_{b1} & \theta_{b2} \end{array}\right]}^{⊤}\in R^{6}$$

where $\theta_{r1}$ and $\theta_{r2}$ are the correction parameters for the red component of the image, $\theta_{g1}$ and $\theta_{g2}$ are for the green component, and $\theta_{b1}$ and $\theta_{b2}$ are for the blue component. The color vector of the original test result is denoted as $C=\left[\begin{array}{ccc} R & G & B \end{array}\right]$; then, the corrected color vector of the test result is as follows:

(10) $$\hat{C}=\left[\begin{array}{c} \hat{R}\\ \hat{G}\\ \hat{B} \end{array}\right]=\left[\begin{array}{cccccc} R & 1 & 0 & 0 & 0 & 0\\ 0 & 0 & G & 1 & 0 & 0\\ 0 & 0 & 0 & 0 & B & 1 \end{array}\right]\Theta$$

As for the four reference color blocks, the color components of the captured reference color are denoted as $R_{i},G_{i},B_{i},i=1,2,3,4$; then, the corrected captured reference color vector is as follows:

(11) $$\tilde{C}=\left[\begin{array}{cccccc} R_{1} & 1 & 0 & 0 & 0 & 0\\ 0 & 0 & G_{1} & 1 & 0 & 0\\ 0 & 0 & 0 & B_{1} & 1 & 0\\ 0 & 0 & 0 & 0 & B_{2} & 1\\ R_{2} & 1 & 0 & 0 & 0 & 0\\ 0 & 0 & G_{2} & 1 & 0 & 0\\ 0 & 0 & 0 & 0 & B_{2} & 1\\ R_{3} & 1 & 0 & 0 & 0 & 0\\ 0 & 0 & G_{3} & 1 & 0 & 0\\ 0 & 0 & 0 & 0 & B_{3} & 1 \end{array}\right]\Theta =\Omega \Theta$$

where Ω is the matrix containing all the color components of the captured reference color.

Then, the search for the optimal correction parameters can be converted into an optimization problem, where the objective function is the sum of the squares of the correction errors, as follows:

(12) $$J\left(\theta \right)=\|\tilde{C}-C_{s}{\|}^{2}={\left(\Omega \Theta -C_{s}\right)}^{T}\left(\Omega \Theta -C_{s}\right)$$

where $C_{s}$ is the standard color vector, which can be formulated by the color components of the standard colors as follow

(13) $$C_{s}={\left[\begin{array}{ccc} C_{s1} & C_{s2} & C_{s3} \end{array}\right]}^{⊤},C_{si}=\left[\begin{array}{ccc} R_{si} & G_{si} & B_{si} \end{array}\right],i=1,2,3,4$$

Then, the derivative of the objective function can be calculated as follows:

(14) $$\frac{\partial }{\partial \Theta }J\left(\Theta \right)=2\Omega^{⊤}\Omega \Theta -2\Omega^{⊤}C_{s}=0$$

Setting the derivative to 0, the optimal correction parameters can be obtained as follows:

(15) $$\Theta ={\left(\Omega^{⊤}\Omega \right)}^{-1}\Omega^{⊤}C_{s}$$

With the optimal calibration vector Θ, the original sample color can be converted into the correct color of the sample to be tested through Equation (10), ensuring that the result is consistent regardless of which camera model the smartphone used for capture.

### 4.2. Data Analysis and Discrimination

Within our smartphone application, we have implemented a color discrimination algorithm to analyze the test results. The corrected test results are a three-dimensional vector, denoted as $\tilde{d}\in R^{3}$. We have constructed a discrimination model, denoted as $G_{w}$, to determine whether the results indicate a positive outcome. This discrimination model is a linear model determined by a parameter vector *w*. The discrimination result $R\left(\tilde{d}\right)\in \left\{0,1\right\}$ can be obtained using the following equation:

(16) $$R\left(\tilde{d}\right)=\mathrm{sign}\left(G_{w}\left(\tilde{d}\right)\right)$$

where $R(\tilde{d})=0$ is for negative results, and $R(\tilde{d})=1$ is for positive results.

To achieve optimal discrimination performance, we built a dataset *D* to train the discrimination model. The dataset includes test result data $S\in R^{3}$ and labels $L\in \left\{0,1\right\}$, where 0 and 1, respectively, represent negative and positive. The training of the model can be expressed as solving the following optimization problem:

(17) $$\begin{array}{cc} \; & \min_{w}\;\frac{1}{2}w^{T}w+c\sum_{i=1}^{n}\varepsilon_{i}\\ s.t.\; & l_{i}\left(w^{T}d_{i}\right)\geq 1-\varepsilon_{i},i=1,\ldots ,n\\ \; & \varepsilon_{i}\geq 0 \end{array}$$

where $d_{i}\in D,\;l_{i}\in L$. The penalty parameter *c* and the slack variable ε ensure that the model can be trained in situations with outliers.

The trained model can divide the data space of the test results into a positive data part and a negative data part. As shown in Figure 5e, the test results fall into two different regions, corresponding to their actual labels accurately. The test results that are actually negative are marked by green circles, while the actual positive results are marked by red crosses. The trained discriminant model divides them into two parts, clustering all the positive test results into a positive region (yellow part) and all the negative test results into a negative region (blue part). In this way, each test result can be accurately determined, and then the results will be directly presented in text form as positive or negative on the app.

## 5. Demonstration of the Whole Test Process 

**Figure 6:**
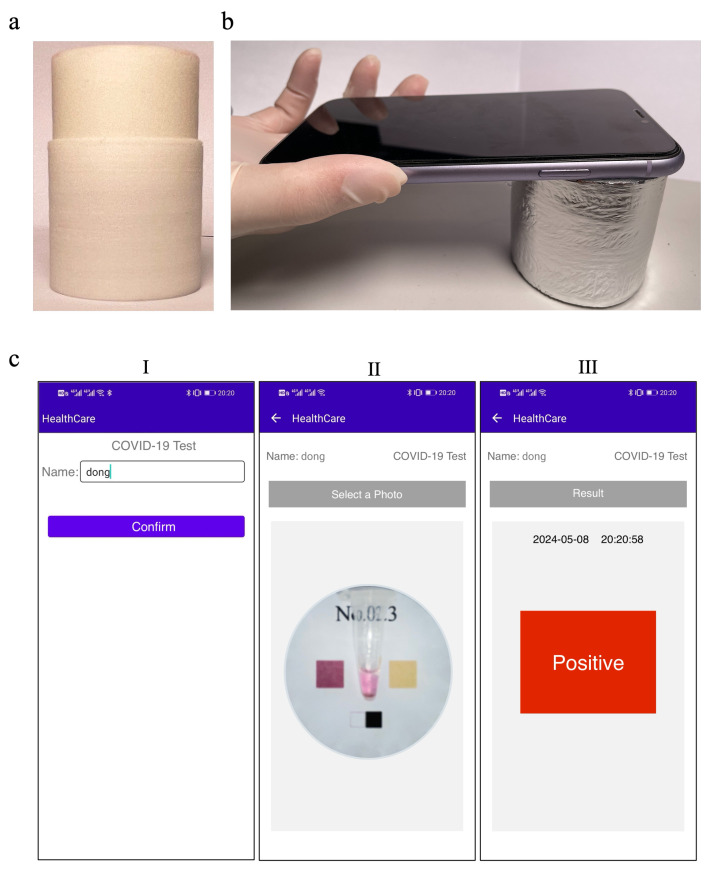
Detection data management system based on mobile app. (**a**) A physical illustration of the portable reaction device. (**b**) After the reaction, the test tube is placed in the bottom of the packaging box for photography. (**c**) User interface of the accompanying mobile app, consisting of three pages: name input, photo uploading, and result display.

During the usage process for the device, the user is required to collect a respiratory sample (in our experiment, a COVID-19 pseudovirus virus is used) using a swab. The sample is then placed into the reaction tube and covered with the tube cap. The tube is inserted into our portable testing device and covered with the lid, allowing the reaction to proceed normally. After waiting for 50 min, the tube is removed and placed in the middle of the color chart at the bottom of the outer packaging box. The outer packaging box plays the role of blocking light, reducing interference from external light sources. The light needed for photography is provided by the built-in flashlight of the mobile phone. The user places their phone directly above the tube, as shown in Figure 6b, and adjusts the focus distance to capture a clear image of the entire color chart (we use a 0.5× focus distance for capturing the image in the experiment). Subsequently, the user opens the mobile app terminal, enters the name of the individual being tested, as shown in Figure 6c(I), and clicks on Confirm. The app then navigates to the second page, as depicted in Figure 6c(II), where the user selects the captured image. The app further proceeds to the third page, which is the test result page, shown in Figure 6c(III). This page displays the current test results and enables users to save a record. Multiple tests can be conducted on different individuals within a certain period, and past test records can be accessed, as shown in Figure 1a(VI).

## 6. Results and Discussion

Through heat transfer experiments and simulations, we have demonstrated the feasibility of maintaining isothermal amplification by controlling the heat reaction rate. We have proposed a chromatographic-strip-based microporous permeation structure that can achieve the amplification temperature requirements of 50–70 °C within 4 min and sustain them for 49.8 min. This power-free and controllable heat reaction not only significantly reduces the inherent volume of the detection equipment but also lowers the operational threshold for users. Comparative experiments were conducted using SARS-CoV-2 pseudovirus samples and the gold-standard LAMP assay. The detection results for the four virus gradients were consistent, with a limit of detection (LOD) of $10^{3}$ copies/μL, which demonstrated the effectiveness of our device. The device is also equipped with a mobile application for result analysis and data management. By utilizing a colorimetric card and a corresponding color calibration algorithm, it is possible to address color variations caused by different smartphone models. The color discrimination algorithm enhances the accuracy of the result analysis. Furthermore, by uploading the detection results to the database to save the records, it is convenient to check and manage health data during disease epidemic periods, predict individual infection status and group virus infection trends, and help in virus prevention and control.

## 7. Summary and Future Work

In the context of frequent infections caused by respiratory viruses in humans and animals, timely detection of the viral type holds significant implications for subsequent treatments. In this work, we designed a device to detect a virus. In this device, a portable constant-temperature amplification device is designed innovatively to controls the water flow speed by using chromatographic cotton, thereby controlling the exothermic reaction speed. It replaces the scheme of maintaining the temperature with a thermos and realizes a lower cost, a smaller size, and reusability. However, the current device is unable to perform real-time detection of virus amplification, thus preventing the achievement of quantitative measurements. In future work, we will integrate portable virus purification techniques to improve the sensitivity and accuracy of the device. At the same time, by recording videos and reading the color change in the test tube in real time, we can turn qualitative detection into real-time detection, further improving the performance of the device. In the upper cover we designed, there is a mechanism to adjust the water flow rate. In future work, this can be used to address different extreme environmental temperatures and various constant-temperature amplification technologies (such as Recombinase Polymerase Amplification, etc.) to regulate the water flow rate, thereby adjusting the temperature maintained during the reaction process. This will enhance the overall adaptability of the equipment.

## Figures and Tables

**Table 1:** A comparative analysis of portable thermocycler devices from recent years.

Name	Samples	Amplification Method	Heating Mechanism	Power Supply	LOD	Reuse	Insulation	Ref.
Portable device for LAMP	SARS-CoV-2	LAMP	Cartridge heater	Yes	$10^{2}$ copies per oro-nasopharyngeal swab	Yes	No	[29]
Electro mechanical RT-LAMP	SARS-CoV-2	LAMP	Peltier heaters	Yes	$10^{3}$ copies/mL	Yes	No	[31]
uMED	SARS-CoV-2	RPA	Electrochemical applications	Yes	0.040 ng/μL	Yes	No	[32]
Mi SHERLOCK	SARS-CoV-2	CRISPR	Polyimide heaters	Yes	1240 cp/mL	Yes	No	[33]
Wearable RPA	HIV-1	RPA	Human body heat	Yes	$10^{3}$–$10^{5}$ copies/mL	Yes	No	[34]
EPCM	SARS-CoV-2	LAMP	Mg(Fe)	No	N/A	No	Yes	[24]
Smart cup	HSV-2	LAMP	Mg-Fe	No	50 copies/reaction	Yes	Yes	[35]
Our device	SARS-CoV-2	LAMP	CaO-Al, Na_2_CO_3_	No	$10^{3}$ copies/μL	Yes	No	N/A

## Data Availability

Data are contained within the article and Supplementary Materials.

## Supplementary Materials

The following supporting information can be downloaded at: https://www.mdpi.com/article/10.3390/s24154912/s1.
