# Supplementary material for: An Ultra-Compact and Low-Cost LAMP-Based Virus Detection Device
Source: Sensors (Basel). 2024 Jul 29;24(15):4912. doi: 10.3390/s24154912 (PMC11314854; doi:10.3390/s24154912)
Supplement: Supplementary file 1 [file sensors-24-04912-s001.zip › Dripping & PCM .pdf]

Number	Time(s)	Temperature (° C)
1	0.0007641	22.1864871
2	1.2046433	22.0960578
3	2.408599	22.0828227
4	3.6123396	22.0685577
5	4.8252472	22.0948295
6	6.0287606	22.1137828
7	7.2327901	22.1089134
8	8.4368716	22.1257362
9	9.6399149	22.1219387
10	10.8441422	22.1227073
11	12.0479399	22.1236343
12	13.3004169	22.1586952
13	14.5040912	22.1672019
14	15.7076043	22.1391067
15	16.9122519	22.1282234
16	18.1173497	22.0817985
17	19.3218098	22.0557041
18	20.5266754	22.109188
19	21.7310909	22.1410522
20	22.9361241	22.1040992
21	24.1406393	22.1066322
22	25.3459207	22.1535625
23	26.5505022	22.1931362
24	27.7544693	22.27145
25	28.9587252	22.2915687
26	30.1634571	22.3641242
27	31.3685755	22.4201812
28	32.5731259	22.341793
29	33.7777421	22.3740234
30	34.9825401	22.3897361
31	36.1877328	22.376564
32	37.3924995	22.2822914
33	38.5980593	22.2705879
34	39.8028479	22.2860374
35	41.0072525	22.2893695
36	42.2121886	22.2386589
37	43.4163973	22.2437171
38	44.6214986	22.3213367
39	45.8261597	22.324728
40	47.0319468	22.4058589
41	48.2362688	22.4732627
42	49.4417343	22.5674457
43	50.6463724	22.6106262
44	51.8499211	22.6293811
45	53.0546685	22.6814937
46	54.259638	22.7697544
47	55.4639265	22.8132057
48	56.6683436	22.817871
49	57.8715142	22.8163986

50	59.0756182	22.8330364
51	60.2801406	22.842678
52	61.4846152	22.8216667
53	62.6887618	22.8083209
54	63.8928356	22.7681331
55	65.0967807	22.7439575
56	66.3016811	22.7142295
57	67.5068543	22.6785392
58	68.7104174	22.6869964
59	69.9150275	22.6927318
60	71.1200355	22.6209564
61	72.3243106	22.5184688
62	73.5285049	22.5467014
63	74.7325694	22.4925651
64	75.936299	22.4905033
65	77.1407877	22.4519424
66	78.3455818	22.5000991
67	79.5493788	22.5400981
68	80.7540825	22.5392951
69	81.9581492	22.5456695
70	83.1621093	22.6222267
71	84.3649735	22.7401618
72	85.5694934	22.7562656
73	86.7740209	22.8443603
74	87.9772571	22.9060268
75	89.1819326	22.9819278
76	90.3859243	22.9733085
77	91.5906399	22.9722824
78	92.7954063	22.9717407
79	93.9996854	22.963581
80	95.2035974	22.9476509
81	96.4074671	22.9164638
82	97.6116117	22.88031
83	98.8148246	22.8435974
84	100.0185405	22.8128528
85	101.2228818	22.7843933
86	102.4269471	22.7605094
87	103.6308297	22.7407627
88	104.8354888	22.7153301
89	106.0407201	22.7036972
90	107.2457766	22.7024192
91	108.4492252	22.7087345
92	109.6534205	22.7282867
93	110.8578645	22.7469272
94	112.0701543	22.7638435
95	113.2742368	22.7917747
96	114.479471	22.8322925
97	115.6842364	22.8860569
98	116.8884278	22.951992
99	118.0927425	23.02709

100	119.2972636	23.1025791
101	120.5020189	23.1948966
102	121.7062946	23.2862339
103	122.9099699	23.3932991
104	124.1136825	23.5089054
105	125.3175115	23.6429557
106	126.5213365	23.7985591
107	127.7255211	23.937397
108	128.929653	24.0713577
109	130.1342931	24.2086067
110	131.3380175	24.3639736
111	132.5429662	24.5494232
112	133.7473383	24.7220001
113	134.9522811	24.8763523
114	136.1554507	25.0334205
115	137.3599784	25.1840019
116	138.5648611	25.3171119
117	139.7694035	25.475605
118	140.9737604	25.6551094
119	142.178322	25.8408241
120	143.3816285	26.0205135
121	144.5863031	26.1767539
122	145.7908301	26.3567295
123	146.9948283	26.5661106
124	148.1995518	26.7813167
125	149.4037354	27.0003852
126	150.6070949	27.2235908
127	151.8120184	27.4475097
128	153.0175191	27.671833
129	154.2208083	27.8956756
130	155.4250452	28.1182708
131	156.6300608	28.3421573
132	157.8344288	28.5675926
133	159.0394331	28.8617668
134	160.2433673	29.1665649
135	161.4475049	29.4700355
136	162.6515013	29.7831153
137	163.8552749	30.1125888
138	165.0593855	30.4222145
139	166.2645074	30.7350826
140	167.4697597	31.0411891
141	168.6747504	31.3723602
142	169.8786978	31.6928596
143	171.082647	31.9415283
144	172.2874668	32.1587295
145	173.4915851	32.3822555
146	174.6963811	32.5981369
147	175.9017142	32.7850456
148	177.1064404	32.9820632
149	178.3115672	33.1608695

150	179.5169562	33.3386116
151	180.7210644	33.4743156
152	181.9258844	33.6131515
153	183.1302793	33.7326545
154	184.3344955	33.8612556
155	185.5398293	33.9887161
156	186.7444143	34.1236419
157	187.9483868	34.2534484
158	189.1530188	34.3820915
159	190.3564357	34.5141868
160	191.5606603	34.6834831
161	192.7658441	34.8593025
162	193.9697549	35.0344314
163	195.1740726	35.2232971
164	196.3772351	35.4080924
165	197.5811541	35.5771293
166	198.7856373	35.7440223
167	199.9898802	35.9061126
168	201.1949746	36.0609397
169	202.4010759	36.208641
170	203.6059123	36.3167457
171	204.8116563	36.4158973
172	206.015751	36.5092658
173	207.2206903	36.5890884
174	208.4258795	36.6797103
175	209.6298768	36.776596
176	210.8340844	36.8797988
177	212.0389011	36.9910697
178	213.2427338	37.0962448
179	214.4476592	37.1981506
180	215.6536409	37.2974433
181	216.858288	37.3970413
182	218.0627678	37.4935264
183	219.2673895	37.6033096
184	220.4719622	37.7018356
185	221.6774614	37.788166
186	222.8813316	37.8468894
187	224.0852	37.9083328
188	225.2895577	37.9819679
189	226.4931854	38.0659065
190	227.6974203	38.1543197
191	228.902096	38.2494544
192	230.1061746	38.3488998
193	231.3109555	38.4451637
194	232.5149416	38.5481452
195	233.718491	38.6619186
196	234.9222948	38.7797889
197	236.1264005	38.9001235
198	237.3307938	39.0235557
199	238.5349499	39.146862

200	239.7394455	39.2711257
201	240.9435748	39.4002113
202	242.1480084	39.5331649
203	243.3535002	39.6689376
204	244.5565209	39.8139877
205	245.7607803	39.9622879
206	246.9651112	40.1193046
207	248.1700719	40.2781143
208	249.3745227	40.4410629
209	250.5801088	40.6111259
210	251.7840583	40.7850303
211	252.9891345	40.959526
212	254.1940277	41.1381988
213	255.3989724	41.3201789
214	256.6029208	41.49971
215	257.8074396	41.6812515
216	259.0107057	41.8616256
217	260.2150392	42.0468711
218	261.4195953	42.2339553
219	262.6247403	42.4223175
220	263.8286802	42.6127967
221	265.0327432	42.8062286
222	266.2372805	42.9994087
223	267.4424004	43.1934089
224	268.6470173	43.3884735
225	269.8517349	43.5890045
226	271.0560433	43.7951545
227	272.2599558	44.048645
228	273.4644922	44.3365249
229	274.6694341	44.6261138
230	275.8731415	44.9108047
231	277.0780094	45.1925621
232	278.2819539	45.4758377
233	279.4862603	45.760437
234	280.6911618	46.0459251
235	281.8951015	46.3301086
236	283.1058996	46.6121292
237	284.3103598	46.8502006
238	285.5135948	47.0569839
239	286.7176558	47.265625
240	287.9223747	47.4836044
241	289.1270989	47.7080612
242	290.3317803	47.9347305
243	291.536488	48.1623878
244	292.7416185	48.3908309
245	293.9457133	48.6170272
246	295.1507509	48.8402481
247	296.3552682	49.0609207
248	297.5600389	49.2795906
249	298.764327	49.4959716

250	299.9680904	49.7074966
251	301.1728417	49.9145088
252	302.3784438	50.1182174
253	303.5839186	50.3180809
254	304.7876269	50.5135421
255	305.9920008	50.7063217
256	307.1972062	50.8963928
257	308.4024692	51.0842285
258	309.6068639	51.2692947
259	310.8115893	51.4509162
260	312.0155669	51.6315689
261	313.2202514	51.808937
262	314.4252376	51.9827957
263	315.630199	52.1536178
264	316.8343279	52.3235359
265	318.0393741	52.4904174
266	319.2432911	52.6549072
267	320.4481397	52.8167839
268	321.6527244	52.9756698
269	322.8569415	53.1317024
270	324.06244	53.2853469
271	325.2662807	53.4370346
272	326.4703824	53.5886039
273	327.6750235	53.7387466
274	328.8798936	53.8869514
275	330.0849596	54.0350112
276	331.2884649	54.1820869
277	332.4924897	54.326416
278	333.6966408	54.4693908
279	334.9004177	54.6107902
280	336.1038623	54.7478523
281	337.3076796	54.8831787
282	338.5113976	55.0137214
283	339.7151688	55.1413345
284	340.9195551	55.2655029
285	342.123727	55.3865776
286	343.3278633	55.508789
287	344.5329227	55.6354751
288	345.7368092	55.7588119
289	346.9410604	55.8763732
290	348.1457632	55.9923019
291	349.3507376	56.1013374
292	350.5552818	56.207405
293	351.7606382	56.3118057
294	352.9642822	56.4118347
295	354.1691118	56.5076484
296	355.3738554	56.6718673
297	356.5780885	56.8113899
298	357.7822489	57.0396308
299	358.9862823	57.260601

300	360.1908136	57.4786605
301	361.3955659	57.6980209
302	362.6001386	57.9154129
303	363.8048493	58.1305236
304	365.010864	58.3440933
305	366.2148415	58.5547866
306	367.4193735	58.6843261
307	368.6239101	58.822071
308	369.827761	58.8615608
309	371.031859	58.9304885
310	372.2366522	58.9726142
311	373.4412734	59.0146713
312	374.6453373	59.0826492
313	375.850306	59.1895446
314	377.0558416	59.3139343
315	378.2599704	59.4175491
316	379.4645771	59.5856399
317	380.6696001	59.6981124
318	381.8744192	59.8314819
319	383.0783529	59.9749984
320	384.2834806	60.1130065
321	385.4883866	60.1810836
322	386.6922367	60.2116203
323	387.8976122	60.1947517
324	389.1036558	60.1631774
325	390.3080871	60.1520462
326	391.5129868	60.0940055
327	392.7172369	60.1348953
328	393.9209077	60.2223625
329	395.1254123	60.2968635
330	396.3302947	60.4187507
331	397.5362643	60.6566047
332	398.7411198	61.0323181
333	399.9460213	61.6689872
334	401.1511459	62.3657417
335	402.3557707	63.0715827
336	403.5605122	63.7656326
337	404.7657366	64.4241638
338	405.9698907	65.0318832
339	407.1735386	65.6218414
340	408.3777269	66.1995315
341	409.5818302	66.7057266
342	410.7868441	67.0707244
343	411.9910618	67.1684417
344	413.1947349	67.1876525
345	414.3990353	67.1795501
346	415.6039724	67.1752853
347	416.8089994	67.159317
348	418.0137485	67.1185302
349	419.2183826	67.0625534

350	420.422571	67.0029602
351	421.6263602	66.9621582
352	422.8310973	66.9423065
353	424.0364498	66.9382095
354	425.2410555	66.9576416
355	426.4450879	66.9954376
356	427.6490704	67.029396
357	428.8522605	67.0709991
358	430.0564071	67.1255035
359	431.2599757	67.1855239
360	432.4642176	67.247055
361	433.6677316	67.3153762
362	434.8730601	67.3886337
363	436.0778294	67.4589385
364	437.2828606	67.5145339
365	438.4880098	67.5727081
366	439.6917617	67.6229705
367	440.8958573	67.67173
368	442.1003569	67.7151641
369	443.305702	67.7666473
370	444.5096049	67.8151855
371	445.7141099	67.8595046
372	446.9177746	67.888153
373	448.1219508	67.9125518
374	449.3267077	67.9396057
375	450.5304807	67.962387
376	451.735385	67.9897003
377	452.9391134	68.0143966
378	454.1430014	68.0408554
379	455.3475345	68.0651702
380	456.5521511	68.0804901
381	457.7577495	68.088768
382	458.9628403	68.0888824
383	460.1673028	68.0907363
384	461.3714442	68.0948028
385	462.5761611	68.1004714
386	463.7808886	68.1081466
387	464.9849211	68.1260223
388	466.1892422	68.1447753
389	467.3928169	68.1645965
390	468.5973654	68.1851196
391	469.8011469	68.2122039
392	471.0055981	68.2380218
393	472.209681	68.2614746
394	473.4129952	68.2846221
395	474.616705	68.3036575
396	475.8207608	68.3138656
397	477.0255542	68.3157196
398	478.2293074	68.3132705
399	479.433636	68.3133087

400	480.6380104	68.3192138
401	481.8430613	68.3269119
402	483.0473656	68.3486938
403	484.2514299	68.3717346
404	485.4566208	68.3988342
405	486.6617346	68.4249114
406	487.8657604	68.4586639
407	489.0709652	68.4921722
408	490.2756599	68.5244598
409	491.4804755	68.5385894
410	492.6852235	68.5487213
411	493.889176	68.553215
412	495.092999	68.5515365
413	496.2976108	68.5549774
414	497.5030043	68.5451583
415	498.7074524	68.5397186
416	499.9119708	68.5277023
417	501.1169078	68.5131225
418	502.321137	68.5055923
419	503.5261268	68.5059661
420	504.7313644	68.507225
421	505.9363444	68.5056533
422	507.1411261	68.5015029
423	508.345901	68.4916992
424	509.5504458	68.4884948
425	510.7556475	68.4791107
426	511.9597867	68.4645309
427	513.1640547	68.4469604
428	514.3677607	68.422966
429	515.5719572	68.3982467
430	516.7767779	68.3752288
431	517.9810768	68.3613357
432	519.1859823	68.3487777
433	520.3904312	68.3353576
434	521.5944774	68.3128738
435	522.7987183	68.2880096
436	524.0027506	68.2743377
437	525.2068858	68.2594146
438	526.4111962	68.2469863
439	527.6162444	68.2348937
440	528.8200139	68.2185897
441	530.0242388	68.1982879
442	531.2288731	68.1730194
443	532.4341586	68.1371612
444	533.6384138	68.1029052
445	534.8420496	68.0624542
446	536.0469253	68.0157012
447	537.2508018	67.9713897
448	538.4545661	67.9232406
449	539.6583083	67.8754043

450	540.8621081	67.8350067
451	542.0660786	67.7940444
452	543.2708248	67.7495269
453	544.4749685	67.7172088
454	545.6796102	67.6905746
455	546.8846231	67.6730194
456	548.0888343	67.6574401
457	549.2928309	67.6376113
458	550.4965374	67.6211242
459	551.7015515	67.6008605
460	552.9064757	67.5676727
461	554.1106824	67.5341033
462	555.3136389	67.5012969
463	556.518221	67.4718856
464	557.7229462	67.448738
465	558.9280233	67.4322509
466	560.1328184	67.413681
467	561.3364508	67.4066085
468	562.5401389	67.4063186
469	563.7448392	67.4054107
470	564.949493	67.4128494
471	566.1537026	67.4116897
472	567.3583636	67.4103546
473	568.5628956	67.4008483
474	569.7678957	67.3835983
475	570.9731256	67.3592681
476	572.1780683	67.3350524
477	573.3835471	67.3053131
478	574.5883442	67.2718276
479	575.792774	67.2412643
480	576.9971714	67.2106552
481	578.2018136	67.1882781
482	579.4058101	67.175148
483	580.6096119	67.16539
484	581.8139369	67.1573562
485	583.0183511	67.1511459
486	584.2226107	67.1474456
487	585.4274868	67.141159
488	586.6324611	67.1311035
489	587.8364765	67.1233749
490	589.0406916	67.1148223
491	590.2442324	67.1068038
492	591.4498362	67.0981063
493	592.6547985	67.0882644
494	593.8593023	67.0782546
495	595.0637428	67.0686645
496	596.2677255	67.0579605
497	597.4720789	67.0511093
498	598.6771004	67.049942
499	599.8826041	67.0509872

500	601.0875856	67.05233
501	602.2915333	67.0524444
502	603.4966605	67.0519561
503	604.7007149	67.0512161
504	605.9051005	67.0508117
505	607.1097965	67.0502471
506	608.3147715	67.0496978
507	609.5182685	67.047554
508	610.7223724	67.0401535
509	611.9273918	67.032341
510	613.1322442	67.0308685
511	614.3363638	67.0295562
512	615.5410569	67.0285797
513	616.744946	67.027481
514	617.9528737	67.0264205
515	619.1572837	67.0250015
516	620.3617981	67.024826
517	621.5660926	67.0284042
518	622.7696093	67.0332031
519	623.9742926	67.0376205
520	625.1780707	67.0365447
521	626.3828884	67.0362091
522	627.5872515	67.0366973
523	628.7913165	67.0372543
524	629.9958525	67.0393295
525	631.1995922	67.0421676
526	632.40409	67.0464248
527	633.6089178	67.0477905
528	634.8134181	67.0476837
529	636.0169151	67.0457534
530	637.22118	67.0437469
531	638.4257409	67.0413742
532	639.6301995	67.0384979
533	640.8347403	67.037384
534	642.0395416	67.0351791
535	643.2439036	67.0331039
536	644.4476743	67.0278854
537	645.6528051	67.0347137
538	646.8580393	67.0422363
539	648.0625276	67.0504913
540	649.2674786	67.0581741
541	650.471488	67.0659332
542	651.6755943	67.0728454
543	652.8809025	67.077629
544	654.0858053	67.0817718
545	655.2906771	67.0854492
546	656.4954227	67.0929031
547	657.7000045	67.08638
548	658.904964	67.0780868
549	660.1101921	67.0694961

550	661.3153934	67.0606079
551	662.5208772	67.0510406
552	663.7253478	67.0401153
553	664.9300768	67.028923
554	666.1349195	67.0173034
555	667.3396815	67.004074
556	668.5439486	66.9859466
557	669.748418	66.9679718
558	670.9514041	66.9508819
559	672.1554778	66.9335708
560	673.3598591	66.9168472
561	674.5643624	66.9001464
562	675.7686891	66.8847198
563	676.9724289	66.8690338
564	678.1771901	66.8534927
565	679.3824914	66.8385162
566	680.587349	66.8232727
567	681.7918004	66.8082962
568	682.995816	66.793457
569	684.2025152	66.7792892
570	685.4071955	66.7648468
571	686.6123934	66.750061
572	687.8172616	66.7361602
573	689.0215167	66.7231369
574	690.2255429	66.710617
575	691.4301505	66.6983489
576	692.6343708	66.6874847
577	693.8389718	66.6780166
578	695.0435254	66.6682968
579	696.247978	66.6584396
580	697.4526015	66.6483612
581	698.6566892	66.6391754
582	699.8619064	66.6292572
583	701.0668123	66.6190338
584	702.2706098	66.6088256
585	703.4744358	66.5985412
586	704.6787567	66.5950088
587	705.8845128	66.5914306
588	707.0889358	66.5870819
589	708.294098	66.5825347
590	709.4981421	66.5775527
591	710.7026559	66.5718383
592	711.9075712	66.5659942
593	713.1127861	66.5598907
594	714.3169637	66.5618133
595	715.5220166	66.5641021
596	716.7263723	66.5580978
597	717.9296771	66.5510711
598	719.13333	66.5445022
599	720.3379743	66.5375213

600	721.5428515	66.5303802
601	722.7474299	66.5229644
602	723.9517184	66.5155563
603	725.1565117	66.5074615
604	726.3613507	66.4907226
605	727.5666696	66.4722595
606	728.7711645	66.454483
607	729.9750158	66.4364547
608	731.1791819	66.4177398
609	732.382577	66.3994369
610	733.5860979	66.3820953
611	734.7901319	66.3652191
612	735.9933313	66.3496246
613	737.1965843	66.3357849
614	738.4009837	66.3215026
615	739.6053978	66.3072662
616	740.8109397	66.2928314
617	742.0157305	66.2778244
618	743.220349	66.2639007
619	744.4235662	66.2493209
620	745.6283912	66.2330703
621	746.8324853	66.2167663
622	748.0376582	66.1984786
623	749.2426126	66.1781616
624	750.4473352	66.1585083
625	751.6514084	66.1395416
626	752.855685	66.1195068
627	754.0604852	66.1002044
628	755.2653644	66.0803833
629	756.4696919	66.0611648
630	757.6741183	66.0424957
631	758.8781232	66.0323333
632	760.0835546	66.0224685
633	761.2878822	66.0122222
634	762.4938054	66.0010375
635	763.699055	65.9892578
636	764.9033748	65.9787292
637	766.1079241	65.9667663
638	767.3126909	65.9552154
639	768.5176694	65.9427413
640	769.7224032	65.9313888
641	770.9263955	65.9119186
642	772.1305283	65.8934631
643	773.3353375	65.8768692
644	774.5403781	65.8614273
645	775.7449764	65.8480453
646	776.9500322	65.8338165
647	778.1538443	65.8198394
648	779.3587882	65.805458
649	780.5640979	65.7907104

650	781.7691634	65.7742996
651	782.973317	65.757637
652	784.1780574	65.7398529
653	785.3821757	65.720642
654	786.5866783	65.700035
655	787.7916165	65.6781997
656	788.9963871	65.6569061
657	790.201479	65.6364212
658	791.4057464	65.6158294
659	792.610176	65.5960922
660	793.8145505	65.5770187
661	795.0201729	65.5576477
662	796.2252282	65.5378417
663	797.4287845	65.5184249
664	798.6336416	65.4988708
665	799.8388988	65.4781875
666	801.0431473	65.457756
667	802.2473496	65.4381332
668	803.4524673	65.4180908
669	804.6562471	65.3984756
670	805.8600134	65.3776626
671	807.0653134	65.3552703
672	808.2699115	65.3325576
673	809.4742024	65.309143
674	810.6787705	65.2868423
675	811.8829785	65.2645416
676	813.0877956	65.2421264
677	814.2919713	65.2187042
678	815.4967396	65.1953277
679	816.7013263	65.1707839
680	817.9056436	65.1480407
681	819.1105245	65.1257934
682	820.3148772	65.1043853
683	821.520381	65.0824356
684	822.7253633	65.0601806
685	823.9297562	65.0381546
686	825.1346735	65.0145492
687	826.3382776	64.9907455
688	827.542814	64.9676055
689	828.7477154	64.9507369
690	829.9526534	64.9313812
691	831.1568995	64.9114608
692	832.3611954	64.8914642
693	833.5663814	64.8723602
694	834.7708839	64.8523788
695	835.9763775	64.8330841
696	837.1815813	64.8143234
697	838.3846156	64.7950592
698	839.5892424	64.7754058
699	840.7937364	64.749916

700	841.9974349	64.725708
701	843.2013832	64.702423
702	844.4050873	64.6790313
703	845.6089155	64.6552963
704	846.8126064	64.6315383
705	848.0171216	64.6076507
706	849.2222331	64.5837249
707	850.4270879	64.5602951
708	851.6318461	64.5362701
709	852.8361469	64.5121307
710	854.0409899	64.4873962
711	855.2454727	64.4624786
712	856.4496784	64.4368896
713	857.6548648	64.4116287
714	858.8593496	64.3863296
715	860.0635816	64.3600006
716	861.2683008	64.3336639
717	862.4732958	64.3076782
718	863.6781697	64.2817459
719	864.8813525	64.2556915
720	866.0856064	64.2306137
721	867.2895769	64.2067031
722	868.4935586	64.1832427
723	869.6971136	64.1594619
724	870.9018543	64.1366119
725	872.1068716	64.114273
726	873.3113834	64.0926055
727	874.5144744	64.0697555
728	875.7192347	64.0470581
729	876.9247798	64.0246124
730	878.1290968	64.0018005
731	879.3329379	63.9783248
732	880.5380202	63.954338
733	881.7430908	63.930767
734	882.9482664	63.9070968
735	884.153148	63.8826713
736	885.3581177	63.8582687
737	886.5614815	63.8348503
738	887.7661681	63.8115539
739	888.9710025	63.788166
740	890.1757198	63.7649154
741	891.3804846	63.7420578
742	892.5845976	63.7198677
743	893.7884434	63.6976852
744	894.9931439	63.6744575
745	896.198511	63.6525878
746	897.4032759	63.6303024
747	898.6077473	63.6072463
748	899.8121971	63.5839958
749	901.0171034	63.5605659

750	902.222474	63.5371627
751	903.4269865	63.5132942
752	904.6313199	63.4893875
753	905.8359496	63.4653701
754	907.0411147	63.4419784
755	908.2463741	63.4178657
756	909.4506524	63.394268
757	910.6552527	63.3710746
758	911.8592354	63.3478775
759	913.0643052	63.3247756
760	914.2693009	63.3018493
761	915.4742605	63.2791976
762	916.6793631	63.2569503
763	917.8842819	63.2341003
764	919.0891815	63.2109489
765	920.2935039	63.1881179
766	921.4977199	63.165039
767	922.7023078	63.1414909
768	923.9073578	63.1185798
769	925.1121934	63.0956344
770	926.3173668	63.0727005
771	927.5217748	63.0489311
772	928.7272788	63.0254936
773	929.9312176	63.0023078
774	931.1360608	62.9790229
775	932.3399782	62.9555358
776	933.5439252	62.9321823
777	934.7488253	62.9093856
778	935.9541393	62.8857498
779	937.157746	62.8612442
780	938.3619965	62.8368911
781	939.5666601	62.8138313
782	940.7706729	62.789875
783	941.9758861	62.7662429
784	943.1805881	62.74337
785	944.3859099	62.7208251
786	945.5903443	62.6975784
787	946.7943412	62.6744766
788	947.9987041	62.6518554
789	949.2037316	62.6301879
790	950.4081518	62.6079292
791	951.6134672	62.5845718
792	952.8178015	62.5617485
793	954.0213528	62.5389633
794	955.2258793	62.5151138
795	956.4305782	62.4918632
796	957.6356338	62.468132
797	958.8400113	62.4450607
798	960.0445033	62.4214782
799	961.2484208	62.3977546

800	962.4526772	62.3747482
801	963.6574918	62.3520584
802	964.8619893	62.3286361
803	966.0662723	62.305397
804	967.2693663	62.2826614
805	968.4735635	62.2592163
806	969.6792621	62.2364273
807	970.8847165	62.2126083
808	972.0896828	62.1890373
809	973.2939837	62.1663703
810	974.4978073	62.1430473
811	975.7017873	62.1190071
812	976.9061853	62.0953483
813	978.110917	62.0713996
814	979.314284	62.0478439
815	980.51929	62.0244255
816	981.7243513	62.0008773
817	982.92947	61.9771957
818	984.1340478	61.9543037
819	985.3384614	61.9304695
820	986.5429642	61.9069213
821	987.7468243	61.8844909
822	988.9504164	61.861763
823	990.1544473	61.8394546
824	991.3591046	61.8174209
825	992.5643054	61.7944755
826	993.7679321	61.7716674
827	994.9722632	61.7493209
828	996.1768429	61.726509
829	997.3814451	61.7037239
830	998.5859623	61.6816596
831	999.7896647	61.6582221
832	1000.993607	61.6359367
833	1002.197462	61.6129837
834	1003.401386	61.5893363
835	1004.606543	61.5669136
836	1005.86978	61.5437545
837	1007.074524	61.5203857
838	1008.301524	61.4970207
839	1009.50658	61.4737434
840	1010.711239	61.4497375
841	1011.915977	61.4271049
842	1013.119989	61.4033126
843	1014.324571	61.3801765
844	1015.529467	61.3580551
845	1016.750254	61.336029
846	1017.955247	61.3147277
847	1019.159142	61.2949218
848	1020.362859	61.2749938
849	1021.566852	61.2550964

850	1022.771822	61.2357673
851	1023.976773	61.2167587
852	1025.18079	61.1988525
853	1026.384789	61.1836547
854	1027.608012	61.1685295
855	1028.81166	61.1542892
856	1030.01545	61.1408271
857	1031.219311	61.126995
858	1032.423036	61.1142082
859	1033.627058	61.1024703
860	1034.831555	61.0902214
861	1036.036354	61.0774765
862	1037.240996	61.0626945
863	1038.460782	61.0449218
864	1039.664577	61.0264053
865	1040.869404	61.0064468
866	1042.074382	60.9853439
867	1043.278721	60.9633407
868	1044.483232	60.939392
869	1045.688624	60.9146308
870	1046.893221	60.8894653
871	1048.097618	60.8648185
872	1049.31869	60.8422355
873	1050.523695	60.8186569
874	1051.727737	60.7955703
875	1052.932329	60.7720031
876	1054.136869	60.7492141
877	1055.342122	60.7257919
878	1056.546825	60.7031745
879	1057.751884	60.6808891
880	1058.955872	60.6577682
881	1060.176933	60.6339836
882	1061.380499	60.6109695
883	1062.585782	60.58889
884	1063.790481	60.5650711
885	1064.995137	60.5416755
886	1066.199367	60.5175971
887	1067.403295	60.5007324
888	1068.608548	60.4850463
889	1069.81366	60.4710159
890	1071.034246	60.4591064
891	1072.246765	60.4468879
892	1073.451207	60.4327697
893	1074.655439	60.4175415
894	1075.859773	60.402729
895	1077.064753	60.3875961
896	1078.269662	60.3722419
897	1079.474549	60.3502311
898	1080.678869	60.3257522
899	1081.899696	60.2992935

900	1083.105002	60.2714729
901	1084.309259	60.2438964
902	1085.513631	60.216278
903	1086.717642	60.1898384
904	1087.922521	60.1642456
905	1089.126535	60.1393203
906	1090.330966	60.1142082
907	1091.535358	60.0891418
908	1092.758986	60.0661125
909	1093.9631	60.0431137
910	1095.167271	60.0203552
911	1096.371142	59.9972953
912	1097.574796	59.9743843
913	1098.778683	59.95158
914	1099.982485	59.9279975
915	1101.186446	59.9042549
916	1102.39002	59.8810844
917	1103.611718	59.8572158
918	1104.815541	59.8323745
919	1106.019344	59.8066711
920	1107.223162	59.7809829
921	1108.427053	59.7550544
922	1109.630825	59.7298774
923	1110.834615	59.7043952
924	1112.038289	59.679779
925	1113.243243	59.65559
926	1114.463927	59.6315536
927	1115.66768	59.6086044
928	1116.871345	59.585865
929	1118.075166	59.5631103
930	1119.278972	59.5409545
931	1120.483274	59.5195541
932	1121.688381	59.4976005
933	1122.892641	59.475605
934	1124.098133	59.4530105
935	1125.317537	59.4292488
936	1126.522297	59.4058456
937	1127.726473	59.3817024
938	1128.930506	59.3574104
939	1130.135579	59.333641
940	1131.339906	59.3102226
941	1132.545187	59.286621
942	1133.749892	59.2635726
943	1134.954434	59.24057
944	1136.175195	59.2180709
945	1137.379996	59.1955947
946	1138.585422	59.1737251
947	1139.790059	59.1545219
948	1140.995444	59.1347427
949	1142.199154	59.114624

950	1143.403013	59.0938606
951	1144.607267	59.0730056
952	1145.81186	59.0522994
953	1147.032933	59.0319633
954	1148.2366	59.0115852
955	1149.44026	58.9915657
956	1150.643904	58.9705314
957	1151.847585	58.9478645
958	1153.051468	58.9257507
959	1154.255195	58.9042243
960	1155.459202	58.8825874
961	1156.66381	58.8602409
962	1157.884785	58.8374099
963	1159.088991	58.8143386
964	1160.294351	58.791069
965	1161.497578	58.7678833
966	1162.701413	58.7446517
967	1163.906508	58.721363
968	1165.111441	58.6980361
969	1166.316545	58.6749076
970	1167.521023	58.6523323
971	1168.741198	58.6299934
972	1169.945881	58.6076164
973	1171.150631	58.5858535
974	1172.355195	58.5645141
975	1173.559736	58.5432853
976	1174.763576	58.5231552
977	1175.967646	58.5037918
978	1177.172493	58.4854049
979	1178.377008	58.4677925
980	1179.598266	58.4496345
981	1180.803183	58.4325027
982	1182.007961	58.4156799
983	1183.212255	58.3991699
984	1184.416914	58.3823242
985	1185.621622	58.3658256
986	1186.825537	58.3490791
987	1188.030455	58.3313217
988	1189.234459	58.3127517
989	1190.455963	58.2941474
990	1191.661326	58.2753601
991	1192.86566	58.2569961
992	1194.070545	58.238964
993	1195.274688	58.2210922
994	1196.479275	58.2037658
995	1197.683186	58.187397
996	1198.887182	58.1717453
997	1200.091635	58.1582527
998	1201.312995	58.1479263
999	1202.517137	58.1395874

1000	1203.721668	58.1339645
1001	1204.926176	58.1292724
1002	1206.131284	58.1270866
1003	1207.397248	58.1260185
1004	1208.601736	58.1269035
1005	1209.805739	58.126091
1006	1211.010454	58.125267
1007	1212.23277	58.1225051
1008	1213.4369	58.1174011
1009	1214.640613	58.1101608
1010	1215.844225	58.1015396
1011	1217.048967	58.0924682
1012	1218.25409	58.0808906
1013	1219.458792	58.0684204
1014	1220.663081	58.0545425
1015	1221.86653	58.041706
1016	1223.088586	58.0288581
1017	1224.293019	58.0160217
1018	1225.497316	58.0035095
1019	1226.701987	57.9911003
1020	1227.906042	57.9794807
1021	1229.110035	57.9672317
1022	1230.31491	57.955986
1023	1231.519687	57.9446411
1024	1232.723932	57.933361
1025	1233.945342	57.922882
1026	1235.149629	57.9132919
1027	1236.354166	57.9036788
1028	1237.55921	57.8954467
1029	1238.764084	57.8872489
1030	1239.968909	57.8783569
1031	1241.172029	57.8704414
1032	1242.376266	57.8623352
1033	1243.58145	57.8548812
1034	1244.801859	57.848133
1035	1246.00721	57.8418273
1036	1247.211746	57.8348007
1037	1248.416157	57.8279533
1038	1249.620925	57.8204269
1039	1250.825752	57.8135986
1040	1252.03148	57.8066902
1041	1253.236576	57.7999496
1042	1254.440586	57.7928657
1043	1255.661717	57.7854843
1044	1256.866536	57.7778091
1045	1258.071583	57.769329
1046	1259.276546	57.7605895
1047	1260.480415	57.752552
1048	1261.683721	57.7442855
1049	1262.888286	57.7361679

1050	1264.09404	57.7292556
1051	1265.298238	57.7207641
1052	1266.517954	57.7138557
1053	1267.722209	57.7070312
1054	1268.926792	57.7010688
1055	1270.131501	57.6960449
1056	1271.336806	57.6913757
1057	1272.541574	57.6868209
1058	1273.74555	57.6827011
1059	1274.949309	57.6776962
1060	1276.153596	57.6715812
1061	1277.376847	57.6667671
1062	1278.581202	57.660614
1063	1279.785585	57.6550979
1064	1280.989386	57.6479682
1065	1282.193636	57.6409072
1066	1283.398834	57.6337089
1067	1284.604008	57.6273994
1068	1285.816825	57.6205558
1069	1287.021446	57.6159133
1070	1288.233705	57.6126708
1071	1289.438322	57.6100959
1072	1290.643314	57.6091613
1073	1291.848013	57.6071662
1074	1293.052159	57.6067886
1075	1294.256188	57.6065177
1076	1295.460935	57.6068115
1077	1296.666035	57.6062202
1078	1297.870285	57.6052932
1079	1299.089856	57.602539
1080	1300.293658	57.5987548
1081	1301.49703	57.5949325
1082	1302.701637	57.589302
1083	1303.906249	57.5838699
1084	1305.110881	57.5774307
1085	1306.315598	57.5700683
1086	1307.520238	57.5623283
1087	1308.72381	57.5545463
1088	1309.945967	57.547676
1089	1311.150731	57.5408935
1090	1312.356047	57.5337028
1091	1313.563371	57.5255317
1092	1314.767107	57.5171585
1093	1315.97262	57.5091629
1094	1317.17857	57.500843
1095	1318.383951	57.4930305
1096	1319.588974	57.4837532
1097	1320.808741	57.474205
1098	1322.012262	57.4640083
1099	1323.216327	57.4535217

1100	1324.421256	57.4425582
1101	1325.624902	57.4306259
1102	1326.83001	57.4188308
1103	1328.034291	57.406414
1104	1329.238456	57.393547
1105	1330.442184	57.3792304
1106	1331.6645	57.3645439
1107	1332.868327	57.3494682
1108	1334.072577	57.3338737
1109	1335.278772	57.318901
1110	1336.483416	57.3044548
1111	1337.688706	57.2906074
1112	1338.893224	57.2772712
1113	1340.097557	57.263813
1114	1341.301706	57.2514228
1115	1342.523078	57.2404174
1116	1343.726585	57.2293586
1117	1344.93147	57.2191848
1118	1346.135353	57.2092475
1119	1347.338922	57.1992721
1120	1348.543332	57.1895866
1121	1349.74811	57.1810379
1122	1350.952501	57.1719131
1123	1352.156794	57.1631469
1124	1353.377883	57.1535453
1125	1354.582594	57.1436576
1126	1355.787265	57.1352615
1127	1356.991991	57.1251716
1128	1358.19698	57.1226272
1129	1359.401601	57.1193733
1130	1360.605398	57.1153335
1131	1361.809938	57.1103439
1132	1363.014436	57.106163
1133	1364.23639	57.1023445
1134	1365.440969	57.1005783
1135	1366.644923	57.0992546
1136	1367.849097	57.0984878
1137	1369.052962	57.0988273
1138	1370.258301	57.0921211
1139	1371.463255	57.0845222
1140	1372.668246	57.0767631
1141	1373.873609	57.0676078
1142	1375.092923	57.0564193
1143	1376.297916	57.0439949
1144	1377.502296	57.0299453
1145	1378.706936	57.0147323
1146	1379.911784	57.0001029
1147	1381.115665	56.9857559
1148	1382.320739	56.971302
1149	1383.52553	56.9578781

1150	1384.73075	56.9455451
1151	1385.951435	56.9331436
1152	1387.156549	56.9203567
1153	1388.361017	56.9065132
1154	1389.566732	56.8917465
1155	1390.770988	56.8772087
1156	1391.975602	56.8611335
1157	1393.180507	56.8443984
1158	1394.38421	56.8275642
1159	1395.5887	56.8103828
1160	1396.811279	56.7922782
1161	1398.015811	56.7760925
1162	1399.220022	56.7620353
1163	1400.42444	56.7502555
1164	1401.628003	56.7404174
1165	1402.832648	56.7324829
1166	1404.037465	56.7253532
1167	1405.24227	56.7209243
1168	1406.447081	56.7170104
1169	1407.690519	56.7139968
1170	1408.896278	56.7120857
1171	1410.101054	56.7099685
1172	1411.306036	56.7071952
1173	1412.510725	56.704113
1174	1413.714517	56.6994132
1175	1414.918119	56.6931686
1176	1416.122312	56.6857681
1177	1417.326947	56.6755905
1178	1418.548946	56.6653861
1179	1419.754058	56.6542282
1180	1420.958814	56.6426506
1181	1422.165774	56.6312751
1182	1423.370004	56.6207885
1183	1424.575087	56.6122856
1184	1425.780405	56.6055068
1185	1426.986476	56.5989456
1186	1428.190842	56.5935058
1187	1429.409777	56.5891036
1188	1430.613907	56.5849723
1189	1431.818142	56.5820732
1190	1433.022856	56.5794563
1191	1434.226424	56.5774841
1192	1435.430074	56.5749931
1193	1436.635581	56.5704345
1194	1437.840316	56.5659255
1195	1439.04531	56.5608825
1196	1440.266073	56.555931
1197	1441.47023	56.5507431
1198	1442.674698	56.5448532
1199	1443.879699	56.5382003

1200	1445.08465	56.5311279
1201	1446.290015	56.5232048
1202	1447.49501	56.5163383
1203	1448.699295	56.5106582
1204	1449.904071	56.505516
1205	1451.125823	56.5019454
1206	1452.329011	56.4990005
1207	1453.532912	56.4969367
1208	1454.736907	56.4950637
1209	1455.940544	56.4931755
1210	1457.144304	56.4909477
1211	1458.348079	56.4897994
1212	1459.55199	56.487873
1213	1460.75652	56.4853935
1214	1461.977842	56.4811019
1215	1463.181547	56.4758491
1216	1464.386767	56.4701423
1217	1465.591447	56.4637222
1218	1466.795802	56.4579162
1219	1467.999487	56.4533805
1220	1469.203434	56.4523735
1221	1470.407395	56.4528007
1222	1471.612642	56.4553909
1223	1472.834015	56.4579048
1224	1474.038724	56.4612197
1225	1475.241873	56.4641952
1226	1476.446463	56.4667587
1227	1477.651892	56.4696731
1228	1478.856186	56.4723281
1229	1480.060556	56.4756774
1230	1481.264387	56.476612
1231	1482.468829	56.4757194
1232	1483.690009	56.4736213
1233	1484.895483	56.4713554
1234	1486.100927	56.4686393
1235	1487.30505	56.4652519
1236	1488.509313	56.4615707
1237	1489.713712	56.457386
1238	1490.917749	56.4532279
1239	1492.121082	56.4468917
1240	1493.325007	56.4405326
1241	1494.547883	56.4345893
1242	1495.751753	56.4280929
1243	1496.955544	56.4217948
1244	1498.160499	56.4155883
1245	1499.365637	56.4096908
1246	1500.571192	56.4041137
1247	1501.775811	56.3990249
1248	1502.979416	56.3930473
1249	1504.183609	56.3868713

1250	1505.406201	56.3795547
1251	1506.61152	56.3708419
1252	1507.81628	56.3615226
1253	1509.020576	56.35083
1254	1510.225458	56.3396682
1255	1511.430639	56.3287277
1256	1512.635355	56.3166122
1257	1513.839432	56.3039016
1258	1515.042556	56.2914199
1259	1516.265527	56.2799987
1260	1517.470311	56.2694282
1261	1518.67569	56.2594718
1262	1519.880689	56.2489204
1263	1521.084094	56.2388916
1264	1522.288107	56.2292861
1265	1523.492244	56.2203865
1266	1524.696354	56.2141189
1267	1525.90064	56.2098503
1268	1527.122378	56.2078971
1269	1528.326808	56.205635
1270	1529.531145	56.2031784
1271	1530.734454	56.2006416
1272	1531.939775	56.198944
1273	1533.144782	56.1972846
1274	1534.350008	56.1958541
1275	1535.55388	56.1942138
1276	1536.758866	56.190876
1277	1537.98038	56.1862297
1278	1539.183715	56.1798324
1279	1540.387977	56.1727523
1280	1541.592197	56.1653976
1281	1542.796223	56.1577873
1282	1544.000985	56.1495285
1283	1545.205775	56.1417121
1284	1546.410671	56.1336364
1285	1547.61389	56.1258659
1286	1548.834874	56.1178779
1287	1550.038507	56.1092643
1288	1551.2435	56.1008224
1289	1552.44814	56.0934753
1290	1553.653605	56.0867996
1291	1554.857781	56.0805473
1292	1556.062303	56.0741462
1293	1557.26727	56.0688591
1294	1558.472914	56.0646667
1295	1559.692416	56.0612068
1296	1560.896113	56.0582885
1297	1562.100255	56.0563735
1298	1563.304628	56.0546684
1299	1564.508775	56.0517158

1300	1565.714511	56.0492553
1301	1566.91911	56.0462036
1302	1568.123094	56.0441513
1303	1569.326712	56.0409126
1304	1570.548961	56.0368766
1305	1571.753858	56.0323905
1306	1572.958842	56.027687
1307	1574.163811	56.0223464
1308	1575.368456	56.0177917
1309	1576.573373	56.0138473
1310	1577.777973	56.0091781
1311	1578.983567	56.0040702
1312	1580.188244	55.9984664
1313	1581.407783	55.9920578
1314	1582.611635	55.9851913
1315	1583.816082	55.9765625
1316	1585.021106	55.9671516
1317	1586.225694	55.9563713
1318	1587.42986	55.9433059
1319	1588.633522	55.9303894
1320	1589.837298	55.9175338
1321	1591.041791	55.9052047
1322	1592.263902	55.8932075
1323	1593.468521	55.8809318
1324	1594.671926	55.8684005
1325	1595.875287	55.8560791
1326	1597.079976	55.844429
1327	1598.284123	55.833992
1328	1599.488784	55.8240242
1329	1600.693044	55.8135795
1330	1601.897081	55.8036918
1331	1603.118793	55.7949333
1332	1604.323624	55.786621
1333	1605.529003	55.7786369
1334	1606.733856	55.7713012
1335	1607.994731	55.7655715
1336	1609.198458	55.7624626
1337	1610.403436	55.760334
1338	1611.608666	55.7600517
1339	1612.813164	55.7609252
1340	1614.033747	55.7621231
1341	1615.238262	55.7632522
1342	1616.442677	55.763935
1343	1617.646204	55.7646484
1344	1618.851029	55.7646598
1345	1620.055551	55.764801
1346	1621.260444	55.7630844
1347	1622.465546	55.7601242
1348	1623.670116	55.7553939
1349	1624.890715	55.7503738

1350	1626.096354	55.7447013
1351	1627.300837	55.7378921
1352	1628.504386	55.730091
1353	1629.708076	55.7231521
1354	1630.912989	55.7171401
1355	1632.118056	55.7094726
1356	1633.323346	55.7020034
1357	1634.527717	55.6938667
1358	1635.747735	55.6870841
1359	1636.952352	55.679779
1360	1638.157366	55.6721878
1361	1639.362553	55.6643142
1362	1640.56737	55.6566047
1363	1641.771613	55.6482963
1364	1642.975992	55.6391601
1365	1644.181415	55.6299209
1366	1645.385632	55.6191177
1367	1646.606086	55.6087226
1368	1647.810168	55.596508
1369	1649.014515	55.5845108
1370	1650.218568	55.5717048
1371	1651.423222	55.5603866
1372	1652.628056	55.5495185
1373	1653.832999	55.5389709
1374	1655.037	55.5282974
1375	1656.241043	55.5188903
1376	1657.462824	55.5169143
1377	1658.667111	55.5152397
1378	1659.871312	55.5140914
1379	1661.075369	55.5119705
1380	1662.281065	55.5099487
1381	1663.485524	55.508171
1382	1664.690362	55.5060501
1383	1665.895515	55.5039787
1384	1667.100654	55.5026969
1385	1668.320692	55.5017395
1386	1669.524658	55.4943847
1387	1670.728929	55.4877738
1388	1671.933871	55.481781
1389	1673.138773	55.4775886
1390	1674.342671	55.4738388
1391	1675.547819	55.4703292
1392	1676.75271	55.4661254
1393	1677.957761	55.4622268
1394	1679.179812	55.4585456
1395	1680.383984	55.4538154
1396	1681.588209	55.4499626
1397	1682.792393	55.4500007
1398	1683.997258	55.4486885
1399	1685.201731	55.4458007

1400	1686.40742	55.4423942
1401	1687.612272	55.4368591
1402	1688.816159	55.430725
1403	1690.038147	55.4240837
1404	1691.243071	55.4148254
1405	1692.447578	55.4039535
1406	1693.652824	55.3915405
1407	1694.856706	55.3737678
1408	1696.0619	55.3570671
1409	1697.266341	55.3412399
1410	1698.470735	55.3271064
1411	1699.675585	55.3147659
1412	1700.897234	55.3048629
1413	1702.100993	55.2957267
1414	1703.305916	55.2892265
1415	1704.51142	55.283638
1416	1705.716675	55.2787094
1417	1706.920583	55.2740669
1418	1708.125163	55.2697257
1419	1709.329766	55.2656936
1420	1710.534301	55.261425
1421	1711.756363	55.2569465
1422	1712.960802	55.250885
1423	1714.164948	55.2439155
1424	1715.370228	55.2349929
1425	1716.574148	55.2255973
1426	1717.77884	55.2147445
1427	1718.983557	55.2034912
1428	1720.188521	55.1910514
1429	1721.392438	55.176342
1430	1722.613655	55.1604804
1431	1723.817054	55.1438102
1432	1725.021598	55.1281242
1433	1726.225795	55.1143417
1434	1727.430381	55.1033439
1435	1728.63418	55.0951728
1436	1729.839668	55.089302
1437	1731.044022	55.0844001
1438	1732.249035	55.080101
1439	1733.470855	55.0796012
1440	1734.675275	55.0783309
1441	1735.879756	55.0776557
1442	1737.084375	55.0768432
1443	1738.289188	55.0741386
1444	1739.494025	55.0700302
1445	1740.69872	55.0658378
1446	1741.903247	55.0612373
1447	1743.107705	55.0570297
1448	1744.328141	55.0538711
1449	1745.533195	55.0482101

1450	1746.739006	55.042572
1451	1747.943045	55.0365219
1452	1749.147252	55.0300216
1453	1750.351308	55.0234565
1454	1751.556322	55.0162239
1455	1752.76103	55.007801
1456	1753.965335	54.9982833
1457	1755.186083	54.9877128
1458	1756.390941	54.9759101
1459	1757.595946	54.9643669
1460	1758.800493	54.9533195
1461	1760.004301	54.9415512
1462	1761.208323	54.9298286
1463	1762.411949	54.9188346
1464	1763.615723	54.9083023
1465	1764.819538	54.8977127
1466	1766.042877	54.8872451
1467	1767.247683	54.8782348
1468	1768.451493	54.869728
1469	1769.655528	54.8619766
1470	1770.859615	54.8543319
1471	1772.064263	54.848278
1472	1773.268613	54.8427925
1473	1774.472334	54.8375015
1474	1775.676447	54.8327407
1475	1776.899203	54.8293571
1476	1778.104786	54.827156
1477	1779.30946	54.823986
1478	1780.513588	54.8210144
1479	1781.717823	54.8168525
1480	1782.921613	54.8122024
1481	1784.127245	54.8061523
1482	1785.332178	54.7985954
1483	1786.536874	54.7902832
1484	1787.757386	54.7806625
1485	1788.961203	54.7693595
1486	1790.166	54.7566642
1487	1791.370434	54.7438278
1488	1792.573985	54.7301139
1489	1793.778441	54.7166671
1490	1794.983347	54.7044754
1491	1796.187598	54.6916694
1492	1797.392024	54.6797256
1493	1798.613147	54.6689872
1494	1799.817044	54.6579818
1495	1801.020385	54.6473503
1496	1802.225059	54.6366577
1497	1803.429991	54.625679
1498	1804.634808	54.6155281
1499	1805.839366	54.6049919

1500	1807.044461	54.5940132
1501	1808.249608	54.5842094
1502	1809.469581	54.5752868
1503	1810.673031	54.5655632
1504	1811.878101	54.5565414
1505	1813.082504	54.5478935
1506	1814.286726	54.5391235
1507	1815.490972	54.5311889
1508	1816.696039	54.52182
1509	1817.900841	54.5147514
1510	1819.105809	54.507061
1511	1820.326623	54.4983825
1512	1821.530915	54.4887924
1513	1822.735077	54.4785881
1514	1823.939207	54.4685173
1515	1825.143725	54.4574394
1516	1826.348774	54.4470443
1517	1827.553336	54.4356689
1518	1828.757687	54.4259757
1519	1829.962542	54.4163208
1520	1831.183471	54.4083061
1521	1832.388418	54.4010696
1522	1833.593354	54.3937377
1523	1834.796678	54.3857841
1524	1836.00066	54.3775711
1525	1837.205475	54.3699111
1526	1838.410577	54.3621292
1527	1839.615593	54.3536186
1528	1840.819509	54.3443717
1529	1842.040334	54.3325271
1530	1843.244642	54.3187942
1531	1844.450083	54.3052368
1532	1845.655246	54.2910957
1533	1846.859362	54.27816
1534	1848.063618	54.2651138
1535	1849.267466	54.2519035
1536	1850.471689	54.2385978
1537	1851.676729	54.2266426
1538	1852.897339	54.2146415
1539	1854.100772	54.203247
1540	1855.305543	54.1925239
1541	1856.50998	54.1817932
1542	1857.714232	54.1720657
1543	1858.919841	54.1620979
1544	1860.12362	54.1522636
1545	1861.326904	54.1428222
1546	1862.531172	54.1330184
1547	1863.754587	54.1227531
1548	1864.959027	54.1126708
1549	1866.16305	54.1017341

1550	1867.367614	54.0904769
1551	1868.572446	54.0783157
1552	1869.776322	54.066658
1553	1870.981474	54.0546073
1554	1872.186083	54.041851
1555	1873.390546	54.0276718
1556	1874.61156	54.0140876
1557	1875.815772	54.001213
1558	1877.019974	53.9876403
1559	1878.224244	53.9754943
1560	1879.428944	53.9629745
1561	1880.633781	53.9504776
1562	1881.837236	53.9372329
1563	1883.041108	53.9238662
1564	1884.246051	53.9115676
1565	1885.468802	53.9008102
1566	1886.673439	53.8898429
1567	1887.877629	53.8785171
1568	1889.081713	53.8667488
1569	1890.287324	53.8542785
1570	1891.491164	53.8425216
1571	1892.695753	53.8312683
1572	1893.900948	53.8199844
1573	1895.104726	53.8082962
1574	1896.326402	53.7962532
1575	1897.530525	53.7841415
1576	1898.734097	53.7719421
1577	1899.938474	53.7597274
1578	1901.149739	53.7476501
1579	1902.353737	53.7353134
1580	1903.558851	53.7231445
1581	1904.764229	53.7099113
1582	1905.969654	53.6967468
1583	1907.189723	53.6844062
1584	1908.393474	53.671894
1585	1909.596867	53.6586418
1586	1910.801327	53.6455688
1587	1912.005521	53.6320686
1588	1913.210196	53.6184196
1589	1914.414253	53.605236
1590	1915.618437	53.5921325
1591	1916.822709	53.5788154
1592	1918.044757	53.5655632
1593	1919.249653	53.5523262
1594	1920.453471	53.5387763
1595	1921.657062	53.5262336
1596	1922.861998	53.5133743
1597	1924.066478	53.5006294
1598	1925.271181	53.4880142
1599	1926.476191	53.4750785

1600	1927.681085	53.4624023
1601	1928.900824	53.4505424
1602	1930.105947	53.4387931
1603	1931.311474	53.4267158
1604	1932.516067	53.4146842
1605	1933.720047	53.4015922
1606	1934.923615	53.3887825
1607	1936.127416	53.3760299
1608	1937.331279	53.3633499
1609	1938.535108	53.350563
1610	1939.758007	53.3367309
1611	1940.962524	53.3229179
1612	1942.166107	53.3089485
1613	1943.371019	53.2947578
1614	1944.576813	53.2813835
1615	1945.78201	53.2679939
1616	1946.986716	53.254692
1617	1948.191254	53.2408561
1618	1949.395696	53.2267761
1619	1950.616766	53.2135238
1620	1951.82121	53.2000846
1621	1953.02752	53.187416
1622	1954.231524	53.1739997
1623	1955.435249	53.1603813
1624	1956.639725	53.1464004
1625	1957.844373	53.1326904
1626	1959.049543	53.118164
1627	1960.253476	53.1044692
1628	1961.474598	53.0915298
1629	1962.67881	53.0776138
1630	1963.882651	53.0644302
1631	1965.087271	53.0500717
1632	1966.292264	53.0359802
1633	1967.496063	53.0225296
1634	1968.69939	53.0093269
1635	1969.903394	52.9952774
1636	1971.10844	52.9807243
1637	1972.328893	52.9658889
1638	1973.532993	52.9503784
1639	1974.736914	52.9349594
1640	1975.940897	52.9188919
1641	1977.144774	52.9036254
1642	1978.348553	52.8886642
1643	1979.553627	52.8742179
1644	1980.757243	52.8586654
1645	1981.961175	52.8430061
1646	1983.18459	52.8283081
1647	1984.389353	52.8135261
1648	1985.59392	52.7988662
1649	1986.797215	52.7843399

1650	1988.001392	52.7697944
1651	1989.205766	52.7551155
1652	1990.410582	52.7406997
1653	1991.61476	52.7255325
1654	1992.819943	52.7112846
1655	1994.040299	52.6972846
1656	1995.244204	52.683403
1657	1996.448016	52.6692276
1658	1997.652385	52.65522
1659	1998.857682	52.6421012
1660	2000.062763	52.6287918
1661	2001.267629	52.6152038
1662	2002.471779	52.6010055
1663	2003.676848	52.5866203
1664	2004.904043	52.5729484
1665	2006.108216	52.5584182
1666	2007.31316	52.5437545
1667	2008.518299	52.5294914
1668	2009.722791	52.5149536
1669	2010.932639	52.4996719
1670	2012.137132	52.4844245
1671	2013.341326	52.4691162
1672	2014.545145	52.4543342
1673	2015.767416	52.4403686
1674	2016.972024	52.4254684
1675	2018.175963	52.4118652
1676	2019.380886	52.3982658
1677	2020.586062	52.3840103
1678	2021.790297	52.3699226
1679	2022.994669	52.3555831
1680	2024.199462	52.3420219
1681	2025.404245	52.3281059
1682	2026.624672	52.3139648
1683	2027.829468	52.2984924
1684	2029.033749	52.2829475
1685	2030.23857	52.2670669
1686	2031.443924	52.25111
1687	2032.648635	52.2354965
1688	2033.853577	52.2202148
1689	2035.058071	52.2051773
1690	2036.261753	52.1887435
1691	2037.483322	52.1725654
1692	2038.688333	52.1560134
1693	2039.893953	52.14085
1694	2041.097312	52.1255378
1695	2042.300378	52.1101875
1696	2043.504391	52.0947151
1697	2044.708928	52.078907
1698	2045.91349	52.062828
1699	2047.119301	52.0473442

1700	2048.338628	52.0327453
1701	2049.543142	52.0179748
1702	2050.747584	52.0038986
1703	2051.952363	51.9890022
1704	2053.15619	51.9741134
1705	2054.360822	51.959095
1706	2055.564901	51.9435539
1707	2056.768384	51.9293022
1708	2057.973872	51.9152107
1709	2059.194417	51.8993644
1710	2060.398643	51.8835563
1711	2061.601739	51.867424
1712	2062.805445	51.8511161
1713	2064.009083	51.8344001
1714	2065.212916	51.8182792
1715	2066.416659	51.802082
1716	2067.620372	51.7861824
1717	2068.824293	51.7696037
1718	2070.046398	51.7533149
1719	2071.251128	51.7385749
1720	2072.455315	51.7237548
1721	2073.660526	51.7100296
1722	2074.865399	51.6949539
1723	2076.069034	51.6805915
1724	2077.274104	51.6653861
1725	2078.478358	51.6506767
1726	2079.682181	51.6356735
1727	2080.904023	51.6198844
1728	2082.10875	51.6030464
1729	2083.312905	51.5854606
1730	2084.51759	51.5681228
1731	2085.722225	51.5493392
1732	2086.926907	51.53125
1733	2088.131877	51.5128898
1734	2089.336977	51.4945144
1735	2090.541855	51.4751396
1736	2091.761593	51.4562683
1737	2092.965501	51.437561
1738	2094.170323	51.4196472
1739	2095.373655	51.4022521
1740	2096.582124	51.3846931
1741	2097.786259	51.3674392
1742	2098.991568	51.3503608
1743	2100.197594	51.3330574
1744	2101.403019	51.31501
1745	2102.622199	51.2977523
1746	2103.826859	51.2803649
1747	2105.031494	51.2628479
1748	2106.23653	51.2453498
1749	2107.441422	51.2270202

1750	2108.645979	51.2086105
1751	2109.850866	51.1909255
1752	2111.055623	51.1723556
1753	2112.260626	51.1539688
1754	2113.480557	51.1363105
1755	2114.685051	51.1185836
1756	2115.889343	51.1002807
1757	2117.093822	51.0825347
1758	2118.29831	51.064289
1759	2119.502578	51.0462455
1760	2120.70835	51.0276908
1761	2121.912902	51.0085868
1762	2123.116591	50.9903259
1763	2124.338947	50.9727249
1764	2125.543469	50.9566688
1765	2126.74911	50.94104
1766	2127.954315	50.9264869
1767	2129.158526	50.9117965
1768	2130.362728	50.8965263
1769	2131.568057	50.8806381
1770	2132.77381	50.8652305
1771	2133.978727	50.8493118
1772	2135.198579	50.8332786
1773	2136.402301	50.8162689
1774	2137.606504	50.798069
1775	2138.81136	50.7784919
1776	2140.016186	50.7582321
1777	2141.220613	50.7369384
1778	2142.424799	50.7170028
1779	2143.629811	50.6977005
1780	2144.834025	50.6782989
1781	2146.055762	50.6596832
1782	2147.260375	50.6409568
1783	2148.464153	50.6221389
1784	2149.668533	50.6035995
1785	2150.873274	50.5847854
1786	2152.077697	50.5658378
1787	2153.282493	50.5477676
1788	2154.48716	50.5290832
1789	2155.691474	50.5092163
1790	2156.91259	50.4888877
1791	2158.117683	50.4681625
1792	2159.322231	50.4473381
1793	2160.526016	50.425045
1794	2161.7296	50.4020614
1795	2162.933914	50.3803863
1796	2164.137903	50.3590736
1797	2165.342427	50.337387
1798	2166.547129	50.3158836
1799	2167.768332	50.2959976

1800	2168.97265	50.2768249
1801	2170.177527	50.2573394
1802	2171.382341	50.2382469
1803	2172.587559	50.2202987
1804	2173.792245	50.2027816
1805	2174.996574	50.1847915
1806	2176.199959	50.1661453
1807	2177.403247	50.1476135
1808	2178.626107	50.1281814
1809	2179.830439	50.1083183
1810	2181.034517	50.0883102
1811	2182.238813	50.0685195
1812	2183.442757	50.0480422
1813	2184.647224	50.0283012
1814	2185.852212	50.0083198
1815	2187.056555	49.9883308
1816	2188.260494	49.9684066
1817	2189.481536	49.9488601
1818	2190.686012	49.9301338
1819	2191.890766	49.9110641
1820	2193.09559	49.8923606
1821	2194.300389	49.8736877
1822	2195.504722	49.8546752
1823	2196.70883	49.8353538
1824	2197.913663	49.8162269
1825	2199.117722	49.7967338
1826	2200.338335	49.7773628
1827	2201.542397	49.7581825
1828	2202.746715	49.738903
1829	2203.950481	49.718811
1830	2205.155513	49.6983222
1831	2206.360977	49.6775741
1832	2207.617923	49.6575622
1833	2208.839196	49.6370048
1834	2210.043504	49.6161422
1835	2211.313129	49.5955009
1836	2212.516477	49.5748443
1837	2213.719685	49.5533256
1838	2214.92434	49.5317764
1839	2216.129562	49.5118904
1840	2217.334847	49.4910659
1841	2218.539717	49.4702224
1842	2219.744726	49.4498748
1843	2220.94889	49.4294776
1844	2222.153085	49.4096488
1845	2223.357424	49.3900451
1846	2224.562313	49.3700752
1847	2225.766968	49.350624
1848	2226.97137	49.3311729
1849	2228.176447	49.3105735

1850	2229.38016	49.2900466
1851	2230.58412	49.2700424
1852	2231.789048	49.2496719
1853	2232.994034	49.2299461
1854	2234.198375	49.2095985
1855	2235.402698	49.188858
1856	2236.637865	49.1686401
1857	2237.842986	49.1482772
1858	2239.047596	49.1269493
1859	2240.251892	49.1064071
1860	2241.456164	49.0863723
1861	2242.661242	49.0660018
1862	2243.865591	49.0453872
1863	2245.0706	49.0248298
1864	2246.27597	49.0045394
1865	2247.480534	48.9847145
1866	2248.683615	48.9627304
1867	2249.8882	48.9392852
1868	2251.0936	48.9162902
1869	2252.298543	48.8924942
1870	2253.502981	48.8683624
1871	2254.70669	48.8437042
1872	2255.910738	48.8192672
1873	2257.115842	48.7938308
1874	2258.320347	48.7689285
1875	2259.525659	48.7433013
1876	2260.729384	48.7193679
1877	2261.932792	48.6972503
1878	2263.136722	48.6752853
1879	2264.341331	48.6537094
1880	2265.545939	48.6328048
1881	2266.750924	48.6128082
1882	2267.955214	48.5927619
1883	2269.158998	48.5736808
1884	2270.362957	48.5533485
1885	2271.568074	48.5336647
1886	2272.772766	48.5140647
1887	2273.976981	48.4942092
1888	2275.181012	48.4744338
1889	2276.385021	48.4543533
1890	2277.589473	48.4344139
1891	2278.796045	48.4142379
1892	2280.000871	48.3939819
1893	2281.205089	48.3730163
1894	2282.409244	48.3530349
1895	2283.613845	48.3328018
1896	2284.818504	48.312828
1897	2286.023735	48.2923698
1898	2287.22782	48.2718734
1899	2288.43233	48.2512283

1900	2289.636704	48.2303619
1901	2290.841801	48.2094345
1902	2292.047245	48.1892356
1903	2293.251996	48.1693763
1904	2294.455465	48.1487236
1905	2295.661498	48.1277313
1906	2296.866472	48.1072769
1907	2298.071363	48.0873031
1908	2299.275255	48.067192
1909	2300.479561	48.0478935
1910	2301.683786	48.0281753
1911	2302.887741	48.008274
1912	2304.091819	47.9878273
1913	2305.296456	47.9676856
1914	2306.501311	47.9475021
1915	2307.705756	47.9279289
1916	2308.910796	47.9077835
1917	2310.115112	47.8876533
1918	2311.320196	47.8671035
1919	2312.525474	47.8471527
1920	2313.7303	47.8265533
1921	2314.934242	47.8060531
1922	2316.138431	47.7856559
1923	2317.343157	47.7653427
1924	2318.547751	47.7450942
1925	2319.753325	47.7253799
1926	2320.958526	47.7053909
1927	2322.163992	47.6848106
1928	2323.368803	47.6653976
1929	2324.573093	47.6456146
1930	2325.777462	47.6262969
1931	2326.982398	47.6069831
1932	2328.186999	47.5879516
1933	2329.391553	47.5688552
1934	2330.596593	47.5491371
1935	2331.801433	47.529087
1936	2333.006836	47.509407
1937	2334.211777	47.4906082
1938	2335.416231	47.4706611
1939	2336.621955	47.4502563
1940	2337.825515	47.4306488
1941	2339.030959	47.4113388
1942	2340.235946	47.3911628
1943	2341.440858	47.3704071
1944	2342.645183	47.3507575
1945	2343.849801	47.3309555
1946	2345.054343	47.3114814
1947	2346.259302	47.2909164
1948	2347.464039	47.2712173
1949	2348.66756	47.2509994

1950	2349.872371	47.2310829
1951	2351.077289	47.2111816
1952	2352.28254	47.1917037
1953	2353.486434	47.1733894
1954	2354.691886	47.1546249
1955	2355.895658	47.1362342
1956	2357.100314	47.1167068
1957	2358.305354	47.0981674
1958	2359.510901	47.0799713
1959	2360.714525	47.0616836
1960	2361.919269	47.0424652
1961	2363.123525	47.0232887
1962	2364.328398	47.0037307
1963	2365.532652	46.9838638
1964	2366.73762	46.9642906
1965	2367.942149	46.9441413
1966	2369.146407	46.9249496
1967	2370.350082	46.9047622
1968	2371.554543	46.8837051
1969	2372.758346	46.8636703
1970	2373.962346	46.84375
1971	2375.166199	46.8238754
1972	2376.370259	46.8037452
1973	2377.57459	46.7834854
1974	2378.779811	46.7632217
1975	2379.985118	46.74337
1976	2381.18986	46.7231674
1977	2382.393886	46.7036056
1978	2383.597895	46.6850929
1979	2384.802442	46.6662979
1980	2386.0069	46.6483764
1981	2387.212511	46.6293029
1982	2388.416447	46.6113967
1983	2389.621263	46.5930709
1984	2390.826042	46.5740051
1985	2392.030563	46.5556716
1986	2393.235442	46.53759
1987	2394.440739	46.5200119
1988	2395.644388	46.5018348
1989	2396.848445	46.484291
1990	2398.052993	46.4656524
1991	2399.258206	46.4482765
1992	2400.463312	46.4307785
1993	2401.667411	46.4129028
1994	2402.871586	46.3959083
1995	2404.075113	46.3782577
1996	2405.280818	46.3602676
1997	2406.485895	46.3421554
1998	2407.691634	46.32444
1999	2408.941267	46.3057441

2000	2410.145583	46.286827
2001	2411.349806	46.2679138
2002	2412.555285	46.2487678
2003	2413.759424	46.230442
2004	2414.965261	46.2121849
2005	2416.169299	46.193901
2006	2417.373958	46.1756324
2007	2418.578081	46.1572761
2008	2419.782992	46.138256
2009	2420.988023	46.1193771
2010	2422.192044	46.1012535
2011	2423.395476	46.0831909
2012	2424.600133	46.064846
2013	2425.804836	46.0464324
2014	2427.008693	46.0280876
2015	2428.213413	46.009407
2016	2429.417684	45.9916191
2017	2430.622635	45.9732627
2018	2431.827658	45.955204
2019	2433.032484	45.93787
2020	2434.237337	45.9200782
2021	2435.44107	45.9021453
2022	2436.644944	45.8844947
2023	2437.849394	45.8664894
2024	2439.05368	45.8487434
2025	2440.258435	45.8309478
2026	2441.464276	45.812664
2027	2442.667813	45.7952003
2028	2443.871962	45.7783889
2029	2445.075941	45.7607002
2030	2446.280327	45.7427749
2031	2447.484158	45.7252464
2032	2448.687669	45.7074279
2033	2449.891345	45.6898536
2034	2451.096139	45.6720199
2035	2452.300991	45.6544876
2036	2453.504997	45.6365776
2037	2454.709621	45.6186523
2038	2455.914262	45.6002769
2039	2457.120095	45.5822296
2040	2458.32507	45.5646781
2041	2459.530234	45.5468902
2042	2460.735025	45.5296478
2043	2461.939324	45.5120849
2044	2463.142729	45.4943618
2045	2464.347451	45.4763298
2046	2465.552439	45.4590263
2047	2466.756443	45.4412651
2048	2467.961887	45.423851
2049	2469.166771	45.4062728

2050	2470.37083	45.3889846
2051	2471.575476	45.3718185
2052	2472.779959	45.3549804
2053	2473.984054	45.3381271
2054	2475.188296	45.3214111
2055	2476.393402	45.3048171
2056	2477.597755	45.2882919
2057	2478.802397	45.272129
2058	2480.007031	45.2553062
2059	2481.212995	45.2387695
2060	2482.417383	45.2219429
2061	2483.62143	45.2047996
2062	2484.825499	45.1873054
2063	2486.030131	45.1705741
2064	2487.23563	45.1537818
2065	2488.440792	45.1369018
2066	2489.645201	45.1203231
2067	2490.849589	45.1036605
2068	2492.053976	45.0858688
2069	2493.258319	45.0679588
2070	2494.46327	45.0501213
2071	2495.667546	45.0324745
2072	2496.874355	45.0146942
2073	2498.078364	44.9962425
2074	2499.282354	44.9778213
2075	2500.486992	44.9600677
2076	2501.692481	44.9411811
2077	2502.897362	44.9244613
2078	2504.100857	44.9087638
2079	2505.305297	44.8932647
2080	2506.510172	44.8778266
2081	2507.715519	44.8616256
2082	2508.91943	44.8456001
2083	2510.133541	44.8304214
2084	2511.344986	44.8151588
2085	2512.548844	44.7993087
2086	2513.753799	44.7843589
2087	2514.958504	44.7675132
2088	2516.161956	44.7504043
2089	2517.366385	44.7329177
2090	2518.570828	44.7156524
2091	2519.775988	44.6992874
2092	2520.980444	44.6833381
2093	2522.186278	44.6669921
2094	2523.390401	44.6509323
2095	2524.594438	44.6350288
2096	2525.798529	44.6192054
2097	2527.003257	44.6034393
2098	2528.20742	44.5886154
2099	2529.411008	44.5748863

2100	2530.614401	44.5610008
2101	2531.819467	44.5470848
2102	2533.024185	44.5322647
2103	2534.228692	44.517826
2104	2535.433673	44.5035438
2105	2536.638168	44.4895095
2106	2537.842196	44.4754524
2107	2539.046449	44.4613914
2108	2540.25103	44.4468536
2109	2541.455551	44.4323196
2110	2542.659689	44.4174194
2111	2543.863666	44.4027175
2112	2545.067674	44.3885116
2113	2546.272478	44.3742332
2114	2547.476567	44.3606262
2115	2548.681128	44.3447532
2116	2549.885902	44.3292465
2117	2551.089489	44.314392
2118	2552.293283	44.2998123
2119	2553.497623	44.2849998
2120	2554.703019	44.2705802
2121	2555.916955	44.2566032
2122	2557.121602	44.242485
2123	2558.325019	44.2282867
2124	2559.529981	44.2130203
2125	2560.735437	44.200325
2126	2561.940153	44.1868247
2127	2563.143737	44.1728286
2128	2564.347999	44.158123
2129	2565.552368	44.143608
2130	2566.756099	44.1300544
2131	2567.961115	44.1155776
2132	2569.16602	44.1015625
2133	2570.370399	44.0877265
2134	2571.574595	44.0743255
2135	2572.778436	44.0609893
2136	2573.982672	44.0483512
2137	2575.187218	44.0355911
2138	2576.392253	44.0238151
2139	2577.596137	44.0121078
2140	2578.80008	43.9996261
2141	2580.004252	43.9882431
2142	2581.208959	43.9762763
2143	2582.413242	43.9644546
2144	2583.617942	43.9525451
2145	2584.821503	43.9404792
2146	2586.027084	43.9276428
2147	2587.231335	43.9150161
2148	2588.436487	43.9022064
2149	2589.641943	43.8897323

2150	2590.846164	43.8772163
2151	2592.05054	43.8641967
2152	2593.25425	43.8519363
2153	2594.457902	43.8400726
2154	2595.662244	43.8283805
2155	2596.866349	43.8170661
2156	2598.070039	43.8061447
2157	2599.273684	43.7957687
2158	2600.478222	43.7858581
2159	2601.682473	43.7753028
2160	2602.887453	43.765274
2161	2604.091659	43.7557601
2162	2605.295731	43.7462692
2163	2606.500578	43.7368164
2164	2607.704673	43.7279586
2165	2608.90996	43.7195472
2166	2610.115217	43.7116813
2167	2611.319419	43.7036972
2168	2612.523082	43.6958808
2169	2613.728174	43.6883239
2170	2614.933117	43.681137
2171	2616.137433	43.6732254
2172	2617.341196	43.6662292
2173	2618.54573	43.6588325
2174	2619.749755	43.6511726
2175	2620.953741	43.6434974
2176	2622.158375	43.6367797
2177	2623.363032	43.6297531
2178	2624.568075	43.6230278
2179	2625.772236	43.6163597
2180	2626.976806	43.6111068
2181	2628.181613	43.6067733
2182	2629.386824	43.6021003
2183	2630.591134	43.5985374
2184	2631.794703	43.5949554
2185	2632.999499	43.5919265
2186	2634.204521	43.5881118
2187	2635.409342	43.5852699
2188	2636.61404	43.5823135
2189	2637.818549	43.5795593
2190	2639.022383	43.5760688
2191	2640.22686	43.5726242
2192	2641.432106	43.5691757
2193	2642.637644	43.5651664
2194	2643.843234	43.5615234
2195	2645.047055	43.5576171
2196	2646.251164	43.5543861
2197	2647.455447	43.5511741
2198	2648.660039	43.5490913
2199	2649.864345	43.5473175

2200	2651.068968	43.5462341
2201	2652.272755	43.5449905
2202	2653.476973	43.5445327
2203	2654.681451	43.5437278
2204	2655.886314	43.5441169
2205	2657.091064	43.5447387
2206	2658.295067	43.5451049
2207	2659.500055	43.5448989
2208	2660.704529	43.5441818
2209	2661.908816	43.5428352
2210	2663.112561	43.5408706
2211	2664.316892	43.5395317
2212	2665.521188	43.5375137
2213	2666.726115	43.5365447
2214	2667.930626	43.534645
2215	2669.135982	43.5329093
2216	2670.340641	43.5318717
2217	2671.544682	43.5310516
2218	2672.748182	43.5309333
2219	2673.952173	43.5316658
2220	2675.15735	43.5324821
2221	2676.360682	43.5333023
2222	2677.564489	43.5340003
2223	2678.768154	43.5351181
2224	2679.971982	43.5367469
2225	2681.17589	43.5376319
2226	2682.379995	43.5378494
2227	2683.585011	43.5372543
2228	2684.789046	43.5364913
2229	2685.992764	43.5351753
2230	2687.19701	43.5338439
2231	2688.401989	43.5326423
2232	2689.606967	43.5315208
2233	2690.811535	43.5301284
2234	2692.01658	43.5273208
2235	2693.222582	43.5246505
2236	2694.427412	43.5219917
2237	2695.632262	43.5202636
2238	2696.836734	43.5181503
2239	2698.040316	43.5170364
2240	2699.243578	43.5154953
2241	2700.447948	43.5130767
2242	2701.652668	43.5112686
2243	2702.857489	43.5094833
2244	2704.062342	43.5086441
2245	2705.267197	43.5076484
2246	2706.471311	43.5065841
2247	2707.676243	43.5058517
2248	2708.881023	43.5049018
2249	2710.085511	43.503334

2250	2711.289901	43.5021171
2251	2712.493501	43.5013046
2252	2713.697655	43.5011672
2253	2714.90219	43.499752
2254	2716.107051	43.4989204
2255	2717.312643	43.4983062
2256	2718.52248	43.4976119
2257	2719.726692	43.4965171
2258	2720.931486	43.4957046
2259	2722.136649	43.4944801
2260	2723.341871	43.4926986
2261	2724.546597	43.4914398
2262	2725.75103	43.4895782
2263	2726.955056	43.4875793
2264	2728.159788	43.4851913
2265	2729.364594	43.4830284
2266	2730.568849	43.4806671
2267	2731.774314	43.478321
2268	2732.978556	43.4755821
2269	2734.182881	43.4727249
2270	2735.388069	43.4703178
2271	2736.592947	43.4675598
2272	2737.797538	43.4641151
2273	2739.000762	43.4614562
2274	2740.204526	43.4581375
2275	2741.409103	43.4549446
2276	2742.613508	43.4520492
2277	2743.818901	43.4492721
2278	2745.022681	43.4460372
2279	2746.226897	43.4427032
2280	2747.432039	43.4392929
2281	2748.637165	43.4351196
2282	2749.840592	43.4306793
2283	2751.044793	43.4258117
2284	2752.249298	43.4220428
2285	2753.453206	43.4178237
2286	2754.657178	43.4135284
2287	2755.862269	43.4088439
2288	2757.06733	43.4047164
2289	2758.272261	43.4005928
2290	2759.475852	43.3959274
2291	2760.679803	43.3918762
2292	2761.884792	43.3886756
2293	2763.08938	43.3849067
2294	2764.294393	43.3808403
2295	2765.497824	43.3767013
2296	2766.701867	43.3726539
2297	2767.906571	43.3679389
2298	2769.111884	43.3633117
2299	2770.316687	43.3590393

2300	2771.521473	43.3553886
2301	2772.726145	43.3514556
2302	2773.930072	43.3472061
2303	2775.135039	43.3436775
2304	2776.339075	43.3388442
2305	2777.543964	43.333599
2306	2778.748107	43.3286552
2307	2779.951678	43.3236694
2308	2781.15617	43.3188018
2309	2782.360102	43.3134689
2310	2783.564103	43.3076324
2311	2784.768408	43.3025588
2312	2785.972733	43.2966346
2313	2787.176919	43.2911453
2314	2788.381543	43.2859039
2315	2789.586368	43.2813796
2316	2790.7913	43.2756004
2317	2791.995831	43.2705154
2318	2793.200976	43.2643661
2319	2794.40592	43.2587432
2320	2795.610821	43.2530937
2321	2796.814779	43.2471809
2322	2798.019615	43.2412414
2323	2799.223917	43.2347335
2324	2800.427866	43.2289619
2325	2801.631892	43.222927
2326	2802.836962	43.2173347
2327	2804.041313	43.2113876
2328	2805.246189	43.2060432
2329	2806.449973	43.2002105
2330	2807.654656	43.1946792
2331	2808.866988	43.1884002
2332	2810.071623	43.1826248
2333	2811.276201	43.1764678
2334	2812.481027	43.1695175
2335	2813.685315	43.1628723
2336	2814.889403	43.1558761
2337	2816.093799	43.1494102
2338	2817.298525	43.1422653
2339	2818.504388	43.1350936
2340	2819.708673	43.1274833
2341	2820.912783	43.1199264
2342	2822.116934	43.1126823
2343	2823.322431	43.1061325
2344	2824.527084	43.0997047
2345	2825.731649	43.0926361
2346	2826.935771	43.0855445
2347	2828.140251	43.0786209
2348	2829.345387	43.0717239
2349	2830.549868	43.0653762

2350	2831.754361	43.0582237
2351	2832.95912	43.0519752
2352	2834.16358	43.0452499
2353	2835.367662	43.0373458
2354	2836.572698	43.0293807
2355	2837.776765	43.0219345
2356	2838.980873	43.0147323
2357	2840.185301	43.007225
2358	2841.389348	42.9995803
2359	2842.592936	42.991497
2360	2843.796623	42.9836959
2361	2845.000422	42.9749259
2362	2846.204453	42.9661483
2363	2847.408698	42.9583587
2364	2848.612508	42.9508476
2365	2849.81752	42.943161
2366	2851.022713	42.9345779
2367	2852.227528	42.9258422
2368	2853.432102	42.9176521
2369	2854.636361	42.9097175
2370	2855.839894	42.902378
2371	2857.044164	42.8957443
2372	2858.249366	42.8888015
2373	2859.453049	42.8816642
2374	2860.657512	42.8738632
2375	2861.861562	42.8660507
2376	2863.065553	42.8588485
2377	2864.269489	42.8518486
2378	2865.474116	42.8435401
2379	2866.678657	42.8350028
2380	2867.88212	42.8262405
2381	2869.086377	42.8163414
2382	2870.292022	42.8057022
2383	2871.496677	42.7954483
2384	2872.701658	42.7861366
2385	2873.905427	42.7758369
2386	2875.109389	42.7660598
2387	2876.314043	42.7568435
2388	2877.518737	42.7489547
2389	2878.723999	42.7419662
2390	2879.929195	42.7349052
2391	2881.134341	42.7281417
2392	2882.338008	42.7224502
2393	2883.543233	42.7157402
2394	2884.747368	42.7095108
2395	2885.95183	42.7034339
2396	2887.155992	42.696331
2397	2888.360093	42.6884841
2398	2889.565449	42.6801872
2399	2890.773475	42.6708145

2400	2891.977467	42.6619186
2401	2893.182086	42.6532402
2402	2894.386294	42.644226
2403	2895.590871	42.6352081
2404	2896.795101	42.6253051
2405	2898.000316	42.6153259
2406	2899.20491	42.6068878
2407	2900.409103	42.5980415
2408	2901.612237	42.589508
2409	2902.816241	42.5814132
2410	2904.020764	42.5727424
2411	2905.22647	42.5641593
2412	2906.430916	42.5555801
2413	2907.635592	42.5477523
2414	2908.839432	42.5391006
2415	2910.04378	42.5315208
2416	2911.24806	42.5231018
2417	2912.453079	42.5144233
2418	2913.666574	42.5050506
2419	2914.870385	42.4961433
2420	2916.074497	42.4864501
2421	2917.27924	42.4771347
2422	2918.484419	42.467781
2423	2919.689151	42.457592
2424	2920.893682	42.4482002
2425	2922.097526	42.4382286
2426	2923.301636	42.4287376
2427	2924.506522	42.4185447
2428	2925.711788	42.4088859
2429	2926.916639	42.3981857
2430	2928.119851	42.3887596
2431	2929.324127	42.3788833
2432	2930.529636	42.3688392
2433	2931.734069	42.3599395
2434	2932.939	42.350914
2435	2934.144305	42.3417701
2436	2935.34814	42.3325653
2437	2936.554139	42.3243179
2438	2937.759278	42.3159751
2439	2938.964199	42.3081817
2440	2940.168942	42.2996482
2441	2941.373323	42.2907333
2442	2942.57758	42.2815132
2443	2943.781707	42.2726211
2444	2944.986391	42.2633247
2445	2946.192264	42.2547416
2446	2947.396678	42.2447128
2447	2948.600444	42.2347488
2448	2949.804523	42.2239151
2449	2951.008889	42.2132339

2450	2952.213991	42.2026367
2451	2953.418952	42.1915016
2452	2954.622853	42.1801261
2453	2955.827357	42.167778
2454	2957.032234	42.1555595
2455	2958.237163	42.1429443
2456	2959.441478	42.1306838
2457	2960.645633	42.1179542
2458	2961.849481	42.1065597
2459	2963.054256	42.0948371
2460	2964.257233	42.0833244
2461	2965.462019	42.0719223
2462	2966.666146	42.0613594
2463	2967.870278	42.0494804
2464	2969.0746	42.0376586
2465	2970.278937	42.0257072
2466	2971.483509	42.0138397
2467	2972.687915	42.0018501
2468	2973.892073	41.9888534
