# Supplementary material for: An Ultra-Compact and Low-Cost LAMP-Based Virus Detection Device
Source: Sensors (Basel). 2024 Jul 29;24(15):4912. doi: 10.3390/s24154912 (PMC11314854; doi:10.3390/s24154912)
Supplement: Supplementary file 1 [file sensors-24-04912-s001.zip › One time release .pdf]

Number	Time(s)	Temperature (° C)
1	0.0064786	25.7213478
2	1.2107368	25.7403182
3	2.4158264	25.7999343
4	3.6204187	25.847557
5	4.825823	25.881237
6	6.0316349	25.8999347
7	7.2366408	25.9126873
8	8.4419347	25.9206466
9	9.6462358	25.9278755
10	10.8508656	25.9338626
11	12.0555026	25.9599418
12	13.2595644	25.9810218
13	14.464548	25.9906501
14	15.6689049	25.9947204
15	16.8734536	25.9982032
16	18.0772796	26.0064945
17	19.2816958	26.0180549
18	20.486214	26.0279426
19	21.6904339	26.0377998
20	22.8954004	26.0468082
21	24.100574	26.0574989
22	25.3049811	26.0694847
23	26.5091167	26.0797176
24	27.7137469	26.072565
25	28.9196431	26.0711612
26	30.1243486	26.0625305
27	31.329005	26.0573825
28	32.5334648	26.0551567
29	33.7380991	26.0590267
30	34.9427047	26.0590095
31	36.1471068	26.059061
32	37.3516179	26.0574684
33	38.5568026	26.0553588
34	39.7613763	26.0703067
35	40.9655384	26.0783481
36	42.1693146	26.0914878
37	43.3744224	26.0988559
38	44.5784362	26.1055755
39	45.7826545	26.1049385
40	46.9878647	26.1126003
41	48.1925399	26.1315956
42	49.3967223	26.1531944
43	50.6018856	26.1603946
44	51.8058993	26.1554527
45	53.0105682	26.1620788
46	54.2154378	26.169979
47	55.4204878	26.1603698
48	56.6242075	26.1593933

49	57.8285491	26.1608772
50	59.034221	26.1589508
51	60.2397422	26.1462097
52	61.4442981	26.1339607
53	62.6488984	26.1356773
54	63.8541603	26.1491603
55	65.0579923	26.1511077
56	66.2626296	26.1532859
57	67.4679582	26.1737422
58	68.6725943	26.1880855
59	69.8770254	26.2013454
60	71.0817134	26.2147979
61	72.2869974	26.2335319
62	73.4920911	26.2858428
63	74.6964011	26.3539886
64	75.9002611	26.409954
65	77.1056336	26.4386749
66	78.3108434	26.5029335
67	79.5155312	26.5714931
68	80.7204675	26.6372413
69	81.924184	26.6697635
70	83.1281293	26.7153053
71	84.3326496	26.7568969
72	85.537564	26.7650547
73	86.7412008	26.7572517
74	87.9456274	26.7613182
75	89.1495161	26.7938194
76	90.3531178	26.7902526
77	91.5572328	26.7827568
78	92.7615195	26.7754878
79	93.9663361	26.8027381
80	95.1714723	26.820343
81	96.3758295	26.8366546
82	97.580302	26.8549003
83	98.7847714	26.8734226
84	99.9895245	26.8941345
85	101.1943534	26.915863
86	102.3994219	26.93861
87	103.6031297	26.9622554
88	104.8069787	26.9894142
89	106.0107569	27.0177211
90	107.2145447	27.0458278
91	108.418874	27.0758628
92	109.6229375	27.1063327
93	110.8271353	27.1384391
94	112.0314493	27.1718177
95	113.2357757	27.20611
96	114.4399507	27.2446651
97	115.6448807	27.2849483
98	116.848017	27.3351535

99	118.0527978	27.3863868
100	119.2567665	27.4429054
101	120.4612644	27.4985389
102	121.6660241	27.5547847
103	122.8704662	27.6100063
104	124.0744453	27.664505
105	125.279136	27.7208213
106	126.4834313	27.7741794
107	127.6878816	27.8301811
108	128.8920205	27.8783302
109	130.0964851	27.9316596
110	131.3005757	27.9830894
111	132.5045615	28.0398101
112	133.7095991	28.0989494
113	134.9146591	28.1638069
114	136.1202033	28.2329101
115	137.3235754	28.3052406
116	138.5271989	28.3842201
117	139.731722	28.4679298
118	140.9366539	28.5610923
119	142.1415859	28.6594181
120	143.3466661	28.7668952
121	144.5508864	28.8833427
122	145.7558694	29.0115451
123	146.9612314	29.1656951
124	148.166832	29.3400421
125	149.3717549	29.5389308
126	150.5767391	29.7531185
127	151.7820412	29.9984092
128	152.9860665	30.2656154
129	154.1910307	30.5712432
130	155.3962007	30.919485
131	156.5996236	31.3015441
132	157.8074666	31.7305259
133	159.012252	32.1872825
134	160.2167412	32.6854248
135	161.4205625	33.2166137
136	162.6252367	33.7930068
137	163.8299574	34.4046516
138	165.0345349	35.0519561
139	166.2392022	35.7304763
140	167.4432515	36.4314727
141	168.6474019	37.1657333
142	169.8518116	37.9238433
143	171.0564458	38.7343177
144	172.2611932	39.5731124
145	173.4658712	40.43684
146	174.6707297	41.3340072
147	175.8753079	42.2661361
148	177.0802925	43.2174148

149	178.2856116	44.1647148
150	179.4897056	45.1282196
151	180.6944976	46.1278572
152	181.8999102	47.0989265
153	183.1048454	48.085186
154	184.31041	49.0950622
155	185.5149039	50.1330261
156	186.7199066	51.184288
157	187.9248598	52.2434158
158	189.1302374	53.3210334
159	190.3351653	54.4302558
160	191.5397488	55.5906372
161	192.744219	56.7639846
162	193.9487618	57.9006843
163	195.1530943	58.90625
164	196.3586757	59.8252944
165	197.5640613	60.6499252
166	198.7685442	61.3960952
167	199.9735264	62.0793647
168	201.1786233	62.7110519
169	202.3836376	63.3347778
170	203.5887753	63.9045219
171	204.793141	64.4302825
172	205.9976351	65.0060119
173	207.2023368	65.6292572
174	208.4076541	66.2463912
175	209.6127424	66.8855209
176	210.8177572	67.5391464
177	212.0224258	68.1973648
178	213.2271952	68.859703
179	214.4317105	69.4772796
180	215.6361176	70.0566635
181	216.8414675	70.6055755
182	218.0461557	71.1302108
183	219.2496017	71.6543121
184	220.4529185	72.1875457
185	221.6581899	72.7094955
186	222.8628175	73.225151
187	224.0665351	73.7189483
188	225.2704457	74.1903915
189	226.4745787	74.6391448
190	227.6786264	75.0643234
191	228.8832863	75.4664154
192	230.0883738	75.8535308
193	231.2936433	76.2358551
194	232.4973811	76.615158
195	233.7025313	76.9920196
196	234.9069648	77.3554611
197	236.1116421	77.7123947
198	237.3168528	78.0631561

199	238.5205925	78.410202
200	239.7252391	78.7569122
201	240.9301344	79.1006011
202	242.1346547	79.4351577
203	243.3391334	79.7565689
204	244.5429239	80.0701522
205	245.7468123	80.3778381
206	246.9515044	80.6760787
207	248.1565521	80.9656524
208	249.3610553	81.2461547
209	250.5660494	81.5207672
210	251.7704822	81.7826995
211	252.975058	82.0294647
212	254.1796359	82.2647247
213	255.3853732	82.487152
214	256.5901537	82.6942977
215	257.7946324	82.8913497
216	258.9990832	83.0811309
217	260.2027335	83.2573776
218	261.4076908	83.4231796
219	262.6123805	83.5765151
220	263.816993	83.7213973
221	265.022114	83.8616561
222	266.2260595	83.9990768
223	267.4308425	84.139122
224	268.6358109	84.2790832
225	269.8411262	84.4149703
226	271.0462109	84.5460968
227	272.2502009	84.6783599
228	273.455463	84.8124465
229	274.6601811	84.9469833
230	275.8650621	85.0829925
231	277.0704753	85.2227783
232	278.2753993	85.363739
233	279.4802358	85.4981079
234	280.6852881	85.6309585
235	281.8892866	85.7656173
236	283.0940872	85.906166
237	284.299295	86.0414657
238	285.504435	86.1823577
239	286.7094286	86.3176193
240	287.9143057	86.4474868
241	289.1191484	86.5700302
242	290.3242047	86.6888275
243	291.5290399	86.8088607
244	292.7336042	86.9270248
245	293.9377976	87.0387496
246	295.1426071	87.1407241
247	296.3485766	87.2447357
248	297.5531679	87.3367462

249	298.7573723	87.425354
250	299.9630117	87.5144424
251	301.1680169	87.6017608
252	302.3731968	87.6863403
253	303.5781762	87.767189
254	304.7829562	87.8464431
255	305.9872977	87.9227828
256	307.1923788	87.9966659
257	308.3967237	88.067543
258	309.6008457	88.1357498
259	310.8051373	88.2043075
260	312.0087004	88.2709579
261	313.2135226	88.3352508
262	314.4185432	88.4010467
263	315.6221145	88.4644622
264	316.8263252	88.5261077
265	318.033559	88.5874023
266	319.2374331	88.6472015
267	320.4414773	88.7085723
268	321.6459255	88.7639617
269	322.8509395	88.8186645
270	324.0562017	88.8718719
271	325.2610693	88.9241561
272	326.4646411	88.9719696
273	327.6703175	89.0171432
274	328.8744714	89.0623626
275	330.0785571	89.1058502
276	331.2830659	89.1468658
277	332.4869829	89.1833419
278	333.6918362	89.2207183
279	334.896641	89.2561264
280	336.1016184	89.2908172
281	337.3077075	89.3222885
282	338.5121765	89.3585891
283	339.7177296	89.3993911
284	340.9223015	89.4372711
285	342.127129	89.4750289
286	343.3315902	89.5098876
287	344.5367644	89.5482254
288	345.7419197	89.58358
289	346.9459716	89.6158752
290	348.1505898	89.6487579
291	349.3546956	89.6808395
292	350.5593649	89.7054901
293	351.762786	89.7253646
294	352.9676037	89.7458953
295	354.1718804	89.764923
296	355.3767172	89.7852325
297	356.5821234	89.8111419
298	357.7871721	89.8336181

299	358.9913229	89.8571243
300	360.1954406	89.8770599
301	361.4003528	89.8970489
302	362.6049622	89.9194183
303	363.8099182	89.936943
304	365.0154704	89.953125
305	366.2191475	89.9672393
306	367.4236875	89.9807128
307	368.6293079	89.9806289
308	369.8341888	89.9852905
309	371.0398361	89.9857025
310	372.2445209	89.9877166
311	373.448672	89.9883575
312	374.6538973	89.9834747
313	375.8573676	89.9800338
314	377.0621996	89.9747009
315	378.2670863	89.97052
316	379.4720723	89.9643402
317	380.6756586	89.9565277
318	381.881389	89.9513702
319	383.0860432	89.9450378
320	384.2907916	89.9335174
321	385.4956508	89.9222564
322	386.6999203	89.9095535
323	387.9046427	89.8957214
324	389.1090594	89.8818817
325	390.3139628	89.8659515
326	391.5188232	89.8477554
327	392.7226886	89.8309249
328	393.9266974	89.8076629
329	395.1321284	89.7838973
330	396.3361786	89.7634811
331	397.540443	89.7426986
332	398.7453442	89.7222518
333	399.9492426	89.7032089
334	401.1538286	89.6811981
335	402.3584341	89.6632843
336	403.5632684	89.6445846
337	404.7679989	89.6228408
338	405.9730077	89.6011657
339	407.1771694	89.5810699
340	408.3816013	89.5562362
341	409.5854687	89.5282669
342	410.7891253	89.5000839
343	411.9928962	89.4700927
344	413.1967444	89.4386749
345	414.4011841	89.400711
346	415.6061859	89.3634719
347	416.811855	89.3276519
348	418.0174974	89.2911758

349	419.2220936	89.2526855
350	420.4264325	89.215538
351	421.6320194	89.1779708
352	422.8367308	89.1397171
353	424.0416967	89.0991592
354	425.2461457	89.061882
355	426.4514721	89.0239105
356	427.6559474	88.9851455
357	428.8606386	88.944168
358	430.0654747	88.9032669
359	431.269553	88.8727264
360	432.4733712	88.8391265
361	433.6772836	88.8060073
362	434.8821062	88.7700347
363	436.0864711	88.7346572
364	437.290599	88.6964187
365	438.4951029	88.655838
366	439.6988242	88.6155776
367	440.9034666	88.5760498
368	442.1077608	88.535614
369	443.3124553	88.4840774
370	444.5172364	88.4348449
371	445.721774	88.3831253
372	446.9258709	88.3347244
373	448.1298633	88.2855453
374	449.3342877	88.2354507
375	450.5390359	88.1881942
376	451.7443442	88.1406478
377	452.9491644	88.0900115
378	454.1529228	88.0384368
379	455.3576051	87.9871368
380	456.5615118	87.9342422
381	457.7663272	87.8826446
382	458.9716409	87.8390197
383	460.1770249	87.7899932
384	461.3834559	87.740509
385	462.5887646	87.6914443
386	463.7942649	87.6418304
387	464.9982971	87.5919342
388	466.2030041	87.542984
389	467.4067912	87.4939193
390	468.6110012	87.4448699
391	469.8152404	87.3953323
392	471.0207657	87.3362884
393	472.2259113	87.2814559
394	473.429898	87.229248
395	474.6346293	87.1752777
396	475.8394069	87.1203231
397	477.0434973	87.0680923
398	478.2487459	87.0154113

399	479.4532749	86.9621124
400	480.657685	86.9087524
401	481.8630522	86.8556594
402	483.0678153	86.8035354
403	484.2730006	86.75634
404	485.4781444	86.7094421
405	486.682057	86.6584472
406	487.8860857	86.6066589
407	489.0910805	86.5544357
408	490.2959076	86.5027008
409	491.500943	86.451271
410	492.7057523	86.4003601
411	493.9102481	86.349266
412	495.114414	86.2977371
413	496.3196495	86.2409133
414	497.5249652	86.1833801
415	498.7293856	86.1283187
416	499.934554	86.0764083
417	501.1393304	86.0237731
418	502.3437212	85.9701843
419	503.5493996	85.9166107
420	504.7546494	85.8629913
421	505.9593274	85.8148345
422	507.1646826	85.7640838
423	508.3692682	85.7130508
424	509.5740659	85.6593704
425	510.7792931	85.6075744
426	511.9836199	85.5547409
427	513.1887473	85.5020599
428	514.3931575	85.4493865
429	515.5987416	85.3975601
430	516.8036162	85.3464202
431	518.0082455	85.2903289
432	519.2133919	85.2372894
433	520.4186804	85.1858062
434	521.6234491	85.1356811
435	522.828884	85.0845794
436	524.033827	85.0339508
437	525.2382122	84.9844512
438	526.4432004	84.9351348
439	527.6476356	84.8853454
440	528.8516884	84.833496
441	530.0559495	84.7818756
442	531.2610099	84.729988
443	532.4662661	84.6775817
444	533.6702392	84.6237411
445	534.8743419	84.5719528
446	536.0800372	84.518753
447	537.285381	84.4653472
448	538.489761	84.4130706

449	539.6945873	84.3605041
450	540.8992242	84.3089218
451	542.1025436	84.2580184
452	543.3075512	84.2061996
453	544.5125336	84.1548538
454	545.7177381	84.1057281
455	546.9225459	84.0558395
456	548.1268748	84.0056304
457	549.3311965	83.9568862
458	550.5355889	83.9071502
459	551.7406609	83.8581237
460	552.9449893	83.8092346
461	554.149325	83.7597503
462	555.353398	83.7104034
463	556.5579857	83.6608963
464	557.7626223	83.611206
465	558.9666112	83.5600738
466	560.1704993	83.5112609
467	561.3747451	83.4603424
468	562.5788139	83.4100112
469	563.7826865	83.3591384
470	564.9874914	83.3085479
471	566.1934732	83.2580947
472	567.398556	83.2086029
473	568.6031544	83.1591567
474	569.8073574	83.1100997
475	571.0118523	83.0625839
476	572.2160967	83.0137786
477	573.4198147	82.9650115
478	574.6233065	82.9164428
479	575.8278643	82.8676757
480	577.0313577	82.8189849
481	578.2365896	82.7700042
482	579.4412806	82.7203903
483	580.6463065	82.6712493
484	581.8500003	82.6210174
485	583.0558376	82.5710983
486	584.2606783	82.5221939
487	585.4663446	82.4736557
488	586.6712826	82.4250793
489	587.8753519	82.3771896
490	589.0793677	82.3289947
491	590.2830957	82.2810516
492	591.4875866	82.2327423
493	592.6935665	82.1859588
494	593.8987737	82.1383056
495	595.1035891	82.090744
496	596.3081515	82.0421981
497	597.5123972	81.9935302
498	598.7172792	81.9452056

499	599.9222761	81.8962249
500	601.1272168	81.8465042
501	602.3320736	81.7973327
502	603.5364976	81.7483749
503	604.7427147	81.6967468
504	605.9476634	81.6465454
505	607.1523235	81.5963745
506	608.3568255	81.5471954
507	609.5610675	81.4974288
508	610.7659936	81.4479141
509	611.971203	81.3987274
510	613.1764528	81.3505477
511	614.3816627	81.3015899
512	615.5856163	81.2539672
513	616.7891863	81.2072982
514	617.9930519	81.1605072
515	619.1969026	81.1131057
516	620.4023241	81.0661163
517	621.6077704	81.0200119
518	622.8121318	80.973999
519	624.0164019	80.9281311
520	625.2213471	80.8825149
521	626.4252108	80.8367767
522	627.6302904	80.7906341
523	628.8348168	80.7439193
524	630.0393851	80.6972274
525	631.2440418	80.6502456
526	632.4488802	80.603096
527	633.6541779	80.5560379
528	634.8588888	80.5085601
529	636.0635605	80.4612884
530	637.2681329	80.4141387
531	638.4732733	80.3677368
532	639.6771458	80.3215484
533	640.8812551	80.2759552
534	642.0865255	80.2306365
535	643.2903361	80.1857757
536	644.4949129	80.1408843
537	645.6998962	80.0961151
538	646.9056396	80.0511016
539	648.1109851	80.0062637
540	649.316034	79.9616165
541	650.5201951	79.9164352
542	651.7252961	79.8713836
543	652.930593	79.8261795
544	654.1358443	79.7812042
545	655.3403571	79.7360992
546	656.5456932	79.6913757
547	657.7506797	79.6444168
548	658.9564504	79.5992279

549	660.1620764	79.5543975
550	661.3668201	79.5093002
551	662.5712567	79.464859
552	663.7762763	79.4200897
553	664.9812961	79.3752593
554	666.1864137	79.330368
555	667.3919941	79.2859191
556	668.5965292	79.2407989
557	669.8018638	79.1980209
558	671.0067799	79.1543121
559	672.2119429	79.1104965
560	673.4168434	79.0666198
561	674.6220103	79.0227127
562	675.8261111	78.9782714
563	677.0304504	78.9345474
564	678.2349323	78.8910598
565	679.4391373	78.8473892
566	680.6446895	78.8032836
567	681.8496813	78.7590789
568	683.0533102	78.7156524
569	684.2581325	78.6725769
570	685.463043	78.6293716
571	686.6675621	78.5891571
572	687.8729716	78.5483474
573	689.0774103	78.5077209
574	690.2809145	78.4667358
575	691.486016	78.4253921
576	692.6917485	78.3844909
577	693.8970765	78.3435287
578	695.1020224	78.3016891
579	696.3070654	78.2591705
580	697.5116753	78.2171783
581	698.7169615	78.1725921
582	699.9215576	78.129425
583	701.1267635	78.0857543
584	702.3309369	78.0426788
585	703.5352868	77.9997406
586	704.7400862	77.956726
587	705.9445825	77.9141387
588	707.1485762	77.8718719
589	708.3542206	77.8299713
590	709.5583359	77.7876205
591	710.7627224	77.7451095
592	711.967371	77.7023696
593	713.1718573	77.6599273
594	714.3756365	77.6181869
595	715.5793425	77.5767593
596	716.7832476	77.5356063
597	717.9870876	77.4940643
598	719.1909622	77.4527435

599	720.3952403	77.4119033
600	721.6003124	77.3706665
601	722.8047313	77.3295135
602	724.0090761	77.2882995
603	725.2135254	77.2468414
604	726.4194176	77.2048492
605	727.6240928	77.1627578
606	728.8289879	77.1209259
607	730.0366566	77.0791625
608	731.2412001	77.0375976
609	732.4456458	76.9955291
610	733.6507103	76.9542846
611	734.8558178	76.914093
612	736.0605766	76.8736419
613	737.2644646	76.8347549
614	738.4691763	76.794548
615	739.6748082	76.7550506
616	740.88701	76.7161712
617	742.0926161	76.6772994
618	743.2977566	76.6379623
619	744.5025	76.599205
620	745.7075974	76.5607299
621	746.9123162	76.521347
622	748.1177857	76.4820785
623	749.3229732	76.4421539
624	750.5265411	76.4033508
625	751.7311933	76.3640899
626	752.9357353	76.3266372
627	754.1408929	76.2888412
628	755.3456671	76.2508621
629	756.5498457	76.2126312
630	757.7549775	76.1741561
631	758.9591602	76.1357879
632	760.1638106	76.097763
633	761.3691997	76.0590438
634	762.5741331	76.0200576
635	763.7781516	75.9815216
636	764.9825326	75.9409408
637	766.1869657	75.9075622
638	767.3925712	75.8703918
639	768.597235	75.8327178
640	769.8022322	75.7941284
641	771.0071672	75.7550582
642	772.2121397	75.7163848
643	773.41585	75.6779022
644	774.6208113	75.6399688
645	775.8251147	75.6012115
646	777.0288527	75.5624389
647	778.2340038	75.5173492
648	779.4392308	75.4764862

649	780.6447987	75.4368286
650	781.8499502	75.3975524
651	783.0552896	75.3585815
652	784.2600216	75.3202896
653	785.4649946	75.281639
654	786.6693022	75.2431106
655	787.8742404	75.206726
656	789.0793694	75.1942367
657	790.2840156	75.3932571
658	791.4956639	75.554367
659	792.7012645	75.7037506
660	793.9068435	75.8575744
661	795.1117706	76.0107574
662	796.3167083	76.1633148
663	797.5206561	76.3179779
664	798.7261011	76.4705886
665	799.9315471	76.6191101
666	801.136478	76.7400894
667	802.3409889	76.6479263
668	803.6043874	76.5935821
669	804.8088137	76.5493621
670	806.0133078	76.5018615
671	807.2187886	76.4553375
672	808.4237732	76.409027
673	809.6290895	76.3608474
674	810.8336948	76.314537
675	812.0374934	76.2698822
676	813.243332	76.2274169
677	814.449395	76.1858673
678	815.6538213	76.152153
679	816.8576978	76.1316223
680	818.0621485	76.1139068
681	819.2675088	76.0976486
682	820.4719962	76.0758438
683	821.6773175	76.0507049
684	822.8832365	76.0232162
685	824.0882557	75.9943161
686	825.2929728	75.9649887
687	826.4979081	75.9326629
688	827.7026974	75.8901138
689	828.9090608	75.8324127
690	830.1139025	75.7697372
691	831.3182569	75.7033691
692	832.5222671	75.6398925
693	833.7274547	75.5782928
694	834.9327566	75.5175018
695	836.1376172	75.4571685
696	837.3424344	75.3979949
697	838.5469595	75.3408966
698	839.7508541	75.2852172

699	840.9554851	75.2307891
700	842.1597097	75.1769866
701	843.363519	75.1246261
702	844.5673334	75.0738067
703	845.7721151	75.0240631
704	846.9777089	74.9747772
705	848.1817814	74.9263763
706	849.387088	74.8780517
707	850.5911459	74.828804
708	851.7953741	74.779747
709	853.0006549	74.7324752
710	854.2055173	74.6864776
711	855.4106487	74.6401367
712	856.6157137	74.5941772
713	857.8194804	74.5474853
714	859.0230807	74.5013809
715	860.2277472	74.4555053
716	861.4326225	74.4092636
717	862.6376878	74.3650283
718	863.8425396	74.3204498
719	865.0467291	74.2746734
720	866.251609	74.2284164
721	867.4564652	74.1822357
722	868.6615675	74.1357269
723	869.8661166	74.0899734
724	871.0704824	74.043869
725	872.2748298	73.9979705
726	873.4801118	73.9524154
727	874.6851732	73.9076309
728	875.8900186	73.8632965
729	877.0946098	73.8198013
730	878.298863	73.7760467
731	879.5038371	73.7328338
732	880.7090419	73.6903457
733	881.9137167	73.6481399
734	883.1194014	73.6062774
735	884.32358	73.5644226
736	885.5276485	73.5228729
737	886.7315272	73.4812774
738	887.9369072	73.4397125
739	889.1420424	73.3995361
740	890.3468439	73.3601074
741	891.5512267	73.3204498
742	892.7557634	73.2806854
743	893.9612338	73.2415542
744	895.1664299	73.2026977
745	896.3717928	73.1635742
746	897.575287	73.1243743
747	898.7795494	73.0847396
748	899.9839286	73.0458374

749	901.1892557	73.0052642
750	902.3937478	72.9648056
751	903.5990037	72.9241256
752	904.802722	72.8833694
753	906.0077944	72.842453
754	907.2122499	72.8017959
755	908.4177264	72.7628631
756	909.6229659	72.7237396
757	910.8271539	72.6851348
758	912.0314125	72.6460418
759	913.236573	72.6077117
760	914.4412613	72.5689697
761	915.6466501	72.5314559
762	916.8512082	72.493843
763	918.0554995	72.4562988
764	919.2598453	72.4186325
765	920.4651325	72.3802108
766	921.6698896	72.3418731
767	922.8752867	72.303543
768	924.0798789	72.2659225
769	925.2844705	72.2288665
770	926.4887549	72.191719
771	927.6935384	72.1542968
772	928.8984398	72.1172409
773	930.1044375	72.0801849
774	931.3094141	72.0438461
775	932.5139086	72.0066528
776	933.7185086	71.970581
777	934.9231713	71.9338378
778	936.1284777	71.8973312
779	937.3332117	71.8603057
780	938.5373266	71.8236007
781	939.7426084	71.7866668
782	940.9469781	71.7507553
783	942.1521397	71.714714
784	943.3570694	71.6790084
785	944.5624075	71.6439056
786	945.766555	71.6083984
787	946.9711634	71.5738525
788	948.1771836	71.5390167
789	949.3823174	71.5042266
790	950.5875891	71.4695587
791	951.79262	71.4349517
792	952.9970151	71.3992004
793	954.2022905	71.3631973
794	955.4071606	71.3289566
795	956.6131499	71.2941207
796	957.8184545	71.2591018
797	959.0229062	71.2236404
798	960.2290163	71.1880798

799	961.4336904	71.1525192
800	962.6376791	71.1173248
801	963.8424942	71.0824432
802	965.0470443	71.0482177
803	966.2520845	71.0150299
804	967.4566977	70.9787139
805	968.660801	70.9430694
806	969.866186	70.9081192
807	971.0696669	70.8737945
808	972.2740775	70.8396224
809	973.4775989	70.8052673
810	974.6823339	70.7706985
811	975.8877323	70.7360229
812	977.0930368	70.7010421
813	978.2968789	70.6652832
814	979.500642	70.6309661
815	980.7062472	70.5969696
816	981.9114921	70.562416
817	983.1171979	70.5284576
818	984.3214903	70.4936141
819	985.5256523	70.4582977
820	986.7307044	70.4236679
821	987.9347926	70.3890533
822	989.1397868	70.3541107
823	990.344618	70.320198
824	991.5495347	70.2850265
825	992.7539414	70.2503128
826	993.958343	70.2159729
827	995.163239	70.1812286
828	996.3687395	70.1473617
829	997.5743939	70.1140594
830	998.7782291	70.0804443
831	999.9824456	70.047348
832	1001.188327	70.0141906
833	1002.3942	69.9800643
834	1003.598855	69.9469146
835	1004.897887	69.9133148
836	1006.102057	69.8791046
837	1007.307217	69.8449783
838	1008.513031	69.811264
839	1009.716967	69.77742
840	1010.921402	69.7435302
841	1012.125308	69.7091827
842	1013.330087	69.6757888
843	1014.535388	69.6426925
844	1015.740506	69.6085281
845	1016.945012	69.5752639
846	1018.149059	69.5430755
847	1019.353115	69.5107498
848	1020.55692	69.4784927

849	1021.76076	69.4471054
850	1022.964549	69.4154357
851	1024.168309	69.3844985
852	1025.371986	69.3534393
853	1026.576552	69.3222427
854	1027.782318	69.2915802
855	1028.987963	69.2602691
856	1030.193114	69.2290344
857	1031.398575	69.1973495
858	1032.602627	69.1746749
859	1033.807425	69.1492462
860	1035.012537	69.1211471
861	1036.217291	69.0915145
862	1037.421977	69.0608978
863	1038.626139	69.0299224
864	1039.83025	68.9987945
865	1041.03563	68.967102
866	1042.240665	68.9343719
867	1043.44582	68.902359
868	1044.649494	68.861351
869	1045.854894	68.8226776
870	1047.059794	68.7863616
871	1048.264555	68.7506027
872	1049.469519	68.715393
873	1050.673699	68.681282
874	1051.879023	68.6475143
875	1053.082928	68.6140518
876	1054.287972	68.5808563
877	1055.492653	68.5475616
878	1056.696759	68.5137634
879	1057.9012	68.4798355
880	1059.105425	68.4473037
881	1060.30996	68.4155349
882	1061.514456	68.3840103
883	1062.720285	68.3525314
884	1063.925741	68.320877
885	1065.130873	68.2887268
886	1066.335514	68.2574005
887	1067.540125	68.2253265
888	1068.744542	68.1944656
889	1069.949643	68.163269
890	1071.153692	68.1307144
891	1072.358485	68.0990676
892	1073.563434	68.0663146
893	1074.768611	68.0335845
894	1075.973216	68.0011596
895	1077.178216	67.968666
896	1078.382623	67.9364242
897	1079.587054	67.9044342
898	1080.790914	67.8714981

899	1081.995983	67.8393707
900	1083.200628	67.8083953
901	1084.405164	67.7757186
902	1085.609901	67.7441329
903	1086.814169	67.712326
904	1088.01864	67.6808242
905	1089.22297	67.6498947
906	1090.427837	67.6180343
907	1091.632366	67.5857543
908	1092.836918	67.5540618
909	1094.041615	67.5224685
910	1095.246622	67.4906311
911	1096.451259	67.4591903
912	1097.656357	67.4282073
913	1098.860507	67.3968658
914	1100.064886	67.3653793
915	1101.268856	67.334587
916	1102.472929	67.3034515
917	1103.677325	67.2731933
918	1104.882064	67.2425994
919	1106.087083	67.2119293
920	1107.291367	67.1810684
921	1108.496246	67.1507949
922	1109.701346	67.1197052
923	1110.906128	67.0890045
924	1112.111005	67.0583419
925	1113.315127	67.0269775
926	1114.520166	66.9969253
927	1115.725167	66.966217
928	1116.930193	66.9353637
929	1118.134291	66.9040679
930	1119.343172	66.8736038
931	1120.547605	66.8428878
932	1121.753104	66.8132781
933	1122.958353	66.7827987
934	1124.163389	66.7525787
935	1125.368517	66.7225875
936	1126.573186	66.6919784
937	1127.777781	66.6628723
938	1128.982584	66.6345214
939	1130.187503	66.6079864
940	1131.393359	66.5822601
941	1132.597932	66.5573272
942	1133.803335	66.5339584
943	1135.008237	66.5119247
944	1136.213143	66.4899139
945	1137.418118	66.4683761
946	1138.623498	66.4470214
947	1139.827381	66.4253997
948	1141.030867	66.4032287

949	1142.235171	66.3789825
950	1143.441002	66.35289
951	1144.645399	66.3266754
952	1145.850213	66.2994537
953	1147.054336	66.2710418
954	1148.259843	66.2420959
955	1149.464281	66.2123794
956	1150.668749	66.1822204
957	1151.873768	66.1512374
958	1153.078266	66.120491
959	1154.282924	66.0899505
960	1155.487552	66.0591812
961	1156.693203	66.0281753
962	1157.897647	65.9962997
963	1159.102655	65.9645385
964	1160.307111	65.9332122
965	1161.511113	65.9020919
966	1162.71626	65.8706436
967	1163.921242	65.8391647
968	1165.125822	65.8064651
969	1166.330583	65.7737884
970	1167.535888	65.7413406
971	1168.740407	65.7080688
972	1169.945282	65.6740264
973	1171.150814	65.6400985
974	1172.354471	65.6059112
975	1173.558053	65.5712814
976	1174.76253	65.537445
977	1175.967543	65.5039215
978	1177.172674	65.4721298
979	1178.377846	65.4404144
980	1179.588879	65.4085998
981	1180.793057	65.3773651
982	1181.998197	65.346054
983	1183.20341	65.3151779
984	1184.408244	65.2850799
985	1185.612395	65.2552566
986	1186.816132	65.225357
987	1188.020528	65.1956329
988	1189.225589	65.1658401
989	1190.430826	65.1358718
990	1191.636129	65.1061325
991	1192.841418	65.0765914
992	1194.046854	65.0479736
993	1195.251087	65.0193328
994	1196.455709	64.9907455
995	1197.660648	64.9631805
996	1198.865881	64.9354095
997	1200.070685	64.9070816
998	1201.274592	64.878189

999	1202.478994	64.8501129
1000	1203.683833	64.8220443
1001	1204.889264	64.7941055
1002	1206.093913	64.7657928
1003	1207.298227	64.7378997
1004	1208.50338	64.7100982
1005	1209.707621	64.6818313
1006	1210.912235	64.6540069
1007	1212.117447	64.626358
1008	1213.321926	64.5990982
1009	1214.526062	64.5715255
1010	1215.730651	64.5443878
1011	1216.936323	64.5170593
1012	1218.140862	64.4901809
1013	1219.345995	64.4628601
1014	1220.550149	64.4348602
1015	1221.755671	64.405754
1016	1222.959241	64.37751
1017	1224.164134	64.349472
1018	1225.368787	64.3210983
1019	1226.573669	64.292984
1020	1227.777652	64.2646789
1021	1228.982108	64.2360992
1022	1230.186303	64.2085266
1023	1231.391824	64.1801376
1024	1232.596901	64.1522827
1025	1233.801168	64.1255035
1026	1235.005826	64.0978546
1027	1236.210586	64.0699691
1028	1237.415568	64.0419082
1029	1238.620241	64.0140686
1030	1239.825432	63.9858093
1031	1241.030445	63.9579887
1032	1242.234327	63.9291801
1033	1243.439147	63.9016418
1034	1244.643588	63.8745193
1035	1245.847646	63.8473739
1036	1247.051391	63.8205261
1037	1248.255676	63.7938728
1038	1249.459739	63.7681465
1039	1250.665209	63.742321
1040	1251.869245	63.7169799
1041	1253.074036	63.6913833
1042	1254.278511	63.6661872
1043	1255.4837	63.6396903
1044	1256.68892	63.6121635
1045	1257.894056	63.5847702
1046	1259.098952	63.5571594
1047	1260.303469	63.5295181
1048	1261.507776	63.5009536

1049	1262.712	63.4723396
1050	1263.917108	63.4436569
1051	1265.123033	63.4152412
1052	1266.327734	63.3865127
1053	1267.531773	63.3592605
1054	1268.736328	63.3318595
1055	1269.94186	63.3037185
1056	1271.147769	63.2762756
1057	1272.352629	63.2488822
1058	1273.556726	63.2212524
1059	1274.761163	63.194561
1060	1275.965734	63.1672134
1061	1277.17085	63.1401252
1062	1278.376181	63.1134033
1063	1279.581149	63.0861434
1064	1280.785552	63.0595283
1065	1281.989931	63.0334587
1066	1283.194495	63.0061874
1067	1284.399128	62.9792976
1068	1285.604383	62.9527359
1069	1286.809061	62.9253959
1070	1288.013188	62.8978157
1071	1289.218213	62.8704414
1072	1290.423115	62.8423881
1073	1291.62789	62.8141975
1074	1292.832792	62.7857475
1075	1294.036625	62.7573432
1076	1295.241396	62.729103
1077	1296.445855	62.7010498
1078	1297.651343	62.6730041
1079	1298.855851	62.6446304
1080	1300.059993	62.6175231
1081	1301.264067	62.5894966
1082	1302.468512	62.5615844
1083	1303.672591	62.5340995
1084	1304.878091	62.5062751
1085	1306.083277	62.4788284
1086	1307.287399	62.4513435
1087	1308.491494	62.4236335
1088	1309.695752	62.3968315
1089	1310.900815	62.3701248
1090	1312.105882	62.3436203
1091	1313.309792	62.3177108
1092	1314.515399	62.2916374
1093	1315.719436	62.2654838
1094	1316.923765	62.2399673
1095	1318.128454	62.2143478
1096	1319.333512	62.1886367
1097	1320.53831	62.1631889
1098	1321.742364	62.1370162

1099	1322.945718	62.1114616
1100	1324.149118	62.0847091
1101	1325.352751	62.0580749
1102	1326.556767	62.0318984
1103	1327.760321	62.0057449
1104	1328.964079	61.9789619
1105	1330.168	61.95232
1106	1331.37234	61.9260559
1107	1332.577507	61.8995246
1108	1333.783152	61.8724479
1109	1334.988455	61.8453063
1110	1336.193343	61.8191833
1111	1337.399055	61.7927017
1112	1338.604452	61.7662582
1113	1339.809158	61.7403564
1114	1341.014087	61.7143859
1115	1342.218606	61.688343
1116	1343.423487	61.6622505
1117	1344.62861	61.6360588
1118	1345.834134	61.6107482
1119	1347.039185	61.5851516
1120	1348.242987	61.5584831
1121	1349.448211	61.5325622
1122	1350.653473	61.5063056
1123	1351.858686	61.4799423
1124	1353.064052	61.4545364
1125	1354.269328	61.4291572
1126	1355.475169	61.4043083
1127	1356.680465	61.3797836
1128	1357.885504	61.3550415
1129	1359.090054	61.3304138
1130	1360.295197	61.3059196
1131	1361.500847	61.2811088
1132	1362.704852	61.2564697
1133	1363.910439	61.2308883
1134	1365.114945	61.2052192
1135	1366.319589	61.1792106
1136	1367.52389	61.1529426
1137	1368.728383	61.1264457
1138	1369.932651	61.0996398
1139	1371.136837	61.0731582
1140	1372.341948	61.0472297
1141	1373.547416	61.0210647
1142	1374.751575	60.9948234
1143	1375.956745	60.9695625
1144	1377.160983	60.944664
1145	1378.366234	60.9198493
1146	1379.570845	60.8953056
1147	1380.776139	60.8708724
1148	1381.980806	60.8470344

1149	1383.184719	60.8234024
1150	1384.389511	60.8345565
1151	1385.594787	60.8344841
1152	1386.799365	60.8261222
1153	1388.003745	60.8119506
1154	1389.208336	60.7941169
1155	1390.412939	60.7745552
1156	1391.61841	60.7534255
1157	1392.823771	60.7312088
1158	1394.029191	60.7081985
1159	1395.233784	60.6838073
1160	1396.438902	60.6256179
1161	1397.643482	60.578289
1162	1398.848478	60.5396499
1163	1400.054386	60.5067558
1164	1401.259206	60.4766731
1165	1402.462595	60.4485702
1166	1403.665914	60.4213142
1167	1404.87118	60.3952217
1168	1406.076194	60.3692092
1169	1407.280153	60.3432235
1170	1408.485411	60.3162422
1171	1409.689036	60.2893257
1172	1410.893837	60.2615242
1173	1412.09864	60.2341079
1174	1413.303561	60.2068061
1175	1414.508793	60.1793212
1176	1415.714242	60.1516609
1177	1416.918579	60.1238174
1178	1418.123866	60.0961494
1179	1419.329327	60.0687866
1180	1420.534892	60.0425224
1181	1421.741113	60.0162963
1182	1422.944587	59.9911918
1183	1424.148886	59.9650154
1184	1425.352729	59.9390029
1185	1426.558197	59.9131355
1186	1427.76274	59.8873596
1187	1428.967667	59.8625602
1188	1430.171562	59.837944
1189	1431.376308	59.8139228
1190	1432.581144	59.7886276
1191	1433.78561	59.7632446
1192	1434.990939	59.7369766
1193	1436.194723	59.7113342
1194	1437.399516	59.6856002
1195	1438.604525	59.6599426
1196	1439.809534	59.6348953
1197	1441.014507	59.6089706
1198	1442.222769	59.5826606

1199	1443.42646	59.5566482
1200	1444.630086	59.5305099
1201	1445.834581	59.5052795
1202	1447.038838	59.4805526
1203	1448.243312	59.4554328
1204	1449.447684	59.4303817
1205	1450.651878	59.4051017
1206	1451.856838	59.38031
1207	1453.061207	59.3555412
1208	1454.264768	59.3317184
1209	1455.468926	59.3070907
1210	1456.673197	59.2833023
1211	1457.877446	59.258831
1212	1459.08261	59.2341804
1213	1460.288063	59.2097396
1214	1461.493007	59.1845092
1215	1462.698309	59.1598625
1216	1463.901997	59.1346435
1217	1465.106331	59.1087837
1218	1466.309985	59.0828857
1219	1467.51374	59.0565376
1220	1468.717513	59.0297431
1221	1469.921428	59.0034255
1222	1471.125639	58.9767875
1223	1472.33075	58.95055
1224	1473.536004	58.9258041
1225	1474.74133	58.9008178
1226	1475.946193	58.8757514
1227	1477.150315	58.8514404
1228	1478.354794	58.8270454
1229	1479.558942	58.8037834
1230	1480.764782	58.7809677
1231	1481.969557	58.757698
1232	1483.174061	58.7349624
1233	1484.377968	58.711956
1234	1485.583191	58.6881179
1235	1486.786708	58.6644783
1236	1487.991502	58.640789
1237	1489.197002	58.6172676
1238	1490.401174	58.5934944
1239	1491.605417	58.5695915
1240	1492.810209	58.5456619
1241	1494.015194	58.5211105
1242	1495.219424	58.4964599
1243	1496.424626	58.472393
1244	1497.628898	58.4483947
1245	1498.833834	58.4235954
1246	1500.039312	58.3985786
1247	1501.244369	58.3730468
1248	1502.449279	58.3473739

1249	1503.653394	58.3218269
1250	1504.858108	58.295948
1251	1506.062922	58.2706909
1252	1507.267965	58.2456283
1253	1508.47235	58.2202529
1254	1509.677403	58.1948623
1255	1510.882055	58.1695251
1256	1512.086408	58.1449279
1257	1513.291646	58.1205024
1258	1514.497186	58.0962104
1259	1515.702283	58.0723953
1260	1516.907236	58.048664
1261	1518.11159	58.0246582
1262	1519.316041	58.0011405
1263	1520.520562	57.9771537
1264	1521.725584	57.9537124
1265	1522.93046	57.9301261
1266	1524.134719	57.9063949
1267	1525.339298	57.8829879
1268	1526.545309	57.8602714
1269	1527.750615	57.8361015
1270	1528.95619	57.8120231
1271	1530.160539	57.7891464
1272	1531.364887	57.7656631
1273	1532.570004	57.7417984
1274	1533.77573	57.7183456
1275	1534.981152	57.6953048
1276	1536.185149	57.6731033
1277	1537.38989	57.6495132
1278	1538.593574	57.6258926
1279	1539.797678	57.6028366
1280	1541.002685	57.5796089
1281	1542.208083	57.5554008
1282	1543.413606	57.5320854
1283	1544.617794	57.5091133
1284	1545.822641	57.485485
1285	1547.027818	57.4623641
1286	1548.23245	57.4380111
1287	1549.437234	57.414318
1288	1550.642203	57.3909912
1289	1551.846811	57.3680305
1290	1553.052633	57.3459663
1291	1554.257368	57.3242683
1292	1555.462228	57.3017883
1293	1556.66687	57.2796325
1294	1557.87194	57.2571945
1295	1559.075749	57.2349052
1296	1560.281109	57.2126274
1297	1561.485381	57.1919174
1298	1562.689881	57.1698303

1299	1563.893982	57.1480216
1300	1565.098104	57.1254501
1301	1566.302282	57.1027221
1302	1567.507669	57.080223
1303	1568.712589	57.0580215
1304	1569.91752	57.0358276
1305	1571.122668	57.0140876
1306	1572.327541	56.9923019
1307	1573.532569	56.9695968
1308	1574.738089	56.9474906
1309	1575.942912	56.925373
1310	1577.146964	56.9032974
1311	1578.350371	56.8819465
1312	1579.555444	56.8598556
1313	1580.759721	56.8367919
1314	1581.964965	56.8140563
1315	1583.169617	56.7906684
1316	1584.375448	56.7670669
1317	1585.580441	56.7440795
1318	1586.785166	56.7212219
1319	1587.990399	56.6974372
1320	1589.195047	56.6739387
1321	1590.400174	56.6497726
1322	1591.603824	56.6273727
1323	1592.808104	56.6052055
1324	1594.012535	56.5827331
1325	1595.217723	56.561016
1326	1596.422966	56.539875
1327	1597.627265	56.518238
1328	1598.832151	56.4960327
1329	1600.03759	56.4745178
1330	1601.242774	56.4530715
1331	1602.448111	56.4309806
1332	1603.652139	56.4075317
1333	1604.902695	56.3844299
1334	1606.107125	56.361
1335	1607.312463	56.3365783
1336	1608.517603	56.3121109
1337	1609.722162	56.2877273
1338	1610.927002	56.263771
1339	1612.131412	56.2399673
1340	1613.336114	56.2156791
1341	1614.539785	56.1925735
1342	1615.745559	56.1690292
1343	1616.950527	56.1456909
1344	1618.155549	56.1230964
1345	1619.36026	56.1001586
1346	1620.56475	56.0774421
1347	1621.770216	56.0544624
1348	1622.974851	56.0315856

1349	1624.179664	56.008171
1350	1625.382897	55.9844894
1351	1626.587037	55.9608764
1352	1627.792576	55.9374771
1353	1628.997392	55.9144172
1354	1630.202217	55.8907356
1355	1631.405967	55.8674697
1356	1632.609766	55.8442764
1357	1633.813626	55.8209609
1358	1635.017388	55.7973136
1359	1636.221194	55.7744636
1360	1637.425075	55.7526664
1361	1638.629881	55.7304611
1362	1639.834364	55.7083969
1363	1641.038926	55.6858901
1364	1642.24428	55.6634521
1365	1643.449821	55.6415481
1366	1644.654878	55.6196289
1367	1645.860176	55.5975151
1368	1647.064951	55.5762138
1369	1648.270481	55.5539054
1370	1649.474957	55.5317916
1371	1650.680125	55.5091438
1372	1651.884029	55.4869384
1373	1653.087827	55.4645767
1374	1654.292043	55.4428024
1375	1655.497342	55.4211845
1376	1656.702569	55.3988571
1377	1657.908176	55.3771858
1378	1659.113231	55.3547058
1379	1660.317078	55.3329162
1380	1661.522159	55.3103752
1381	1662.727022	55.2880249
1382	1663.931987	55.2657356
1383	1665.137221	55.2431869
1384	1666.341412	55.2211227
1385	1667.547398	55.1981201
1386	1668.751766	55.1758842
1387	1669.957518	55.1535339
1388	1671.162679	55.1321945
1389	1672.367374	55.1098442
1390	1673.571846	55.0882148
1391	1674.776359	55.0665054
1392	1675.980903	55.0452384
1393	1677.184576	55.0238952
1394	1678.389224	55.0017929
1395	1679.593292	54.9800109
1396	1680.798284	54.9589767
1397	1682.002986	54.9376754
1398	1683.20776	54.915657

1399	1684.412943	54.8946609
1400	1685.620171	54.8727302
1401	1686.825115	54.8511772
1402	1688.028823	54.8290519
1403	1689.233796	54.8073348
1404	1690.438973	54.7851448
1405	1691.643789	54.7637138
1406	1692.849132	54.7415084
1407	1694.053033	54.7189331
1408	1695.257756	54.6963005
1409	1696.462242	54.6741523
1410	1697.666369	54.6520156
1411	1698.872416	54.6297531
1412	1700.076961	54.6074752
1413	1701.281768	54.5857467
1414	1702.486703	54.5648689
1415	1703.692043	54.5430831
1416	1704.898095	54.5216484
1417	1706.101102	54.5002326
1418	1707.305775	54.4786148
1419	1708.510268	54.45755
1420	1709.715209	54.4367294
1421	1710.919533	54.4156723
1422	1712.124415	54.3945617
1423	1713.328578	54.3731193
1424	1714.532626	54.3518981
1425	1715.737258	54.3302993
1426	1716.942157	54.3090324
1427	1718.147475	54.2877311
1428	1719.352235	54.2669906
1429	1720.556407	54.2453842
1430	1721.761196	54.2233047
1431	1722.966451	54.2015266
1432	1724.170907	54.1799812
1433	1725.375554	54.1591072
1434	1726.580289	54.1376762
1435	1727.785127	54.1169052
1436	1728.990405	54.0955314
1437	1730.19523	54.074913
1438	1731.400209	54.0539321
1439	1732.604385	54.0325431
1440	1733.808698	54.0118637
1441	1735.013954	53.990776
1442	1736.219014	53.9695053
1443	1737.423248	53.9477615
1444	1738.628783	53.9261283
1445	1739.833048	53.9046134
1446	1741.037229	53.8830261
1447	1742.241516	53.8608779
1448	1743.447002	53.8391532

1449	1744.65305	53.8171081
1450	1745.857179	53.7950935
1451	1747.06194	53.7740173
1452	1748.266798	53.7527236
1453	1749.472374	53.7314033
1454	1750.677341	53.7101135
1455	1751.883322	53.6888809
1456	1753.088228	53.6673088
1457	1754.292668	53.6465339
1458	1755.497963	53.6252937
1459	1756.70362	53.6051406
1460	1757.909485	53.5842895
1461	1759.114589	53.5631942
1462	1760.318931	53.5419197
1463	1761.523834	53.5204658
1464	1762.72857	53.4992294
1465	1763.933269	53.4783096
1466	1765.138141	53.4575309
1467	1766.342681	53.4364852
1468	1767.546856	53.4155044
1469	1768.752585	53.3940963
1470	1769.957787	53.3736343
1471	1771.163027	53.3524284
1472	1772.368186	53.3325195
1473	1773.572334	53.3126411
1474	1774.777042	53.2923583
1475	1775.981141	53.2714157
1476	1777.185889	53.2507095
1477	1778.391094	53.2294845
1478	1779.595367	53.2084045
1479	1780.799594	53.1873893
1480	1782.004439	53.1658744
1481	1783.209861	53.145317
1482	1784.415127	53.124031
1483	1785.620465	53.1023025
1484	1786.824016	53.0809822
1485	1788.028441	53.060585
1486	1789.233273	53.0393142
1487	1790.437777	53.0187873
1488	1791.642317	52.9979476
1489	1792.846284	52.9772453
1490	1794.050333	52.9565505
1491	1795.254604	52.935234
1492	1796.458993	52.9137763
1493	1797.663857	52.8930435
1494	1798.8687	52.872509
1495	1800.072494	52.8517456
1496	1801.276722	52.8312072
1497	1802.481521	52.8104133
1498	1803.686762	52.790039

1499	1804.8911	52.7692985
1500	1806.096635	52.7484626
1501	1807.301156	52.7280349
1502	1808.505472	52.7077026
1503	1809.71066	52.6870346
1504	1810.915015	52.6657524
1505	1812.120657	52.6439132
1506	1813.326603	52.6230888
1507	1814.531504	52.6023063
1508	1815.737001	52.5814437
1509	1816.941741	52.559822
1510	1818.146882	52.5392265
1511	1819.350667	52.5190849
1512	1820.554813	52.4993972
1513	1821.759556	52.4799118
1514	1822.964916	52.4612312
1515	1824.170285	52.4419326
1516	1825.375572	52.4232864
1517	1826.579574	52.4044876
1518	1827.78432	52.3857536
1519	1828.988815	52.367958
1520	1830.193459	52.3494377
1521	1831.397775	52.3303947
1522	1832.602348	52.310997
1523	1833.806686	52.2912788
1524	1835.010807	52.2707214
1525	1836.214729	52.2516288
1526	1837.419657	52.2313957
1527	1838.625083	52.2115974
1528	1839.829652	52.1922492
1529	1841.033497	52.1728591
1530	1842.23807	52.1535835
1531	1843.442913	52.1336135
1532	1844.647618	52.1138191
1533	1845.852466	52.0941238
1534	1847.05718	52.0753059
1535	1848.261804	52.0552749
1536	1849.467079	52.0361518
1537	1850.671806	52.017395
1538	1851.876307	51.9980087
1539	1853.080446	51.978981
1540	1854.284644	51.9599227
1541	1855.488812	51.9415054
1542	1856.694021	51.9227066
1543	1857.899481	51.9043159
1544	1859.10471	51.885559
1545	1860.309646	51.8672714
1546	1861.514103	51.8485488
1547	1862.719896	51.8287887
1548	1863.924587	51.8093872

1549	1865.129459	51.7895278
1550	1866.334241	51.7695999
1551	1867.537976	51.7500839
1552	1868.743144	51.7307968
1553	1869.948378	51.7113723
1554	1871.152758	51.6918525
1555	1872.35768	51.6720771
1556	1873.561369	51.6519241
1557	1874.765954	51.6325912
1558	1875.972503	51.6124649
1559	1877.177034	51.592163
1560	1878.382169	51.571846
1561	1879.587615	51.5514831
1562	1880.792	51.5305557
1563	1881.996879	51.5100021
1564	1883.201484	51.4898872
1565	1884.40628	51.4708671
1566	1885.611623	51.4524002
1567	1886.8164	51.4331741
1568	1888.020269	51.4147872
1569	1889.225532	51.3970222
1570	1890.431202	51.3787536
1571	1891.635785	51.3600654
1572	1892.839433	51.3432846
1573	1894.044935	51.3254928
1574	1895.248102	51.3073196
1575	1896.453388	51.288536
1576	1897.658982	51.2697029
1577	1898.863983	51.250904
1578	1900.071354	51.2324333
1579	1901.275376	51.2133255
1580	1902.479914	51.1946029
1581	1903.685304	51.175991
1582	1904.889949	51.1564865
1583	1906.095231	51.1380538
1584	1907.299609	51.119194
1585	1908.50305	51.1002349
1586	1909.707901	51.0814743
1587	1910.913075	51.0631179
1588	1912.117408	51.0433845
1589	1913.322508	51.0242233
1590	1914.527752	51.0052261
1591	1915.732795	50.9866561
1592	1916.938072	50.9679145
1593	1918.14303	50.948574
1594	1919.348781	50.9302062
1595	1920.554133	50.9121322
1596	1921.758512	50.8930931
1597	1922.963566	50.8740463
1598	1924.168508	50.8562126

1599	1925.373668	50.8382034
1600	1926.579062	50.8204307
1601	1927.783222	50.8024368
1602	1928.989302	50.7837409
1603	1930.193602	50.7659416
1604	1931.398583	50.746273
1605	1932.603332	50.7266006
1606	1933.807791	50.7072525
1607	1935.012165	50.6880569
1608	1936.216867	50.6690864
1609	1937.420608	50.6496276
1610	1938.624343	50.6298675
1611	1939.82813	50.6106224
1612	1941.032409	50.5912246
1613	1942.236183	50.5712471
1614	1943.440816	50.5526046
1615	1944.645705	50.5339546
1616	1945.850056	50.5155868
1617	1947.054478	50.4969253
1618	1948.259164	50.4777107
1619	1949.462623	50.4593849
1620	1950.667022	50.4415473
1621	1951.871539	50.4231796
1622	1953.076304	50.4054222
1623	1954.279987	50.3869857
1624	1955.484346	50.3685302
1625	1956.688889	50.3500099
1626	1957.893072	50.3320045
1627	1959.098408	50.3140106
1628	1960.303222	50.2955932
1629	1961.508001	50.2767524
1630	1962.713211	50.2577896
1631	1963.91826	50.2384147
1632	1965.123345	50.219532
1633	1966.32878	50.2017288
1634	1967.533104	50.1833305
1635	1968.737599	50.1641654
1636	1969.941969	50.1453056
1637	1971.147611	50.125782
1638	1972.352833	50.1071815
1639	1973.558608	50.0883941
1640	1974.762934	50.0695075
1641	1975.967442	50.0511245
1642	1977.171562	50.0324745
1643	1978.376583	50.0137786
1644	1979.580889	49.9953536
1645	1980.785908	49.9774322
1646	1981.990212	49.9590339
1647	1983.194905	49.9420166
1648	1984.399887	49.9247512

1649	1985.605018	49.9067497
1650	1986.809651	49.8887825
1651	1988.014564	49.8714523
1652	1989.219114	49.8535346
1653	1990.424274	49.8353424
1654	1991.628653	49.8174514
1655	1992.833687	49.7999191
1656	1994.039395	49.7824172
1657	1995.244181	49.7645034
1658	1996.448028	49.7465286
1659	1997.652677	49.7290725
1660	1998.857794	49.7116165
1661	2000.062898	49.6934204
1662	2001.267857	49.6760139
1663	2002.471586	49.6583671
1664	2003.675839	49.6404151
1665	2004.880872	49.622673
1666	2006.086608	49.6048469
1667	2007.291743	49.5868721
1668	2008.496927	49.5688552
1669	2009.701068	49.5512313
1670	2010.906332	49.5333442
1671	2012.111659	49.5153312
1672	2013.317004	49.497013
1673	2014.520914	49.479351
1674	2015.725787	49.4612884
1675	2016.930344	49.4433746
1676	2018.133638	49.4249343
1677	2019.337975	49.4063262
1678	2020.54303	49.3874092
1679	2021.747577	49.3686866
1680	2022.952458	49.3501434
1681	2024.156625	49.3318939
1682	2025.361061	49.3136634
1683	2026.565981	49.2948074
1684	2027.771267	49.277378
1685	2028.975867	49.2585792
1686	2030.180554	49.2406616
1687	2031.385297	49.2226333
1688	2032.589959	49.2054023
1689	2033.795687	49.1871986
1690	2035.00019	49.1694335
1691	2036.204499	49.1515655
1692	2037.408979	49.1340522
1693	2038.614206	49.1165084
1694	2039.819208	49.0982704
1695	2041.024089	49.080574
1696	2042.229417	49.0625762
1697	2043.433934	49.0447235
1698	2044.638044	49.0260543

1699	2045.843536	49.007965
1700	2047.048621	48.990242
1701	2048.25395	48.9722938
1702	2049.458634	48.9550361
1703	2050.663204	48.9374732
1704	2051.867694	48.9197998
1705	2053.071805	48.902626
1706	2054.27741	48.8853683
1707	2055.482722	48.8685722
1708	2056.686521	48.852436
1709	2057.890884	48.8354377
1710	2059.096533	48.8180618
1711	2060.301641	48.7999534
1712	2061.507329	48.7805099
1713	2062.711415	48.7618637
1714	2063.915674	48.7437095
1715	2065.119714	48.7253456
1716	2066.32534	48.7061729
1717	2067.530697	48.6868782
1718	2068.73536	48.6674804
1719	2069.939694	48.6495895
1720	2071.144163	48.6314849
1721	2072.349187	48.6144409
1722	2073.555071	48.5978622
1723	2074.760459	48.5807113
1724	2075.964349	48.5631675
1725	2077.16876	48.5458908
1726	2078.373874	48.528923
1727	2079.578401	48.5116424
1728	2080.783333	48.4946212
1729	2081.987627	48.4765129
1730	2083.19189	48.4580841
1731	2084.396553	48.4391899
1732	2085.601496	48.4203834
1733	2086.807354	48.4023895
1734	2088.011795	48.3849945
1735	2089.215327	48.367115
1736	2090.4198	48.3489074
1737	2091.624491	48.3309173
1738	2092.828101	48.312107
1739	2094.032758	48.2945632
1740	2095.238053	48.2771224
1741	2096.442345	48.2601699
1742	2097.64656	48.2431373
1743	2098.851306	48.2257652
1744	2100.056797	48.2082595
1745	2101.26211	48.1909294
1746	2102.466023	48.1736755
1747	2103.669833	48.1567993
1748	2104.874345	48.1402053

1749	2106.078721	48.1230773
1750	2107.283766	48.1061325
1751	2108.488707	48.0893974
1752	2109.693242	48.0720901
1753	2110.898263	48.0546607
1754	2112.103526	48.0376205
1755	2113.308877	48.0202789
1756	2114.513777	48.0038414
1757	2115.71953	47.9864616
1758	2116.923125	47.9695396
1759	2118.127469	47.95298
1760	2119.332738	47.9363021
1761	2120.537549	47.9196243
1762	2121.742978	47.9029922
1763	2122.947411	47.8868942
1764	2124.151118	47.8701896
1765	2125.356344	47.8534736
1766	2126.561406	47.8368759
1767	2127.766025	47.8202629
1768	2128.971304	47.803852
1769	2130.175861	47.7868118
1770	2131.380035	47.770092
1771	2132.585168	47.7525291
1772	2133.789952	47.7358322
1773	2134.993748	47.719017
1774	2136.198568	47.7025833
1775	2137.404176	47.6865234
1776	2138.607828	47.6699562
1777	2139.812608	47.6540222
1778	2141.017003	47.6371841
1779	2142.222191	47.6205368
1780	2143.427006	47.6042556
1781	2144.631288	47.5885276
1782	2145.836576	47.5725402
1783	2147.041858	47.5557365
1784	2148.246798	47.5386581
1785	2149.451994	47.5213623
1786	2150.656773	47.5047187
1787	2151.861179	47.4877128
1788	2153.06637	47.4713516
1789	2154.271421	47.4550895
1790	2155.476098	47.4383773
1791	2156.68046	47.4222183
1792	2157.885056	47.4054832
1793	2159.090753	47.3901138
1794	2160.2962	47.374401
1795	2161.501343	47.3590621
1796	2162.706101	47.3425979
1797	2163.911709	47.3267288
1798	2165.115621	47.310482

1799	2166.320568	47.2943496
1800	2167.524984	47.2781028
1801	2168.73032	47.2607917
1802	2169.934806	47.2440376
1803	2171.139355	47.2263374
1804	2172.344872	47.2087669
1805	2173.549588	47.1912345
1806	2174.755015	47.1738929
1807	2175.960068	47.1566352
1808	2177.16403	47.1396598
1809	2178.369043	47.1226806
1810	2179.57445	47.1057357
1811	2180.779683	47.0891609
1812	2181.9846	47.0730857
1813	2183.189734	47.0567703
1814	2184.394007	47.0405082
1815	2185.598293	47.0239028
1816	2186.802134	47.0075874
1817	2188.00708	46.9909858
1818	2189.212325	46.9747848
1819	2190.416889	46.957901
1820	2191.620975	46.9409103
1821	2192.825758	46.9244003
1822	2194.030635	46.9070549
1823	2195.235191	46.8894882
1824	2196.440076	46.8722381
1825	2197.64426	46.8555793
1826	2198.848694	46.8384933
1827	2200.053942	46.8211593
1828	2201.259418	46.8032112
1829	2202.464807	46.7863845
1830	2203.669391	46.7692642
1831	2204.874255	46.7519912
1832	2206.079203	46.7352752
1833	2207.284003	46.7185478
1834	2208.489224	46.7016792
1835	2209.694447	46.6846351
1836	2210.898556	46.668209
1837	2212.103512	46.6514663
1838	2213.307837	46.6347351
1839	2214.513017	46.6181716
1840	2215.718134	46.6019554
1841	2216.922403	46.5856094
1842	2218.126598	46.5690193
1843	2219.330907	46.5530052
1844	2220.535672	46.5374679
1845	2221.740403	46.5221138
1846	2222.944227	46.5062599
1847	2224.148528	46.4908714
1848	2225.356512	46.4759674

1849	2226.560878	46.4602622
1850	2227.766413	46.4448623
1851	2228.971726	46.4291343
1852	2230.177249	46.4134292
1853	2231.382257	46.3989486
1854	2232.587216	46.3838424
1855	2233.791815	46.3682441
1856	2234.996983	46.3529968
1857	2236.201273	46.3386154
1858	2237.406144	46.3243408
1859	2238.61052	46.309761
1860	2239.815604	46.2951354
1861	2241.020341	46.280651
1862	2242.224175	46.2657966
1863	2243.42784	46.2500991
1864	2244.631629	46.2341423
1865	2245.835512	46.2181968
1866	2247.03933	46.2025146
1867	2248.243709	46.1862106
1868	2249.448227	46.1691513
1869	2250.652996	46.1523971
1870	2251.85755	46.1354103
1871	2253.062057	46.120018
1872	2254.267392	46.1049804
1873	2255.472838	46.0900344
1874	2256.677332	46.0747756
1875	2257.882846	46.0604133
1876	2259.087605	46.0458793
1877	2260.292532	46.0312347
1878	2261.496856	46.0172386
1879	2262.702013	46.0027847
1880	2263.906494	45.9885826
1881	2265.110917	45.9732627
1882	2266.315708	45.9572219
1883	2267.521481	45.9410476
1884	2268.72555	45.9256362
1885	2269.92953	45.9092597
1886	2271.134845	45.8932991
1887	2272.338915	45.8770675
1888	2273.54361	45.8601799
1889	2274.748394	45.8442955
1890	2275.953729	45.8280868
1891	2277.157901	45.8117752
1892	2278.362463	45.796173
1893	2279.56659	45.7804565
1894	2280.772798	45.7652511
1895	2281.977642	45.7496109
1896	2283.18228	45.7341804
1897	2284.385925	45.7184867
1898	2285.593937	45.7030563

1899	2286.798309	45.6871337
1900	2288.003305	45.6718559
1901	2289.206914	45.6559028
1902	2290.41151	45.6406059
1903	2291.616392	45.6253585
1904	2292.821104	45.6094131
1905	2294.032074	45.59412
1906	2295.236765	45.578083
1907	2296.442583	45.5625686
1908	2297.646854	45.5472831
1909	2298.851199	45.5318984
1910	2300.054538	45.5163307
1911	2301.259196	45.5012588
1912	2302.464251	45.4856033
1913	2303.669428	45.4696083
1914	2304.874657	45.4538803
1915	2306.078505	45.437828
1916	2307.283544	45.4220352
1917	2308.489007	45.4064064
1918	2309.693901	45.39151
1919	2310.899548	45.3763885
1920	2312.1036	45.3607406
1921	2313.308291	45.345787
1922	2314.513595	45.3306045
1923	2315.718845	45.3158416
1924	2316.923673	45.3005599
1925	2318.128747	45.2854652
1926	2319.332617	45.2704238
1927	2320.537457	45.2559585
1928	2321.741756	45.2400283
1929	2322.947139	45.2248802
1930	2324.152024	45.2104415
1931	2325.356147	45.1946487
1932	2326.560361	45.1797599
1933	2327.765084	45.1643943
1934	2328.969129	45.1488571
1935	2330.174319	45.1336593
1936	2331.378999	45.1184463
1937	2332.58364	45.1018371
1938	2333.788876	45.0852317
1939	2334.993764	45.0690536
1940	2336.198675	45.0526504
1941	2337.403161	45.0367622
1942	2338.607139	45.0205039
1943	2339.811821	45.0048713
1944	2341.017354	44.9893417
1945	2342.222319	44.9741592
1946	2343.426898	44.9591712
1947	2344.631767	44.9448318
1948	2345.836285	44.931118

1949	2347.04059	44.9165115
1950	2348.246027	44.9016723
1951	2349.450255	44.8873062
1952	2350.655443	44.873207
1953	2351.860919	44.858612
1954	2353.065429	44.8449211
1955	2354.269523	44.8306083
1956	2355.474573	44.8161697
1957	2356.679457	44.8015441
1958	2357.883078	44.7875175
1959	2359.087523	44.7733612
1960	2360.292897	44.7588577
1961	2361.497661	44.7450027
1962	2362.702464	44.7305526
1963	2363.907238	44.7161483
1964	2365.111707	44.7015228
1965	2366.31654	44.6875076
1966	2367.521806	44.6729545
1967	2368.727828	44.6587753
1968	2369.931886	44.6442031
1969	2371.136788	44.6300582
1970	2372.340647	44.6164016
1971	2373.545407	44.6018829
1972	2374.749252	44.587944
1973	2375.953022	44.5736618
1974	2377.156947	44.5592308
1975	2378.362072	44.5443649
1976	2379.565729	44.5300178
1977	2380.769346	44.5148429
1978	2381.974693	44.499855
1979	2383.178644	44.4852523
1980	2384.384137	44.4698371
1981	2385.589017	44.4548645
1982	2386.797553	44.4397392
1983	2388.002117	44.4254684
1984	2389.20672	44.4110946
1985	2390.410925	44.3961219
1986	2391.615807	44.381668
1987	2392.820097	44.3677062
1988	2394.0249	44.3537597
1989	2395.230064	44.3391532
1990	2396.4353	44.3257827
1991	2397.640145	44.3114814
1992	2398.845484	44.2974395
1993	2400.049761	44.2827529
1994	2401.253836	44.2682533
1995	2402.457799	44.2544555
1996	2403.661947	44.2404136
1997	2404.866609	44.2261466
1998	2406.071402	44.2117233

1999	2407.278661	44.1977577
2000	2408.483904	44.1831588
2001	2409.688598	44.1682968
2002	2410.892887	44.1534118
2003	2412.097834	44.1384811
2004	2413.302271	44.1236648
2005	2414.506295	44.1092338
2006	2415.71133	44.0938873
2007	2416.916639	44.0792045
2008	2418.121386	44.0645637
2009	2419.325764	44.0499191
2010	2420.530203	44.0353317
2011	2421.734213	44.0206947
2012	2422.939121	44.0062599
2013	2424.143949	43.9921073
2014	2425.349349	43.9775085
2015	2426.554409	43.9635086
2016	2427.759181	43.9497795
2017	2428.963748	43.9368476
2018	2430.169041	43.9231071
2019	2431.374187	43.909851
2020	2432.57963	43.8965377
2021	2433.784096	43.8839073
2022	2434.988551	43.8714828
2023	2436.194135	43.8582534
2024	2437.400358	43.8454971
2025	2438.604533	43.8319587
2026	2439.80972	43.818489
2027	2441.01323	43.8044853
2028	2442.217042	43.7902908
2029	2443.420935	43.7761001
2030	2444.625704	43.7619552
2031	2445.830823	43.7480697
2032	2447.034547	43.7335891
2033	2448.23853	43.7195777
2034	2449.443041	43.7056083
2035	2450.648275	43.69141
2036	2451.853377	43.6774749
2037	2453.057889	43.6631202
2038	2454.262547	43.6495628
2039	2455.467386	43.6351318
2040	2456.672348	43.6210784
2041	2457.878041	43.6069526
2042	2459.082674	43.5924873
2043	2460.286107	43.5783271
2044	2461.490142	43.5639877
2045	2462.695543	43.5491752
2046	2463.90077	43.5345191
2047	2465.105471	43.519638
2048	2466.309655	43.5048332

2049	2467.514716	43.4904708
2050	2468.719038	43.4754867
2051	2469.924135	43.4598922
2052	2471.128976	43.4448776
2053	2472.333982	43.4290924
2054	2473.53847	43.413475
2055	2474.742812	43.3974418
2056	2475.949051	43.381874
2057	2477.15397	43.3660354
2058	2478.358616	43.3505401
2059	2479.563495	43.3350105
2060	2480.767774	43.3200569
2061	2481.972808	43.3060379
2062	2483.177992	43.2915344
2063	2484.381861	43.2782211
2064	2485.587763	43.2638664
2065	2486.791594	43.2508163
2066	2487.996094	43.237091
2067	2489.200112	43.2244606
2068	2490.405411	43.2110328
2069	2491.610336	43.1978759
2070	2492.815335	43.1843185
2071	2494.019739	43.1707229
2072	2495.224419	43.1573867
2073	2496.429698	43.1438903
2074	2497.634746	43.1307792
2075	2498.839189	43.1176071
2076	2500.043957	43.104309
2077	2501.247897	43.0903205
2078	2502.452705	43.0770187
2079	2503.658186	43.0634689
2080	2504.862955	43.0493698
2081	2506.067452	43.0355911
2082	2507.270993	43.0216369
2083	2508.475924	43.0070762
2084	2509.680401	42.9938392
2085	2510.885731	42.9795532
2086	2512.090987	42.9653968
2087	2513.295559	42.9517707
2088	2514.499521	42.9379539
2089	2515.704475	42.9239578
2090	2516.908607	42.9105529
2091	2518.113718	42.8970642
2092	2519.318754	42.8834495
2093	2520.523375	42.8692092
2094	2521.72799	42.8544235
2095	2522.932653	42.8407325
2096	2524.137595	42.8267745
2097	2525.34284	42.8129997
2098	2526.548063	42.7988395

2099	2527.753011	42.7844696
2100	2528.95748	42.770153
2101	2530.162283	42.7558631
2102	2531.367414	42.7415695
2103	2532.572238	42.72826
2104	2533.776656	42.7145996
2105	2534.980601	42.7009506
2106	2536.186533	42.6870193
2107	2537.391457	42.6731376
2108	2538.595949	42.6593551
2109	2539.800722	42.6465606
2110	2541.00484	42.6334762
2111	2542.208856	42.6204452
2112	2543.412932	42.6068458
2113	2544.618055	42.5938529
2114	2545.823109	42.5810661
2115	2547.02745	42.5679626
2116	2548.231228	42.5547637
2117	2549.435102	42.5416984
2118	2550.638852	42.5286941
2119	2551.842642	42.5150146
2120	2553.047067	42.5014648
2121	2554.260919	42.4870567
2122	2555.464951	42.473793
2123	2556.669218	42.4600486
2124	2557.873881	42.4461708
2125	2559.077846	42.4324951
2126	2560.28367	42.4194259
2127	2561.488079	42.4060287
2128	2562.692481	42.3925819
2129	2563.897163	42.3792037
2130	2565.101418	42.3656501
2131	2566.305753	42.3525962
2132	2567.510108	42.3389587
2133	2568.714694	42.325283
2134	2569.920181	42.3113555
2135	2571.124311	42.2977485
2136	2572.329365	42.2844772
2137	2573.534513	42.2709999
2138	2574.738273	42.2578849
2139	2575.943316	42.2445755
2140	2577.148357	42.2323493
2141	2578.35363	42.2198448
2142	2579.558667	42.2069892
2143	2580.764031	42.1952629
2144	2581.967865	42.1828536
2145	2583.171881	42.1711807
2146	2584.3777	42.1587867
2147	2585.582666	42.1463165
2148	2586.787444	42.1339035

2149	2587.992413	42.121437
2150	2589.196679	42.109024
2151	2590.402052	42.0966758
2152	2591.606955	42.085781
2153	2592.812282	42.0728988
2154	2594.017469	42.0615959
2155	2595.22116	42.0489082
2156	2596.425601	42.0370178
2157	2597.631567	42.0251312
2158	2598.835732	42.0136222
2159	2600.04075	42.0016708
2160	2601.245903	41.9895057
2161	2602.450797	41.9771156
2162	2603.655581	41.964344
2163	2604.860808	41.9514808
2164	2606.066063	41.9384078
2165	2607.271207	41.9253158
2166	2608.570933	41.9123535
2167	2609.775745	41.8993911
2168	2610.980952	41.8856849
2169	2612.186413	41.8727035
2170	2613.391713	41.8586692
2171	2614.595974	41.8453826
2172	2615.800556	41.8321189
2173	2617.004519	41.8194999
2174	2618.209644	41.8065376
2175	2619.415196	41.7935409
2176	2620.619653	41.7803993
2177	2621.824482	41.7669029
2178	2623.029123	41.7533111
2179	2624.234193	41.7398376
2180	2625.439585	41.7268829
2181	2626.644468	41.7142791
2182	2627.848722	41.70055
2183	2629.052782	41.6870574
2184	2630.257468	41.6739234
2185	2631.462476	41.6604156
2186	2632.694343	41.6473579
2187	2633.898234	41.6342048
2188	2635.156996	41.622425
2189	2636.361626	41.6110115
2190	2637.565202	41.598854
2191	2638.77068	41.5863914
2192	2639.975859	41.5739746
2193	2641.180521	41.5619735
2194	2642.384806	41.5487136
2195	2643.607049	41.5364379
2196	2644.812268	41.5231094
2197	2646.018062	41.510498
2198	2647.221875	41.4964752

2199	2648.428787	41.4822807
2200	2649.633219	41.4691734
2201	2650.838417	41.4548301
2202	2652.044403	41.4415779
2203	2653.24923	41.4273986
2204	2654.468542	41.4140243
2205	2655.673934	41.4009132
2206	2656.877716	41.3874282
2207	2658.082592	41.3734664
2208	2659.287794	41.3596649
2209	2660.491569	41.3462524
2210	2661.695701	41.3321952
2211	2662.900085	41.3193893
2212	2664.104805	41.3057365
2213	2665.327106	41.2920799
2214	2666.532183	41.2788925
2215	2667.736379	41.2651443
2216	2668.940337	41.2517013
2217	2670.145441	41.2390403
2218	2671.350612	41.2263679
2219	2672.556088	41.2135086
2220	2673.760576	41.2001686
2221	2674.965705	41.187561
2222	2676.186403	41.1746826
2223	2677.391563	41.1623191
2224	2678.595994	41.150238
2225	2679.800895	41.1380805
2226	2681.00553	41.1259689
2227	2682.209597	41.113552
2228	2683.414103	41.1015396
2229	2684.619332	41.0887336
2230	2685.82434	41.0770263
2231	2687.044905	41.0632553
2232	2688.248331	41.0508193
2233	2689.453106	41.0383491
2234	2690.657286	41.0248069
2235	2691.86182	41.0116348
2236	2693.067691	40.9984817
2237	2694.272871	40.985897
2238	2695.476998	40.9730453
2239	2696.681852	40.9609756
2240	2697.904213	40.9488716
2241	2699.108748	40.9377517
2242	2700.3127	40.926239
2243	2701.516873	40.9146652
2244	2702.7218	40.903656
2245	2703.926298	40.8923454
2246	2705.131454	40.8807601
2247	2706.336498	40.8686141
2248	2707.541377	40.8563194

2249	2708.761318	40.8440704
2250	2709.966416	40.8315048
2251	2711.171658	40.8191299
2252	2712.376839	40.8064193
2253	2713.582159	40.7937355
2254	2714.786601	40.7816314
2255	2715.990585	40.7691001
2256	2717.195766	40.7567749
2257	2718.400783	40.7440757
2258	2719.620551	40.7323303
2259	2720.824189	40.7204017
2260	2722.028884	40.7085685
2261	2723.23389	40.6965446
2262	2724.439349	40.6838912
2263	2725.645012	40.6716461
2264	2726.850699	40.6592483
2265	2728.054577	40.6467514
2266	2729.258774	40.6348762
2267	2730.481278	40.6230545
2268	2731.685802	40.6101989
2269	2732.89168	40.5967788
2270	2734.096616	40.5835151
2271	2735.301492	40.5699615
2272	2736.505421	40.5572547
2273	2737.710697	40.5438957
2274	2738.915979	40.5304794
2275	2740.120417	40.5178375
