# Supplementary material for: An Ultra-Compact and Low-Cost LAMP-Based Virus Detection Device
Source: Sensors (Basel). 2024 Jul 29;24(15):4912. doi: 10.3390/s24154912 (PMC11314854; doi:10.3390/s24154912)
Supplement: Supplementary file 1 [file sensors-24-04912-s001.zip › One time release & PCM .pdf]

Number	Time (s)	Temperature (° C)
1	0.0005859	21.9140892
2	1.204841	21.9348411
3	2.4080988	21.9476451
4	3.6127233	21.9701995
5	4.8255997	22.8409461
6	6.0303769	23.5490589
7	7.2345138	23.9973926
8	8.4378808	24.2742424
9	9.6417492	24.4445724
10	10.8459273	24.5278644
11	12.050454	24.7888278
12	13.3008638	25.3387279
13	14.5052812	25.8594017
14	15.7097804	26.3537044
15	16.9143503	26.4327945
16	18.1192369	26.5427646
17	19.3237913	26.5199031
18	20.5285031	26.3720188
19	21.7325745	26.090969
20	22.9378559	25.7959442
21	24.1431194	25.5158729
22	25.3484465	24.9546718
23	26.5525402	24.5494956
24	27.7576553	24.5824222
25	28.9628201	24.4304161
26	30.1677958	23.9565238
27	31.3715418	23.6798553
28	32.5767793	23.4399089
29	33.7823624	23.387186
30	34.9860491	23.4130649
31	36.1909678	23.5248718
32	37.3956754	23.671297
33	38.6001105	23.7266387
34	39.8047443	23.5339469
35	41.0086201	23.3315906
36	42.2126103	23.407936
37	43.4176116	23.3766212
38	44.6227167	23.4499015
39	45.8269841	23.5434284
40	47.0316612	23.9888191
41	48.2366123	24.22583
42	49.441053	24.3703422
43	50.6457068	24.3826923
44	51.849344	24.4411716
45	53.0529347	24.4714145
46	54.2576108	24.5316238
47	55.4617301	24.6305904
48	56.6656134	24.7619533
49	57.8693679	24.855585

50	59.0729667	24.5896854
51	60.2768586	24.5034866
52	61.4805854	24.4580688
53	62.6850188	24.4997615
54	63.8898225	24.321022
55	65.0954015	24.3524131
56	66.3003525	24.257307
57	67.5047674	24.1648483
58	68.7096718	24.0633792
59	69.9130404	23.9660758
60	71.1180573	23.8649063
61	72.3215665	23.7697792
62	73.5258322	23.6866416
63	74.7288859	23.609848
64	75.9331982	23.5357971
65	77.1383753	23.4588546
66	78.342322	23.4170112
67	79.5466598	23.4154415
68	80.7514448	23.4290313
69	81.9565539	23.4446983
70	83.1613223	23.4462432
71	84.3658339	23.4588394
72	85.5722893	23.4627952
73	86.7771907	23.4692325
74	87.9815772	23.4704933
75	89.1849117	23.470993
76	90.3897267	23.4659595
77	91.5942862	23.4638252
78	92.7987524	23.4497108
79	94.003658	23.4302368
80	95.2081635	23.4214878
81	96.4118571	23.3951053
82	97.6161607	23.3670406
83	98.8203272	23.336111
84	100.0249769	23.3076839
85	101.2302083	23.2789573
86	102.4337554	23.2503795
87	103.6381803	23.2176322
88	104.8427627	23.188694
89	106.0460497	23.1610832
90	107.2615052	23.1335563
91	108.4662098	23.1069259
92	109.6708036	23.0809669
93	110.8758941	23.0545024
94	112.0796986	23.0299644
95	113.2835382	23.0060615
96	114.5028638	22.9837799
97	115.7067602	22.96352
98	116.9116437	22.9399337
99	118.1168819	22.9165

100	119.3248155	22.8950099
101	120.5293532	22.8742465
102	121.7340052	22.8544578
103	122.9379903	22.8352851
104	124.1430475	22.8167896
105	125.3475572	22.7995967
106	126.5526416	22.782917
107	127.7566894	22.7672386
108	128.9596122	22.753952
109	130.1643572	22.7416343
110	131.3695597	22.7294101
111	132.5735147	22.7182884
112	133.7776683	22.7076435
113	134.9823903	22.6979866
114	136.1863105	22.6887302
115	137.3903894	22.6811237
116	138.5952129	22.6739845
117	139.7993066	22.6652812
118	141.0036981	22.6591873
119	142.2086784	22.6527404
120	143.4125151	22.6465797
121	144.6168916	22.64188
122	145.8210014	22.6376209
123	147.0257504	22.6338481
124	148.2301396	22.6314182
125	149.4335488	22.628334
126	150.6379149	22.6263751
127	151.8421324	22.624895
128	153.0462945	22.6237564
129	154.2513327	22.6237888
130	155.4555831	22.6250648
131	156.6594921	22.6269073
132	157.8643392	22.6305732
133	159.0689857	22.6348476
134	160.2730836	22.6395378
135	161.4775171	22.6460914
136	162.6817352	22.652502
137	163.8857301	22.6611747
138	165.0898809	22.6692447
139	166.2944607	22.6789894
140	167.498882	22.6908493
141	168.7029075	22.7023811
142	169.9086787	22.7142086
143	171.1138544	22.7278957
144	172.3180248	22.7423191
145	173.5220856	22.7573413
146	174.7262891	22.7741127
147	175.9303834	22.7924919
148	177.1346128	22.8127193
149	178.3390874	22.8333759

150	179.5434805	22.8546466
151	180.7477081	22.8771553
152	181.9516555	22.9015254
153	183.1571262	22.925394
154	184.3626054	22.9502563
155	185.5664965	22.9746818
156	186.7704774	22.9992141
157	187.9754988	23.0233879
158	189.1813017	23.0478782
159	190.3844285	23.073246
160	191.5887417	23.0978584
161	192.7931986	23.1232357
162	193.9978373	23.1490135
163	195.2020173	23.1743659
164	196.4067389	23.2000465
165	197.6116159	23.2263736
166	198.8163651	23.2533226
167	200.0203457	23.2813625
168	201.2254967	23.3105125
169	202.4302004	23.340229
170	203.663711	23.3734798
171	204.8674492	23.4101276
172	206.1235674	23.447977
173	207.328221	23.4898891
174	208.5321103	23.5349979
175	209.7357222	23.5835685
176	210.9395759	23.6342468
177	212.1433607	23.6885948
178	213.3471408	23.7438392
179	214.5696497	23.8036384
180	215.7747055	23.8665885
181	216.9995254	23.9333419
182	218.203769	24.0060195
183	219.4140911	24.0838394
184	220.618645	24.1659641
185	221.822564	24.2543869
186	223.028173	24.3488426
187	224.2327179	24.4487094
188	225.4381567	24.554531
189	226.6429162	24.6650829
190	227.8897251	24.7809238
191	229.094729	24.9002571
192	230.2987437	25.0204448
193	231.504004	25.1426734
194	232.7085244	25.2690906
195	233.9116742	25.4006004
196	235.1164612	25.5354423
197	236.319381	25.6779689
198	237.5242548	25.8281974
199	238.7471033	25.9904537

200	239.9511354	26.1600284
201	241.1562791	26.332983
202	242.360863	26.507761
203	243.5652391	26.6884498
204	244.7699135	26.8724422
205	245.9747362	27.0577239
206	247.1793157	27.252655
207	248.3836316	27.4534358
208	249.6047857	27.6577606
209	250.8086141	27.9933872
210	252.0127498	28.2945442
211	253.2162304	28.6075592
212	254.421706	29.0296573
213	255.6259339	29.4704017
214	256.8296576	29.8750305
215	258.0343947	30.3370113
216	259.2384521	30.8225708
217	260.4600569	31.2515563
218	261.6644443	31.668682
219	262.8684548	31.9328002
220	264.0733658	32.2309837
221	265.2778549	32.5067062
222	266.4824403	32.6680259
223	267.6874833	33.1988029
224	268.8914367	33.6137084
225	270.0946614	33.9991683
226	271.3172878	34.3498306
227	272.5202442	34.7383041
228	273.7236686	35.0951614
229	274.9288903	35.4513092
230	276.1330843	35.7929611
231	277.3365524	36.1585807
232	278.5409356	36.5639457
233	279.7445543	36.5625648
234	280.9486253	36.7338943
235	282.1708524	36.9033584
236	283.3749223	37.1169738
237	284.5784476	37.361061
238	285.7831297	37.6417961
239	286.9868405	37.9636726
240	288.1908493	38.3276367
241	289.3954815	38.7042236
242	290.6004347	39.0606689
243	291.8045454	39.4546737
244	293.0254687	39.8404808
245	294.2311117	40.2094345
246	295.4366803	40.5475845
247	296.6399989	40.8885688
248	297.8440007	41.242897
249	299.0486343	41.5973281

250	300.2537592	41.9460067
251	301.4589893	42.2860679
252	302.6620794	42.6492958
253	303.8835954	43.018505
254	305.0879273	43.3931083
255	306.2926362	43.7752647
256	307.4971037	44.165184
257	308.7025741	44.5400199
258	309.9068272	44.921463
259	311.1103964	45.287075
260	312.3144976	45.6421508
261	313.5188665	45.9972114
262	314.740512	46.3404731
263	315.9446737	46.6749954
264	317.1487345	47.0324707
265	318.3528545	47.3935852
266	319.5570605	47.751934
267	320.761185	48.1316833
268	321.9655348	48.5063819
269	323.169723	48.8816452
270	324.3739094	49.2543029
271	325.5960888	49.6305847
272	326.8006994	50.0062332
273	328.0053245	50.3707733
274	329.2102862	50.7049484
275	330.4141991	51.0194587
276	331.617894	51.3311843
277	332.821962	51.6307907
278	334.0255858	51.9287605
279	335.2294027	52.2205429
280	336.4509589	52.4984626
281	337.6541955	52.7564888
282	338.8588803	53.0008163
283	340.0634485	53.2420463
284	341.2679863	53.4787597
285	342.472923	53.724121
286	343.6764071	53.9669075
287	344.8801725	54.196289
288	346.0853786	54.4229431
289	347.3071012	54.6440544
290	348.512053	54.8692855
291	349.716619	55.0967025
292	350.9204465	55.3162155
293	352.1251671	55.5346603
294	353.3302867	55.750122
295	354.5351097	55.9588623
296	355.7398969	56.1637039
297	356.9606891	56.3686752
298	358.2295771	56.568634
299	359.4338171	56.7756729

300	360.6386088	56.9835319
301	361.8432719	57.1883316
302	363.0477331	57.3948249
303	364.2517232	57.6001396
304	365.4561092	57.8054809
305	366.6608928	58.0097122
306	367.8657004	58.2131347
307	369.0707268	58.4131851
308	370.2753252	58.610218
309	371.4791954	58.8016853
310	372.6843358	58.990242
311	373.8899395	59.1792182
312	375.0946579	59.3675041
313	376.2992588	59.5562973
314	377.5034567	59.744049
315	378.7072886	59.9277153
316	379.912201	60.1039962
317	381.1168894	60.2755622
318	382.3213164	60.4449272
319	383.5549356	60.6101188
320	384.7595448	60.7726516
321	385.9636	60.9329299
322	387.1682149	61.0885505
323	388.3732515	61.2366714
324	389.5779725	61.3749198
325	390.7819661	61.5080184
326	391.9862393	61.6376876
327	393.1905787	61.765026
328	394.3944514	61.8887138
329	395.5993325	62.0090332
330	396.8038128	62.1237983
331	398.0078461	62.2340621
332	399.2120093	62.3418083
333	400.4158213	62.45055
334	401.6202396	62.5601577
335	402.8241269	62.6703987
336	404.0408461	62.7814254
337	405.2451307	62.8920288
338	406.4496075	63.0023612
339	407.654905	63.1128768
340	408.8592148	63.2234878
341	410.0636613	63.3333358
342	411.2678018	63.4420661
343	412.4732658	63.5484695
344	413.6779009	63.6539192
345	414.8834175	63.7584457
346	416.0875865	63.8617057
347	417.2925714	63.9646987
348	418.4965479	64.0658111
349	419.7014327	64.1656875

350	420.9066389	64.2655181
351	422.1116055	64.3650894
352	423.3166188	64.4651336
353	424.5201957	64.5642852
354	425.7240955	64.6625137
355	426.9284033	64.7603912
356	428.133906	64.8587036
357	429.3384926	64.9585723
358	430.543081	65.0581207
359	431.7476146	65.1576766
360	432.9530618	65.2573165
361	434.1575437	65.3576889
362	435.3625467	65.4584808
363	436.5675205	65.5587463
364	437.7723151	65.6590805
365	438.977058	65.759262
366	440.1824793	65.8567657
367	441.3872787	65.9518585
368	442.5921419	66.0471496
369	443.7970248	66.1431427
370	445.0007769	66.2373657
371	446.2056404	66.3295135
372	447.410556	66.4200286
373	448.6153732	66.5105438
374	449.8195117	66.6002349
375	451.0238824	66.6887359
376	452.2281378	66.7775726
377	453.4326337	66.8647308
378	454.6369342	66.9511718
379	455.8411703	67.0358886
380	457.0462575	67.1218872
381	458.250032	67.2094879
382	459.4547596	67.2968063
383	460.6592653	67.3842849
384	461.8638342	67.4699249
385	463.0684837	67.5526123
386	464.2731597	67.6325454
387	465.4774091	67.7097244
388	466.6816745	67.7829437
389	467.8855805	67.8572082
390	469.0897264	67.9290771
391	470.2951067	67.9954681
392	471.4990565	68.0577926
393	472.7040133	68.1170806
394	473.9086699	68.1740264
395	475.1139884	68.2294464
396	476.3177338	68.2822189
397	477.5229175	68.3328018
398	478.7275018	68.3819732
399	479.9318674	68.42527

400	481.1366857	68.4657745
401	482.3417454	68.5112075
402	483.5461464	68.5613632
403	484.7507975	68.6140594
404	485.954874	68.6706314
405	487.1594707	68.732933
406	488.3640839	68.799118
407	489.5689935	68.8687591
408	490.7724173	68.9419708
409	491.9769542	69.0163421
410	493.181275	69.0903778
411	494.385264	69.1622467
412	495.590602	69.2291717
413	496.7959382	69.2891845
414	498.0008565	69.3442459
415	499.2051202	69.3908996
416	500.4094225	69.4321289
417	501.6143241	69.4688949
418	502.8193696	69.5006484
419	504.0236381	69.5291595
420	505.2283368	69.5540237
421	506.4330521	69.5737457
422	507.6374321	69.5908355
423	508.8423078	69.606903
424	510.0466909	69.6220855
425	511.2510434	69.6386413
426	512.4548969	69.6553192
427	513.6588988	69.6703567
428	514.8626612	69.6884384
429	516.0665346	69.7077636
430	517.2701291	69.7291564
431	518.4741259	69.74971
432	519.677826	69.7701263
433	520.8812627	69.7931213
434	522.0853153	69.8153762
435	523.2902359	69.8365173
436	524.4946529	69.8571777
437	525.6986751	69.8789215
438	526.9030683	69.8977508
439	528.1075991	69.9153137
440	529.3124448	69.9328765
441	530.5176755	69.9502563
442	531.7215304	69.9672622
443	532.9247252	69.9815673
444	534.12907	69.9966888
445	535.3331814	70.0094146
446	536.5376791	70.0219879
447	537.7408631	70.033905
448	538.945281	70.0444946
449	540.1499246	70.0532913

450	541.3542403	70.0605239
451	542.5594546	70.0670776
452	543.7644695	70.0730209
453	544.9689971	70.0796203
454	546.1728363	70.0847015
455	547.3769281	70.090599
456	548.5813363	70.0965347
457	549.7862936	70.1023406
458	550.9914832	70.108551
459	552.194936	70.1157073
460	553.3994757	70.1223144
461	554.6037883	70.1278533
462	555.808846	70.1327133
463	557.0137953	70.1371078
464	558.2183023	70.1417083
465	559.4225586	70.1460571
466	560.626794	70.1506271
467	561.8313508	70.1549224
468	563.0362901	70.1588668
469	564.2411955	70.1628265
470	565.4462437	70.1672592
471	566.6496771	70.171791
472	567.8542619	70.177597
473	569.0589087	70.1830139
474	570.262735	70.1879348
475	571.4676667	70.1931228
476	572.6717977	70.1979293
477	573.8759554	70.2036361
478	575.0805563	70.2084197
479	576.2846172	70.2130355
480	577.4899704	70.216957
481	578.6948469	70.2229385
482	579.8996291	70.2312316
483	581.1038847	70.2432937
484	582.3082362	70.258934
485	583.5113694	70.2775268
486	584.7156402	70.3002014
487	585.9203161	70.326477
488	587.1245077	70.356491
489	588.3291855	70.3890533
490	589.5339989	70.4238815
491	590.7379016	70.4606094
492	591.9432386	70.4968643
493	593.1479791	70.5305557
494	594.3526625	70.5628128
495	595.5575624	70.5928192
496	596.7623188	70.6200408
497	597.9666635	70.6440353
498	599.179333	70.664978
499	600.3832005	70.6838378

500	601.5877603	70.7011947
501	602.7925379	70.7165145
502	603.9973073	70.7282485
503	605.2023599	70.7390594
504	606.4066483	70.7465744
505	607.6107557	70.7519989
506	608.8160263	70.7541351
507	610.0215547	70.7545928
508	611.2266228	70.7531127
509	612.4310239	70.7476806
510	613.6351344	70.7399063
511	614.8388298	70.728012
512	616.0437344	70.7160415
513	617.2485763	70.7093811
514	618.4533523	70.7082138
515	619.6578862	70.7114944
516	620.8612447	70.7180938
517	622.0661166	70.7259445
518	623.2708733	70.7358169
519	624.475069	70.7477951
520	625.6797525	70.7604598
521	626.8839256	70.7729568
522	628.0885694	70.7836456
523	629.2929786	70.785057
524	630.497267	70.7797393
525	631.7023415	70.768547
526	632.9068342	70.7528457
527	634.1100161	70.7324447
528	635.314296	70.7082901
529	636.5193806	70.6811981
530	637.724754	70.6510314
531	638.9294154	70.6218719
532	640.1345367	70.5936126
533	641.3385289	70.5685195
534	642.5435424	70.5429153
535	643.746963	70.5183181
536	644.9517722	70.493515
537	646.1567617	70.4702606
538	647.3605944	70.4473037
539	648.5656048	70.4244995
540	649.7714364	70.4032058
541	650.9754944	70.3813247
542	652.1795194	70.3587493
543	653.3834051	70.3337554
544	654.5880305	70.3092346
545	655.7912227	70.2844085
546	656.9953081	70.2601852
547	658.2005366	70.2362747
548	659.4049784	70.213211
549	660.6087668	70.1920471

550	661.8156748	70.1708602
551	663.0204161	70.1504364
552	664.2252316	70.1297607
553	665.4303676	70.1092453
554	666.6351309	70.0890045
555	667.8384738	70.0688705
556	669.0429716	70.0486602
557	670.2488756	70.0278015
558	671.4539774	70.0064849
559	672.6587188	69.9846649
560	673.8629793	69.9627761
561	675.0674203	69.9404602
562	676.2722385	69.9186019
563	677.4771361	69.8971328
564	678.6823499	69.8753585
565	679.8879108	69.8526153
566	681.0922043	69.8305206
567	682.2967325	69.8075408
568	683.5010048	69.7844924
569	684.7061598	69.7606201
570	685.9113089	69.737442
571	687.1156137	69.7146606
572	688.3198637	69.6911468
573	689.5239951	69.6684188
574	690.7287828	69.6459503
575	691.9343255	69.6273727
576	693.1391211	69.6098403
577	694.3435868	69.5959243
578	695.5481389	69.5842437
579	696.7524898	69.5739364
580	697.9572731	69.5635452
581	699.1621836	69.5529785
582	700.3670432	69.5438003
583	701.5720497	69.5335845
584	702.7763542	69.5234069
585	703.981524	69.5090713
586	705.1855885	69.4931182
587	706.390434	69.4751434
588	707.5943687	69.4546966
589	708.799327	69.4331741
590	710.0039136	69.4113693
591	711.2081294	69.389595
592	712.4137016	69.3680267
593	713.6180725	69.3469772
594	714.8221212	69.3265991
595	716.0267745	69.306343
596	717.232437	69.2855987
597	718.4370457	69.26445
598	719.6415336	69.2444
599	720.8448915	69.2253036

600	722.0487227	69.2072525
601	723.2526339	69.1893692
602	724.4566019	69.1708831
603	725.6610344	69.1520233
604	726.8659879	69.1316986
605	728.0702886	69.1117782
606	729.2745944	69.1060028
607	730.4794239	69.1050262
608	731.6842836	69.1066055
609	732.8894004	69.1096115
610	734.0935541	69.114006
611	735.2977405	69.1203536
612	736.5024911	69.1272811
613	737.707748	69.1348876
614	738.9120642	69.1425247
615	740.1159149	69.149002
616	741.3191606	69.1399078
617	742.5244488	69.1244735
618	743.7293183	69.1042404
619	744.9343761	69.0789718
620	746.139159	69.0495452
621	747.3438594	69.0158081
622	748.5484118	68.9799346
623	749.7532168	68.9415664
624	750.9587175	68.9029541
625	752.1635684	68.864212
626	753.3682768	68.8255767
627	754.5728081	68.7879714
628	755.7776313	68.7498931
629	756.9816365	68.7135772
630	758.1867308	68.6783599
631	759.3915307	68.6443099
632	760.5953219	68.6093292
633	761.8000495	68.575325
634	763.0044997	68.5410919
635	764.2095769	68.5076293
636	765.4144205	68.4748382
637	766.6187809	68.4421539
638	767.8231113	68.4111175
639	769.0269014	68.3825225
640	770.2316883	68.3525314
641	771.4372707	68.3227157
642	772.6422591	68.2944793
643	773.8468969	68.2665634
644	775.0512807	68.2388153
645	776.2564265	68.2114334
646	777.4609429	68.1844787
647	778.6647945	68.1570816
648	779.8695371	68.129776
649	781.073765	68.1000976

650	782.2782683	68.0719299
651	783.4884496	68.0438842
652	784.6937317	68.0160827
653	785.8988169	67.9887313
654	787.1044375	67.961235
655	788.3081255	67.933525
656	789.5123029	67.9051742
657	790.7173094	67.8775405
658	791.9230066	67.8493347
659	793.1275669	67.8212356
660	794.3322284	67.7933502
661	795.5363312	67.764656
662	796.7404173	67.736206
663	797.944773	67.7075881
664	799.1491795	67.6796722
665	800.3542102	67.6510925
666	801.558287	67.6222991
667	802.7625798	67.5938262
668	803.9674258	67.565361
669	805.173068	67.5377655
670	806.3775993	67.5094757
671	807.5820116	67.4817657
672	808.7857578	67.4537811
673	809.990686	67.4251556
674	811.1951326	67.3970413
675	812.4008642	67.3696746
676	813.6055246	67.3426818
677	814.8092839	67.3152008
678	816.0136612	67.2869186
679	817.2184924	67.2579498
680	818.4236637	67.2292633
681	819.6274962	67.2004928
682	820.8312113	67.1714401
683	822.0351498	67.1423187
684	823.2390433	67.1124725
685	824.4426501	67.0813217
686	825.6470314	67.0507202
687	826.8523277	67.0279693
688	828.0565045	67.0115966
689	829.2613356	66.9952621
690	830.4663266	66.9787292
691	831.6716791	66.9604034
692	832.876249	66.9412384
693	834.0813352	66.9229278
694	835.2855551	66.9055404
695	836.4901963	66.8894805
696	837.6949845	66.8736724
697	838.8999482	66.8500366
698	840.1040291	66.8202209
699	841.3084207	66.7905349

700	842.5123806	66.7607116
701	843.7171902	66.7320098
702	844.921794	66.704483
703	846.1266914	66.6756134
704	847.3312765	66.6463317
705	848.536081	66.6169281
706	849.7400982	66.5858993
707	850.9447353	66.5548095
708	852.1488898	66.5241928
709	853.3533669	66.493225
710	854.557199	66.4621429
711	855.7610612	66.4310226
712	856.9657107	66.3998794
713	858.1704827	66.3696136
714	859.3739467	66.3387832
715	860.5778058	66.3075485
716	861.7815524	66.277275
717	862.9855512	66.2471694
718	864.1896423	66.2170562
719	865.3944905	66.1874008
720	866.5988501	66.1586456
721	867.8037257	66.1308135
722	869.0085526	66.1024322
723	870.2124174	66.0738983
724	871.4175447	66.0447082
725	872.6225528	66.0160446
726	873.8268036	65.9865112
727	875.0311504	65.9553451
728	876.2350237	65.9240264
729	877.4394637	65.8922348
730	878.6440996	65.8586502
731	879.8482822	65.8247375
732	881.0535353	65.7909698
733	882.2581345	65.757431
734	883.4627926	65.7237777
735	884.6674121	65.6906127
736	885.8728918	65.6590042
737	887.0767882	65.6306304
738	888.2806215	65.6047668
739	889.4842068	65.5785293
740	890.6885616	65.5543823
741	891.8927974	65.5300521
742	893.0970948	65.5052871
743	894.30227	65.4799575
744	895.5073854	65.4548187
745	896.7112159	65.4283294
746	897.9154358	65.401535
747	899.1205664	65.3707733
748	900.3257112	65.3379669
749	901.5306942	65.3042373

750	902.7347582	65.269783
751	903.9394009	65.234497
752	905.1440697	65.2003631
753	906.3492274	65.1662979
754	907.5540105	65.1320571
755	908.7581606	65.0982437
756	909.9627645	65.0642318
757	911.1671524	65.0309295
758	912.3715809	64.997116
759	913.576169	64.9649887
760	914.7816314	64.932106
761	915.9859878	64.9004669
762	917.190525	64.8677825
763	918.394798	64.8358993
764	919.6000224	64.8042831
765	920.8047626	64.7727203
766	922.0092564	64.7405853
767	923.2133134	64.7091293
768	924.417708	64.6780776
769	925.6222682	64.646141
770	926.8267952	64.6154098
771	928.0325783	64.5841674
772	929.237332	64.5531692
773	930.4414708	64.5216827
774	931.6458858	64.4908294
775	932.850662	64.4604949
776	934.0553695	64.4309539
777	935.2606478	64.4015731
778	936.464726	64.3726501
779	937.6688023	64.3446502
780	938.8732942	64.3161468
781	940.0773507	64.2881927
782	941.2821212	64.2598342
783	942.4869325	64.2323608
784	943.6902119	64.2043609
785	944.8941115	64.1765747
786	946.0983108	64.1481857
787	947.3024973	64.1196823
788	948.507395	64.0907516
789	949.7116441	64.060913
790	950.9163553	64.0305862
791	952.1214877	63.9996223
792	953.3270943	63.9684524
793	954.5317242	63.9367675
794	955.7368737	63.904457
795	956.9412606	63.8711318
796	958.1450692	63.8376159
797	959.3489532	63.8038253
798	960.5538744	63.7696037
799	961.7580977	63.7361755

800	962.9619944	63.7022819
801	964.1657813	63.6682357
802	965.3696715	63.6344375
803	966.5732838	63.6002578
804	967.7787634	63.5662651
805	968.9835769	63.5316238
806	970.1881188	63.4970588
807	971.3920383	63.4623298
808	972.5970139	63.427452
809	973.8022135	63.3922233
810	975.0068226	63.3575019
811	976.209948	63.3221549
812	977.4137874	63.2874679
813	978.6180047	63.2528495
814	979.8222781	63.2181205
815	981.0271082	63.1845397
816	982.2319758	63.1511116
817	983.4370236	63.1176948
818	984.6416771	63.0846176
819	985.8457233	63.0511283
820	987.0510169	63.0179634
821	988.2563472	62.98526
822	989.4613465	62.9518318
823	990.6656403	62.9187202
824	991.8702912	62.8865585
825	993.075143	62.8531417
826	994.2794059	62.8206481
827	995.4845545	62.7883605
828	996.6894312	62.7568855
829	997.8940913	62.7261352
830	999.0982635	62.6958847
831	1000.303533	62.6657829
832	1001.509334	62.6359748
833	1002.714831	62.6069869
834	1003.919039	62.5770988
835	1005.123637	62.5478401
836	1006.32996	62.5172119
837	1007.534586	62.4869995
838	1008.740764	62.4552803
839	1009.944903	62.4237251
840	1011.148761	62.3904533
841	1012.353345	62.357624
842	1013.558532	62.3249626
843	1014.763422	62.2914657
844	1015.968272	62.2579841
845	1017.172374	62.2248611
846	1018.376262	62.1921768
847	1019.581182	62.1593093
848	1020.785292	62.12751
849	1021.990308	62.0953369

850	1023.195454	62.0640754
851	1024.400209	62.0333938
852	1025.603353	62.0024528
853	1026.807364	61.9712219
854	1028.012778	61.9409408
855	1029.217324	61.9112739
856	1030.422731	61.8811874
857	1031.626755	61.8513793
858	1032.830828	61.821495
859	1034.03498	61.7924003
860	1035.240036	61.7632942
861	1036.445155	61.7341499
862	1037.649913	61.7061538
863	1038.853611	61.6779251
864	1040.058653	61.6491012
865	1041.263015	61.6205062
866	1042.468002	61.5920639
867	1043.672407	61.5642929
868	1044.876703	61.5370674
869	1046.081168	61.5089416
870	1047.286728	61.4808654
871	1048.491405	61.4530563
872	1049.695564	61.4250793
873	1050.899973	61.3973388
874	1052.104304	61.3702392
875	1053.309323	61.3425674
876	1054.514651	61.3227233
877	1055.719167	61.3015556
878	1056.923784	61.2798957
879	1058.127894	61.2591323
880	1059.332994	61.2388992
881	1060.536673	61.2183723
882	1061.740053	61.1986885
883	1062.944003	61.1790657
884	1064.148366	61.1617813
885	1065.352092	61.1462593
886	1066.557281	61.124958
887	1067.761867	61.10569
888	1068.967046	61.0873641
889	1070.172112	61.0688705
890	1071.375822	61.0510559
891	1072.580313	61.0327682
892	1073.784814	61.0137939
893	1074.989665	60.9944229
894	1076.193741	60.9735412
895	1077.397648	60.9509506
896	1078.600679	60.9275398
897	1079.806593	60.9027175
898	1081.011682	60.8775863
899	1082.216613	60.8528366

900	1083.42216	60.8278427
901	1084.626592	60.8036994
902	1085.830533	60.7799072
903	1087.034882	60.7570419
904	1088.239648	60.7340278
905	1089.443996	60.7114944
906	1090.648739	60.6888847
907	1091.853347	60.666954
908	1093.057584	60.6447257
909	1094.262676	60.6226387
910	1095.468114	60.6001472
911	1096.67357	60.5774612
912	1097.878333	60.5546989
913	1099.082696	60.5317916
914	1100.286665	60.5075607
915	1101.491736	60.4835891
916	1102.695838	60.4594001
917	1103.900936	60.4345588
918	1105.105107	60.4091377
919	1106.309231	60.3828849
920	1107.51443	60.3565254
921	1108.718626	60.3295898
922	1109.922852	60.3019828
923	1111.128243	60.2729797
924	1112.332231	60.2428779
925	1113.537228	60.2114944
926	1114.741809	60.1779594
927	1115.946623	60.1421699
928	1117.151463	60.1057434
929	1118.354698	60.0680656
930	1119.558937	60.0287361
931	1120.763385	59.989582
932	1121.967382	59.9501724
933	1123.171612	59.9118537
934	1124.375498	59.8747253
935	1125.579499	59.8379516
936	1126.783286	59.8031616
937	1127.98688	59.7701416
938	1129.190706	59.7385864
939	1130.39449	59.7084045
940	1131.599466	59.6805
941	1132.803956	59.6527633
942	1134.007943	59.6253662
943	1135.212313	59.5981826
944	1136.417089	59.5712089
945	1137.622153	59.5440483
946	1138.826808	59.5164985
947	1140.031065	59.4884834
948	1141.235959	59.4602432
949	1142.440974	59.4314575

950	1143.645979	59.402153
951	1144.850834	59.3729934
952	1146.055174	59.3432998
953	1147.259102	59.3127937
954	1148.463404	59.2836723
955	1149.668542	59.2553901
956	1150.873255	59.2281494
957	1152.077297	59.2020683
958	1153.28171	59.1755142
959	1154.485868	59.1493721
960	1155.690797	59.1231803
961	1156.895767	59.0967483
962	1158.100997	59.0698051
963	1159.3048	59.0445442
964	1160.507961	59.0182304
965	1161.712439	58.9913024
966	1162.91673	58.9631843
967	1164.121385	58.9350395
968	1165.326009	58.9064636
969	1166.530442	58.8784484
970	1167.7348	58.8499755
971	1168.939929	58.8212585
972	1170.145792	58.7932128
973	1171.350321	58.7639961
974	1172.554482	58.7354354
975	1173.758555	58.7065544
976	1174.962834	58.6785354
977	1176.16753	58.6510238
978	1177.372799	58.6242675
979	1178.577875	58.5968551
980	1179.782763	58.5707321
981	1180.988173	58.5450744
982	1182.193436	58.5195007
983	1183.398401	58.4944458
984	1184.603964	58.4685859
985	1185.807815	58.4432563
986	1187.012053	58.4178466
987	1188.217064	58.3925056
988	1189.422008	58.3675765
989	1190.6264	58.3437919
990	1191.830448	58.3184738
991	1193.034646	58.3171806
992	1194.239274	58.3140373
993	1195.443838	58.3099555
994	1196.6493	58.3055038
995	1197.853659	58.3005599
996	1199.05799	58.2948608
997	1200.262086	58.2881965
998	1201.46703	58.2816543
999	1202.671178	58.2746505

1000	1203.875467	58.2679519
1001	1205.080214	58.2375869
1002	1206.284901	58.2096977
1003	1207.489311	58.1818656
1004	1208.693911	58.1546745
1005	1209.898879	58.1281433
1006	1211.104002	58.1019973
1007	1212.307808	58.0762443
1008	1213.511895	58.0497512
1009	1214.7166	58.0223922
1010	1215.92196	57.9960937
1011	1217.126447	57.9689254
1012	1218.331464	57.9412422
1013	1219.536602	57.9145812
1014	1220.740012	57.8868827
1015	1221.945037	57.8598632
1016	1223.148816	57.8323974
1017	1224.352841	57.8053092
1018	1225.5577	57.7782516
1019	1226.761781	57.751049
1020	1227.966126	57.7235527
1021	1229.170808	57.6969413
1022	1230.374249	57.670269
1023	1231.579346	57.6428146
1024	1232.783438	57.6159858
1025	1233.98738	57.5884399
1026	1235.192306	57.5603752
1027	1236.397784	57.5318679
1028	1237.602809	57.5039176
1029	1238.807601	57.4769668
1030	1240.01223	57.4501838
1031	1241.21749	57.4221916
1032	1242.421483	57.3946838
1033	1243.626745	57.3676528
1034	1244.831624	57.3407516
1035	1246.036076	57.3137321
1036	1247.240506	57.2876815
1037	1248.445504	57.2615356
1038	1249.650892	57.2347908
1039	1250.855469	57.207386
1040	1252.060554	57.1795234
1041	1253.264603	57.1521797
1042	1254.468416	57.1255569
1043	1255.671964	57.0984382
1044	1256.876428	57.0709915
1045	1258.081764	57.0435371
1046	1259.285465	57.0162239
1047	1260.489984	56.9889526
1048	1261.694796	56.961708
1049	1262.899787	56.9346923

1050	1264.103628	56.9083976
1051	1265.308774	56.8811416
1052	1266.512135	56.8532829
1053	1267.716377	56.8263015
1054	1268.9207	56.7999343
1055	1270.125397	56.7728881
1056	1271.3306	56.7466239
1057	1272.536489	56.7206726
1058	1273.74386	56.6948928
1059	1274.94819	56.6687812
1060	1276.153326	56.6420783
1061	1277.358449	56.6168403
1062	1278.563551	56.5905838
1063	1279.767343	56.5641098
1064	1280.971319	56.537712
1065	1282.176964	56.5123672
1066	1283.381979	56.4863395
1067	1284.586124	56.4599494
1068	1285.791368	56.4336166
1069	1286.994946	56.4070434
1070	1288.199541	56.3814888
1071	1289.404511	56.3555297
1072	1290.609084	56.3298149
1073	1291.8133	56.3045425
1074	1293.017257	56.2794723
1075	1294.221563	56.2538948
1076	1295.4266	56.2284622
1077	1296.630804	56.2031631
1078	1297.835163	56.1774978
1079	1299.040128	56.1528091
1080	1300.245049	56.1272506
1081	1301.448721	56.1015815
1082	1302.653641	56.0763778
1083	1303.857788	56.0500907
1084	1305.061296	56.0234489
1085	1306.265125	55.9972724
1086	1307.46912	55.9703292
1087	1308.672625	55.943531
1088	1309.876402	55.9174385
1089	1311.080138	55.8914566
1090	1312.283989	55.8656425
1091	1313.487664	55.8401298
1092	1314.69151	55.814495
1093	1315.895517	55.7894897
1094	1317.099395	55.7644729
1095	1318.303131	55.7393913
1096	1319.506819	55.7158737
1097	1320.710616	55.6911697
1098	1321.914441	55.6663742
1099	1323.118427	55.6418533

1100	1324.322072	55.6170349
1101	1325.525807	55.5919799
1102	1326.72978	55.5671806
1103	1327.933597	55.5421981
1104	1329.137353	55.5178222
1105	1330.342148	55.4936904
1106	1331.547317	55.4683151
1107	1332.752756	55.4441757
1108	1333.95727	55.4197921
1109	1335.161676	55.3948554
1110	1336.36629	55.3702697
1111	1337.570982	55.3460922
1112	1338.775369	55.3220901
1113	1339.979999	55.2985839
1114	1341.184338	55.2748184
1115	1342.388659	55.2509689
1116	1343.593887	55.2276077
1117	1344.798994	55.2048454
1118	1346.00461	55.1830024
1119	1347.208917	55.1614189
1120	1348.412642	55.1397705
1121	1349.616273	55.1185073
1122	1350.820654	55.0974502
1123	1352.025511	55.075756
1124	1353.230644	55.0541381
1125	1354.434471	55.0328598
1126	1355.637878	55.0129776
1127	1356.841654	54.9944076
1128	1358.045854	54.9748458
1129	1359.25137	54.954895
1130	1360.456697	54.9344253
1131	1361.660063	54.9135322
1132	1362.864227	54.8923149
1133	1364.069562	54.8715934
1134	1365.274775	54.8512153
1135	1366.47937	54.8302841
1136	1367.68381	54.8083114
1137	1368.888131	54.783905
1138	1370.093532	54.7593994
1139	1371.29909	54.7359619
1140	1372.503519	54.712902
1141	1373.707685	54.6892204
1142	1374.912078	54.6662826
1143	1376.116451	54.6430244
1144	1377.321324	54.6186141
1145	1378.526346	54.5947456
1146	1379.730696	54.5703315
1147	1380.935326	54.5466423
1148	1382.13933	54.52359
1149	1383.343456	54.4995231

1150	1384.548593	54.4755477
1151	1385.753733	54.4528694
1152	1386.957518	54.4295234
1153	1388.162117	54.4068107
1154	1389.365277	54.385601
1155	1390.569511	54.3632583
1156	1391.774221	54.3415222
1157	1392.979167	54.3193588
1158	1394.184564	54.2970962
1159	1395.389748	54.2749938
1160	1396.594075	54.2526855
1161	1397.798822	54.2299194
1162	1399.004133	54.2072639
1163	1400.209047	54.1838455
1164	1401.41348	54.1595039
1165	1402.618154	54.1358032
1166	1403.822557	54.1119461
1167	1405.02757	54.0885505
1168	1406.232393	54.0650253
1169	1407.436164	54.0413818
1170	1408.640541	54.0178108
1171	1409.84487	53.9943618
1172	1411.04906	53.9704818
1173	1412.252842	53.9471206
1174	1413.457452	53.9237442
1175	1414.663182	53.9015731
1176	1415.866822	53.8783645
1177	1417.071173	53.8554
1178	1418.275904	53.8324699
1179	1419.480178	53.8094902
1180	1420.684644	53.7867126
1181	1421.888006	53.7635498
1182	1423.092273	53.7407341
1183	1424.296675	53.7183227
1184	1425.501071	53.6956481
1185	1426.705574	53.6719779
1186	1427.910864	53.6492385
1187	1429.114256	53.6264877
1188	1430.318718	53.6035652
1189	1431.52213	53.5805549
1190	1432.72582	53.5581665
1191	1433.929693	53.5371322
1192	1435.133364	53.5161628
1193	1436.337231	53.4951477
1194	1437.540593	53.474987
1195	1438.744804	53.4551277
1196	1439.948926	53.4353942
1197	1441.153344	53.4146194
1198	1442.358209	53.3948745
1199	1443.562897	53.3750915

1200	1444.767126	53.3551635
1201	1445.971871	53.3337097
1202	1447.176719	53.312294
1203	1448.381766	53.2907714
1204	1449.58802	53.2691154
1205	1450.79156	53.2470932
1206	1451.996291	53.2250595
1207	1453.200808	53.2042541
1208	1454.40426	53.1837692
1209	1455.608436	53.1638679
1210	1456.812089	53.1444015
1211	1458.01647	53.1258163
1212	1459.220466	53.1080322
1213	1460.425327	53.0900993
1214	1461.629958	53.07201
1215	1462.834441	53.0559158
1216	1464.039221	53.0409851
1217	1465.244494	53.0263366
1218	1466.449123	53.0108833
1219	1467.653826	52.9958
1220	1468.857798	52.98069
1221	1470.062334	52.9656753
1222	1471.267167	52.9494628
1223	1472.473657	52.9335212
1224	1473.678602	52.9178581
1225	1474.884024	52.8999519
1226	1476.088523	52.8803482
1227	1477.292994	52.8599777
1228	1478.497382	52.8398742
1229	1479.702804	52.819458
1230	1480.906312	52.7976455
1231	1482.110415	52.7756042
1232	1483.314548	52.753334
1233	1484.518459	52.730915
1234	1485.722353	52.707653
1235	1486.926811	52.6858024
1236	1488.131722	52.6637077
1237	1489.336355	52.6410751
1238	1490.540549	52.6183586
1239	1491.745426	52.5952644
1240	1492.949799	52.5724563
1241	1494.154171	52.5497055
1242	1495.357852	52.5265617
1243	1496.561404	52.5025253
1244	1497.765262	52.4787788
1245	1498.969622	52.4544868
1246	1500.173949	52.4317512
1247	1501.3787	52.4095573
1248	1502.583486	52.3875846
1249	1503.787679	52.3661422

1250	1504.992678	52.3453369
1251	1506.197549	52.3252182
1252	1507.402866	52.3062858
1253	1508.607076	52.2890815
1254	1509.812162	52.2726135
1255	1511.016753	52.2561416
1256	1512.221368	52.238758
1257	1513.426719	52.2222099
1258	1514.632028	52.2056159
1259	1515.83704	52.1892395
1260	1517.041638	52.1725349
1261	1518.245309	52.1553344
1262	1519.449476	52.1390686
1263	1520.653902	52.1216049
1264	1521.858675	52.1045303
1265	1523.062608	52.0869216
1266	1524.266496	52.0697555
1267	1525.470597	52.0518722
1268	1526.67527	52.0342788
1269	1527.8801	52.0166244
1270	1529.084199	51.999504
1271	1530.289238	51.982769
1272	1531.493114	51.9649467
1273	1532.697362	51.9470329
1274	1533.90234	51.9285469
1275	1535.107658	51.9111557
1276	1536.311751	51.8939285
1277	1537.515558	51.8767623
1278	1538.720685	51.8584976
1279	1539.925565	51.8405647
1280	1541.130928	51.8216781
1281	1542.33629	51.8028869
1282	1543.541081	51.7844963
1283	1544.745469	51.7663993
1284	1545.949734	51.7480049
1285	1547.155349	51.7292785
1286	1548.360282	51.7102851
1287	1549.56515	51.6911125
1288	1550.768453	51.6724472
1289	1551.973021	51.6540946
1290	1553.177158	51.6361618
1291	1554.381391	51.6179313
1292	1555.585756	51.5994262
1293	1556.790347	51.5808334
1294	1557.99482	51.5623703
1295	1559.199222	51.5442428
1296	1560.403549	51.5257949
1297	1561.60907	51.5069351
1298	1562.81347	51.4886779
1299	1564.016875	51.4691963

1300	1565.221778	51.4498176
1301	1566.426122	51.4295959
1302	1567.63109	51.410057
1303	1568.836908	51.3913688
1304	1570.042293	51.3728332
1305	1571.247028	51.3528671
1306	1572.451357	51.3328781
1307	1573.65642	51.313404
1308	1574.861588	51.2938003
1309	1576.066074	51.2737846
1310	1577.270253	51.2544555
1311	1578.47453	51.2350234
1312	1579.678084	51.2145614
1313	1580.882452	51.1943092
1314	1582.087278	51.1743888
1315	1583.292395	51.1549987
1316	1584.496197	51.1360893
1317	1585.700481	51.1172027
1318	1586.904845	51.097351
1319	1588.10928	51.0786209
1320	1589.312841	51.0593338
1321	1590.518121	51.0404739
1322	1591.722506	51.0225067
1323	1592.927115	51.0039596
1324	1594.131286	50.9850654
1325	1595.336171	50.9659347
1326	1596.541161	50.9466705
1327	1597.745255	50.9278793
1328	1598.949443	50.9098739
1329	1600.153183	50.8910675
1330	1601.357743	50.8725013
1331	1602.562125	50.8546676
1332	1603.767608	50.8353195
1333	1604.972453	50.8157119
1334	1606.176117	50.7965507
1335	1607.379998	50.7776031
1336	1608.585029	50.7588958
1337	1609.789513	50.739746
1338	1610.993221	50.7206344
1339	1612.197917	50.7014045
1340	1613.402944	50.6811599
1341	1614.60717	50.6602363
1342	1615.812303	50.639801
1343	1617.016798	50.6184577
1344	1618.221915	50.5968551
1345	1619.426449	50.5752487
1346	1620.631411	50.5529403
1347	1621.836742	50.5308151
1348	1623.040996	50.5086402
1349	1624.244591	50.4869499

1350	1625.448606	50.4687118
1351	1626.653527	50.4501266
1352	1627.858201	50.4323577
1353	1629.06228	50.4156188
1354	1630.267339	50.3989944
1355	1631.470876	50.3824348
1356	1632.685546	50.3657913
1357	1633.890901	50.3488922
1358	1635.095525	50.332447
1359	1636.300656	50.3161239
1360	1637.505098	50.2970314
1361	1638.709021	50.277687
1362	1639.913205	50.2586669
1363	1641.118224	50.2389335
1364	1642.322969	50.2190246
1365	1643.527014	50.1992187
1366	1644.731766	50.1801567
1367	1645.936274	50.1609001
1368	1647.141876	50.1414871
1369	1648.346843	50.1218452
1370	1649.551887	50.102806
1371	1650.755301	50.084053
1372	1651.959217	50.0654373
1373	1653.164178	50.0478858
1374	1654.367809	50.0296173
1375	1655.573089	50.0117111
1376	1656.777529	49.9940834
1377	1657.982467	49.9761962
1378	1659.18698	49.9581069
1379	1660.391674	49.9402885
1380	1661.596695	49.9220161
1381	1662.800876	49.9036026
1382	1664.004375	49.884716
1383	1665.208941	49.8660812
1384	1666.413902	49.8484344
1385	1667.617431	49.8307456
1386	1668.822312	49.8125305
1387	1670.027031	49.7952308
1388	1671.231486	49.7774848
1389	1672.435426	49.7602005
1390	1673.639293	49.7434501
1391	1674.84381	49.7270736
1392	1676.048363	49.7107315
1393	1677.252066	49.69384
1394	1678.456476	49.6772613
1395	1679.660663	49.6603164
1396	1680.865202	49.6439361
1397	1682.069577	49.627182
1398	1683.27412	49.611679
1399	1684.477828	49.5959434

1400	1685.681179	49.5800437
1401	1686.886885	49.5647048
1402	1688.092172	49.5499305
1403	1689.297682	49.5347366
1404	1690.502692	49.5193061
1405	1691.706403	49.5036926
1406	1692.910829	49.4867935
1407	1694.115016	49.4708023
1408	1695.320184	49.4537582
1409	1696.525711	49.4360084
1410	1697.729695	49.4182968
1411	1698.933431	49.4001045
1412	1700.137958	49.3816223
1413	1701.343041	49.3639793
1414	1702.548082	49.3452377
1415	1703.75234	49.327095
1416	1704.955853	49.3097534
1417	1706.160471	49.2927131
1418	1707.365582	49.2755622
1419	1708.571147	49.2587547
1420	1709.776327	49.2417831
1421	1710.980391	49.2247695
1422	1712.184991	49.2072792
1423	1713.390408	49.1893539
1424	1714.595424	49.172676
1425	1715.799694	49.1555175
1426	1717.004667	49.1388664
1427	1718.209783	49.1217842
1428	1719.413822	49.1052246
1429	1720.618765	49.0885963
1430	1721.82288	49.0718727
1431	1723.027041	49.0555114
1432	1724.232135	49.0385284
1433	1725.436389	49.0218429
1434	1726.641368	49.0051574
1435	1727.84589	48.9878807
1436	1729.051157	48.970642
1437	1730.25602	48.9531021
1438	1731.461091	48.9356002
1439	1732.66536	48.9179801
1440	1733.87027	48.8997344
1441	1735.075641	48.8805923
1442	1736.279606	48.8619689
1443	1737.483399	48.8429985
1444	1738.687391	48.8234519
1445	1739.891071	48.8045425
1446	1741.094943	48.7852554
1447	1742.298507	48.7649765
1448	1743.503703	48.7441329
1449	1744.707527	48.7233047

1450	1745.911924	48.7026443
1451	1747.117333	48.6824569
1452	1748.321802	48.6630859
1453	1749.526391	48.6437454
1454	1750.731327	48.6241798
1455	1751.935883	48.6045417
1456	1753.13923	48.5844116
1457	1754.34355	48.5647354
1458	1755.547662	48.5446968
1459	1756.753102	48.5239334
1460	1757.957129	48.5031471
1461	1759.162493	48.4817657
1462	1760.367324	48.4593276
1463	1761.571624	48.4363174
1464	1762.775892	48.4134178
1465	1763.981373	48.3901443
1466	1765.185353	48.3665695
1467	1766.389214	48.3427925
1468	1767.594209	48.3191299
1469	1768.798929	48.2956962
1470	1770.004078	48.2727127
1471	1771.208435	48.2502059
1472	1772.412791	48.2276496
1473	1773.617358	48.2048606
1474	1774.822146	48.1817512
1475	1776.027222	48.1589431
1476	1777.232415	48.1366271
1477	1778.437549	48.1146163
1478	1779.642064	48.0932006
1479	1780.846711	48.0724563
1480	1782.050624	48.0520706
1481	1783.255736	48.0312461
1482	1784.460631	48.0107727
1483	1785.664243	47.9913253
1484	1786.867655	47.9727363
1485	1788.071966	47.9539985
1486	1789.276549	47.9358901
1487	1790.481282	47.9175376
1488	1791.685205	47.8988227
1489	1792.889284	47.8796806
1490	1794.093871	47.8600845
1491	1795.299121	47.841732
1492	1796.503771	47.8230094
1493	1797.708054	47.8036575
1494	1798.911563	47.783802
1495	1800.115657	47.7645378
1496	1801.319791	47.7447853
1497	1802.523336	47.7254524
1498	1803.727678	47.7060127
1499	1804.932518	47.6877555

1500	1806.203	47.668621
1501	1807.406747	47.6490402
1502	1808.611499	47.6298828
1503	1809.815904	47.6117019
1504	1811.019256	47.5929718
1505	1812.223915	47.5742759
1506	1813.428139	47.5561485
1507	1814.632671	47.5378723
1508	1815.836148	47.5204467
1509	1817.039921	47.5026016
1510	1818.243322	47.4852294
1511	1819.447516	47.4686698
1512	1820.651537	47.4515228
1513	1821.856645	47.4338417
1514	1823.061755	47.4174728
1515	1824.265736	47.4008865
1516	1825.469281	47.383625
1517	1826.673938	47.3663253
1518	1827.877836	47.3490943
1519	1829.08254	47.3317413
1520	1830.287131	47.315689
1521	1831.491084	47.2991027
1522	1832.69818	47.2836685
1523	1833.901478	47.2684593
1524	1835.106119	47.2529067
1525	1836.310916	47.2378959
1526	1837.51533	47.223484
1527	1838.720344	47.2096252
1528	1839.925079	47.1950988
1529	1841.129698	47.1818809
1530	1842.335099	47.1680526
1531	1843.540033	47.1539688
1532	1844.74379	47.1403923
1533	1845.951933	47.1268501
1534	1847.156588	47.1132278
1535	1848.360508	47.0992088
1536	1849.564427	47.0848693
1537	1850.768252	47.0706977
1538	1851.973154	47.0569992
1539	1853.176706	47.0423088
1540	1854.381483	47.028057
1541	1855.587177	47.0143127
1542	1856.79079	46.9997482
1543	1857.995434	46.9855346
1544	1859.199424	46.9712257
1545	1860.404062	46.9573707
1546	1861.6086	46.9435157
1547	1862.813694	46.9300079
1548	1864.018776	46.9161491
1549	1865.223587	46.9024391

1550	1866.42833	46.8895149
1551	1867.632429	46.8761367
1552	1868.83624	46.8638992
1553	1870.040091	46.8512115
1554	1871.243907	46.8390655
1555	1872.447759	46.8268661
1556	1873.652156	46.814579
1557	1874.856637	46.8019409
1558	1876.061014	46.7903442
1559	1877.266203	46.7779388
1560	1878.470198	46.764656
1561	1879.674676	46.7509841
1562	1880.879209	46.7366752
1563	1882.083942	46.7230339
1564	1883.289074	46.7085189
1565	1884.494314	46.6949348
1566	1885.698427	46.6812667
1567	1886.902429	46.6672554
1568	1888.105983	46.6526718
1569	1889.30978	46.6385002
1570	1890.514986	46.6238899
1571	1891.719044	46.6107254
1572	1892.923129	46.5968627
1573	1894.12792	46.5829124
1574	1895.332113	46.5691719
1575	1896.537187	46.5547409
1576	1897.742724	46.5407714
1577	1898.947832	46.5271797
1578	1900.152045	46.5133628
1579	1901.356568	46.5005645
1580	1902.560992	46.4879684
1581	1903.7663	46.4743385
1582	1904.971357	46.461811
1583	1906.175557	46.4487609
1584	1907.380111	46.436016
1585	1908.584755	46.4228439
1586	1909.789164	46.4097709
1587	1910.993821	46.3971176
1588	1912.198349	46.3838729
1589	1913.402026	46.3701782
1590	1914.60645	46.3563537
1591	1915.81103	46.3430061
1592	1917.015702	46.3296585
1593	1918.220009	46.3169937
1594	1919.4234	46.3039855
1595	1920.627773	46.2917594
1596	1921.831284	46.2789497
1597	1923.035776	46.2657203
1598	1924.240339	46.2529525
1599	1925.445676	46.2404708

1600	1926.65066	46.227909
1601	1927.855075	46.2146873
1602	1929.059794	46.2011642
1603	1930.264179	46.1876869
1604	1931.468771	46.1752128
1605	1932.673569	46.1619796
1606	1933.877523	46.1499252
1607	1935.08231	46.1376647
1608	1936.286837	46.1257476
1609	1937.491662	46.1138801
1610	1938.6968	46.1025161
1611	1939.900867	46.0916366
1612	1941.104895	46.0801467
1613	1942.310128	46.0676155
1614	1943.514684	46.0544204
1615	1944.719015	46.0416183
1616	1945.923638	46.0277175
1617	1947.127449	46.0138778
1618	1948.331646	46.0001754
1619	1949.535026	45.9861335
1620	1950.739274	45.9715919
1621	1951.944051	45.9576492
1622	1953.148071	45.9442977
1623	1954.351871	45.9320144
1624	1955.556599	45.9194679
1625	1956.761205	45.9074401
1626	1957.966516	45.8955764
1627	1959.170995	45.8839454
1628	1960.375654	45.8725662
1629	1961.579761	45.8614196
1630	1962.784914	45.8505325
1631	1963.990057	45.8383865
1632	1965.195216	45.8257255
1633	1966.400138	45.8126602
1634	1967.605322	45.8003158
1635	1968.808836	45.7870674
1636	1970.01381	45.7741928
1637	1971.21847	45.7612075
1638	1972.423829	45.7479324
1639	1973.62926	45.7349472
1640	1974.834311	45.7214355
1641	1976.038692	45.7087059
1642	1977.243822	45.6971549
1643	1978.447966	45.6852912
1644	1979.652139	45.6728782
1645	1980.856026	45.6614608
1646	1982.060371	45.6490097
1647	1983.265062	45.6372299
1648	1984.469391	45.626007
1649	1985.674564	45.6139869

1650	1986.879647	45.6026039
1651	1988.083555	45.5916061
1652	1989.287813	45.5797233
1653	1990.492039	45.5685615
1654	1991.697056	45.5576019
1655	1992.902579	45.5465545
1656	1994.107069	45.5363578
1657	1995.311389	45.5257987
1658	1996.515952	45.5144653
1659	1997.721169	45.5034255
1660	1998.924406	45.4926605
1661	2000.128411	45.4812812
1662	2001.332735	45.470333
1663	2002.536905	45.4590263
1664	2003.741504	45.4480209
1665	2004.946564	45.4360084
1666	2006.151333	45.42453
1667	2007.355984	45.4138145
1668	2008.559737	45.4033889
1669	2009.763301	45.3932151
1670	2010.968766	45.3828964
1671	2012.173327	45.372734
1672	2013.377057	45.3628005
1673	2014.580936	45.3527069
1674	2015.785406	45.3429145
1675	2016.990148	45.3335533
1676	2018.194799	45.3232879
1677	2019.400329	45.3124504
1678	2020.605081	45.3003044
1679	2021.808628	45.288166
1680	2023.012732	45.276329
1681	2024.21719	45.2640762
1682	2025.422381	45.2516403
1683	2026.626869	45.2390213
1684	2027.831272	45.2258529
1685	2029.035356	45.2146606
1686	2030.240603	45.2043304
1687	2031.445813	45.1931152
1688	2032.6519	45.1831932
1689	2033.855198	45.1724472
1690	2035.059008	45.161518
1691	2036.27438	45.151287
1692	2037.479464	45.1409835
1693	2038.68398	45.1303176
1694	2039.889054	45.120018
1695	2041.09391	45.1077194
1696	2042.298057	45.0952301
1697	2043.501691	45.0832176
1698	2044.705303	45.0718727
1699	2045.909211	45.0615768

1700	2047.113083	45.0507202
1701	2048.317268	45.0401306
1702	2049.521374	45.0295219
1703	2050.726389	45.0193328
1704	2051.931261	45.0098037
1705	2053.136553	45.0001029
1706	2054.340279	44.9901962
1707	2055.544884	44.9808044
1708	2056.749267	44.9702301
1709	2057.954214	44.9598236
1710	2059.158876	44.9503059
1711	2060.363441	44.9405136
1712	2061.567904	44.9308013
1713	2062.772056	44.9219245
1714	2063.976582	44.9119033
1715	2065.181279	44.9030799
1716	2066.386673	44.8941078
1717	2067.592206	44.8856315
1718	2068.796447	44.8779373
1719	2070.00048	44.8694458
1720	2071.220095	44.8608436
1721	2072.424729	44.8525199
1722	2073.62979	44.8441543
1723	2074.833811	44.8346939
1724	2076.038278	44.8259429
1725	2077.242971	44.8165359
1726	2078.447866	44.8074607
1727	2079.652493	44.7978401
1728	2080.856749	44.7888641
1729	2082.061361	44.7797355
1730	2083.265197	44.7703933
1731	2084.469779	44.7606353
1732	2085.674693	44.7506637
1733	2086.878541	44.7412986
1734	2088.082832	44.7314834
1735	2089.287253	44.7211647
1736	2090.491354	44.7113113
1737	2091.696268	44.7009353
1738	2092.90104	44.6895561
1739	2094.10607	44.6789093
1740	2095.310567	44.6686477
1741	2096.515108	44.6581001
1742	2097.719598	44.6482543
1743	2098.924538	44.6385726
1744	2100.130173	44.6293525
1745	2101.334452	44.6203727
1746	2102.538884	44.611885
1747	2103.74287	44.6037292
1748	2104.946695	44.5961914
1749	2106.150711	44.5888442

1750	2107.355501	44.5812149
1751	2108.560707	44.573574
1752	2109.764466	44.5652084
1753	2110.969084	44.5568466
1754	2112.173368	44.5481681
1755	2113.376958	44.5394706
1756	2114.580099	44.5304985
1757	2115.784371	44.5209121
1758	2116.989164	44.5112419
1759	2118.193677	44.5015716
