# Supplementary material for: An Ultra-Compact and Low-Cost LAMP-Based Virus Detection Device
Source: Sensors (Basel). 2024 Jul 29;24(15):4912. doi: 10.3390/s24154912 (PMC11314854; doi:10.3390/s24154912)
Supplement: Supplementary file 1 [file sensors-24-04912-s001.zip › Penetration & PCM .pdf]

Number	Time (s)	Temperature (° C)
1	0.0055486	25.1891994
2	1.2104421	25.1814479
3	2.4146335	25.1800403
4	3.6187288	25.1906986
5	4.823863	25.2059383
6	6.0290794	25.2194919
7	7.2418537	25.2066745
8	8.44514	25.2090892
9	9.650244	25.207756
10	10.8540833	25.2182521
11	12.0582249	25.2211456
12	13.2629909	25.2344474
13	14.4676452	25.2411499
14	15.716389	25.2473831
15	16.9207454	25.2521476
16	18.1254113	25.2478485
17	19.33008	25.2634563
18	20.5349046	25.2717399
19	21.7391228	25.2834053
20	22.9435999	25.2798156
21	24.1487883	25.2729091
22	25.3534055	25.2537746
23	26.5578797	25.238285
24	27.7625203	25.2190437
25	28.966949	25.1966228
26	30.1701246	25.1809425
27	31.3742008	25.1608676
28	32.5791811	25.1385917
29	33.7831709	25.1159095
30	34.9875567	25.0967979
31	36.1914412	25.0905666
32	37.3962202	25.087532
33	38.6014017	25.0866127
34	39.8061146	25.0846614
35	41.0112714	25.0835094
36	42.2161433	25.0822124
37	43.419909	25.0804557
38	44.6243521	25.078762
39	45.8292459	25.0775146
40	47.0340326	25.0759239
41	48.2385029	25.0741004
42	49.44235	25.0725555
43	50.6473824	25.0713691
44	51.8514784	25.0705413
45	53.0567355	25.0690689
46	54.261245	25.0675868
47	55.4654072	25.0670318
48	56.6690358	25.0672473
49	57.8737708	25.0665569

50	59.0792477	25.0668525
51	60.2831417	25.0679721
52	61.4875546	25.0691871
53	62.6920905	25.0707702
54	63.8961749	25.0721092
55	65.1013083	25.0743808
56	66.3071985	25.0772209
57	67.5124314	25.0800647
58	68.7158854	25.0826416
59	69.9192567	25.0848503
60	71.1241073	25.0877399
61	72.3291794	25.0905189
62	73.5330488	25.0933876
63	74.7376004	25.0961418
64	75.941393	25.1003608
65	77.1463174	25.1039199
66	78.3500741	25.1077365
67	79.5542137	25.1127204
68	80.7584009	25.1176509
69	81.9622182	25.1240749
70	83.1659886	25.1295585
71	84.3707306	25.1358108
72	85.5765513	25.1419486
73	86.7810122	25.1495094
74	87.9884671	25.1573829
75	89.1922609	25.166233
76	90.3962058	25.1759395
77	91.6014359	25.1852474
78	92.806591	25.1952991
79	94.0115124	25.2053298
80	95.2159657	25.2167816
81	96.419645	25.2285118
82	97.6246314	25.2414665
83	98.8289687	25.2543067
84	100.0340804	25.2670879
85	101.2387427	25.2804965
86	102.4427235	25.2940177
87	103.6465504	25.3071479
88	104.8514503	25.3200454
89	106.0556835	25.3330497
90	107.2597828	25.346035
91	108.464428	25.3581581
92	109.6680807	25.3700771
93	110.8719232	25.3829555
94	112.0764572	25.3963508
95	113.2805287	25.4098453
96	114.4844952	25.4248294
97	115.6888349	25.4405803
98	116.8931731	25.4584064
99	118.0976773	25.4782524

100	119.3026835	25.499731
101	120.5067679	25.5241146
102	121.7106358	25.5517997
103	122.9154009	25.5834388
104	124.1200368	25.6195335
105	125.324503	25.6611289
106	126.5296572	25.7091636
107	127.7352079	25.7627906
108	128.9393373	25.8237304
109	130.1431894	25.8906364
110	131.3490969	25.9665374
111	132.5537808	26.0497798
112	133.7581287	26.141613
113	134.9620038	26.2403831
114	136.167342	26.348339
115	137.3715831	26.4645423
116	138.5760274	26.5903205
117	139.7808871	26.7279853
118	140.9850392	26.8726196
119	142.1900547	27.0283088
120	143.3947936	27.1942958
121	144.5994407	27.3711547
122	145.803429	27.5586547
123	147.0073605	27.7596073
124	148.212135	27.9698638
125	149.4164925	28.19038
126	150.6209276	28.418436
127	151.8255503	28.6556358
128	153.0310676	28.9024925
129	154.2359928	29.157505
130	155.4401853	29.4183406
131	156.6459736	29.6882419
132	157.8497713	29.9687004
133	159.0546392	30.2500305
134	160.2588767	30.5304012
135	161.4639555	30.8122253
136	162.669895	31.0917167
137	163.8744521	31.3698177
138	165.0788845	31.6513061
139	166.2834375	31.9355983
140	167.4868867	32.2271385
141	168.6916166	32.5388603
142	169.8961108	32.8515129
143	171.1001802	33.1644363
144	172.3042055	33.4801216
145	173.5089008	33.7951316
146	174.7134237	34.1284255
147	175.9174397	34.4682235
148	177.120793	34.7984008
149	178.3249215	35.1245956

150	179.5307249	35.4430999
151	180.7359991	35.7364463
152	181.9412246	36.0140495
153	183.1462015	36.2926406
154	184.3492081	36.5768814
155	185.5535954	36.8707885
156	186.7579125	37.1599693
157	187.9630333	37.4568862
158	189.1679514	37.7614517
159	190.3720691	38.0677261
160	191.5756903	38.36166
161	192.7812291	38.6497726
162	193.9850557	38.9164772
163	195.1901769	39.1552619
164	196.3952589	39.3663482
165	197.5992538	39.5517616
166	198.8030731	39.713356
167	200.0083616	39.8532714
168	201.2132524	39.9754295
169	202.4168252	40.0900306
170	203.6205824	40.2096176
171	204.824354	40.3270034
172	206.0281674	40.4664459
173	207.2319078	40.6171607
174	208.4357529	40.781311
175	209.640108	40.9502792
176	210.8439715	41.1311836
177	212.046867	41.3147506
178	213.2518075	41.5102958
179	214.4560555	41.7132949
180	215.6605448	41.9099044
181	216.8654387	42.0862998
182	218.0689206	42.2509689
183	219.2749682	42.4234619
184	220.4793309	42.6015167
185	221.6829085	42.8056945
186	222.8879241	43.0052337
187	224.0930274	43.1922721
188	225.2975513	43.3708457
189	226.5022661	43.5396499
190	227.707451	43.7034835
191	228.9117553	43.8745193
192	230.1162052	44.0420799
193	231.3200327	44.1834297
194	232.5247009	44.3010292
195	233.7290392	44.3847885
196	234.9339195	44.4645462
197	236.1390802	44.5530815
198	237.3431265	44.6481781
199	238.5475725	44.7614669

200	239.7523941	44.8997268
201	240.9574569	45.0529861
202	242.1630406	45.2263565
203	243.368108	45.4402389
204	244.5722513	45.6733207
205	245.7765103	45.8969802
206	246.9812831	46.1095085
207	248.1859092	46.3169631
208	249.3903105	46.5245018
209	250.5952679	46.7487869
210	251.7995618	46.9876708
211	253.0047713	47.3146286
212	254.2090267	47.7029075
213	255.413972	48.064476
214	256.6188301	48.4198265
215	257.8232487	48.7407684
216	259.0275652	49.018608
217	260.2332419	49.2787857
218	261.4377013	49.4942855
219	262.6422495	49.6580543
220	263.8472045	49.7464752
221	265.0516698	49.7182159
222	266.2561718	49.6281967
223	267.4612002	49.5569686
224	268.6662046	49.4998207
225	269.8704748	49.4804916
226	271.0752542	49.4803619
227	272.2801232	49.49897
228	273.4840078	49.5504188
229	274.6876668	49.6039657
230	275.8924702	49.6754226
231	277.0966921	49.7433052
232	278.3010312	49.7922286
233	279.5063053	49.8310737
234	280.7109183	49.8495712
235	281.9157401	49.8537292
236	283.1208107	49.8574104
237	284.324863	49.8439025
238	285.5290961	49.8184814
239	286.7343026	49.7907943
240	287.9390677	49.8198928
241	289.1434042	49.9048728
242	290.3493314	49.9899291
243	291.553336	50.0496749
244	292.7572646	50.1020584
245	293.9617847	50.1872711
246	295.1666135	50.3177604
247	296.3704433	50.4774208
248	297.5749848	50.6725196
249	298.7794666	50.9010543

250	299.9855128	51.1076393
251	301.1904854	51.3003158
252	302.3965656	51.5233764
253	303.6011703	51.7732238
254	304.8057337	52.0470046
255	306.0094987	52.3049926
256	307.2141661	52.5511283
257	308.4180739	52.7828369
258	309.6229019	52.9907531
259	310.8290121	53.1726188
260	312.0329699	53.3336601
261	313.2368407	53.473011
262	314.4431896	53.5724563
263	315.6489054	53.6506462
264	316.8540691	53.7178039
265	318.0594963	53.7733268
266	319.262707	53.8217544
267	320.4672947	53.8801651
268	321.6713782	53.9530029
269	322.8765973	54.0569648
270	324.0815854	54.2317733
271	325.2861565	54.4147949
272	326.4911201	54.6273918
273	327.6967473	54.8607482
274	328.9016838	55.1022109
275	330.1064033	55.3629035
276	331.3108828	55.6220588
277	332.514373	55.8727302
278	333.7184634	56.1134452
279	334.9234055	56.3272171
280	336.1282617	56.4801101
281	337.3329286	56.6437377
282	338.5367325	56.7979125
283	339.7406726	56.9479141
284	340.9454205	57.0917167
285	342.1490311	57.2422866
286	343.3539557	57.3933715
287	344.5590771	57.537178
288	345.7628004	57.6741714
289	346.9676861	57.8051033
290	348.1729271	57.9271545
291	349.3786466	58.0287094
292	350.5831259	58.1201095
293	351.7874116	58.2054557
294	352.992138	58.2841567
295	354.1964433	58.3507614
296	355.4008198	58.4174995
297	356.6060509	58.4850044
298	357.81141	58.5529098
299	359.016362	58.6200637

300	360.2204131	58.6872749
301	361.425558	58.7552986
302	362.6306226	58.8233528
303	363.8352913	58.8914413
304	365.0388364	58.958992
305	366.2430912	59.0275382
306	367.4477789	59.0955696
307	368.6537477	59.1639289
308	369.8584798	59.231842
309	371.0628297	59.2999992
310	372.266723	59.3679924
311	373.4711569	59.4355583
312	374.6759355	59.5038871
313	375.8809882	59.5722274
314	377.0863927	59.6411361
315	378.2914722	59.7094039
316	379.4962749	59.7774963
317	380.7007909	59.8457946
318	381.9060957	59.9142379
319	383.1104015	59.9835815
320	384.315354	60.0534133
321	385.5188965	60.1236038
322	386.7232596	60.1936264
323	387.9285007	60.2634658
324	389.1336563	60.3333053
325	390.3382711	60.4042587
326	391.5425521	60.475502
327	392.746716	60.5473213
328	393.9514803	60.6191825
329	395.1564257	60.6910705
330	396.3609145	60.7632522
331	397.5652452	60.8357963
332	398.7697256	60.9079284
333	399.9734462	60.9808654
334	401.1782702	61.0539245
335	402.3831114	61.1257629
336	403.5880337	61.19738
337	404.7920392	61.2691459
338	405.9969597	61.3414382
339	407.2013741	61.4124565
340	408.4051847	61.4837341
341	409.6095638	61.5532722
342	410.8145717	61.6232719
343	412.019262	61.6919364
344	413.222852	61.7599678
345	414.427218	61.8270034
346	415.6323939	61.8938598
347	416.837814	61.9588623
348	418.0416654	62.0232467
349	419.2459474	62.0876579

350	420.449859	62.1505355
351	421.6540339	62.2139701
352	422.8589247	62.278553
353	424.0640707	62.341896
354	425.2695526	62.4039993
355	426.473093	62.4659347
356	427.6778002	62.5272712
357	428.8833763	62.5879707
358	430.0871854	62.6480941
359	431.2923102	62.706871
360	432.4976316	62.7647209
361	433.7014269	62.8211593
362	434.9055366	62.8741264
363	436.1103396	62.9268722
364	437.3149725	62.9793624
365	438.519276	63.0305786
366	439.7236229	63.0818176
367	440.9275227	63.1321945
368	442.1324578	63.1893196
369	443.3373876	63.2451477
370	444.5425575	63.302288
371	445.7460165	63.3590278
372	446.9506786	63.4145889
373	448.1558238	63.4692497
374	449.3604116	63.5232543
375	450.5641598	63.5768127
376	451.777862	63.627983
377	452.981356	63.6791305
378	454.1846618	63.7215347
379	455.3885528	63.7660598
380	456.5922142	63.8062438
381	457.7961257	63.8457412
382	458.9997693	63.8863296
383	460.2041258	63.9264907
384	461.4077993	63.9662704
385	462.6118022	64.0055236
386	463.8171415	64.0461044
387	465.0220386	64.0859069
388	466.2273951	64.1251754
389	467.4317754	64.1626892
390	468.6363397	64.2020111
391	469.8411705	64.2412338
392	471.0461895	64.2803649
393	472.2504969	64.319252
394	473.4551918	64.3575286
395	474.6592678	64.3961334
396	475.8645062	64.4337844
397	477.0692185	64.4753112
398	478.2735656	64.5159912
399	479.4791063	64.5563964

400	480.6830586	64.596817
401	481.8871647	64.6367721
402	483.0925301	64.6758728
403	484.2963292	64.7151184
404	485.5013454	64.7536926
405	486.7059147	64.7916183
406	487.9102723	64.8293991
407	489.1145491	64.8630218
408	490.3186361	64.8967895
409	491.5226317	64.9298629
410	492.7264437	64.9630279
411	493.9301771	64.996315
412	495.1345449	65.0290145
413	496.338495	65.0619277
414	497.5430572	65.0955123
415	498.746939	65.1293258
416	499.9508601	65.1625518
417	501.1548901	65.1950912
418	502.3591478	65.2286529
419	503.5629387	65.2617187
420	504.766868	65.2943878
421	505.9711435	65.3273849
422	507.1749257	65.3608856
423	508.3786278	65.3939437
424	509.5824529	65.4270095
425	510.7860686	65.4594116
426	511.9900545	65.4884185
427	513.193557	65.5200195
428	514.3973743	65.5338211
429	515.6010424	65.5479812
430	516.8050485	65.5420761
431	518.0092348	65.4975585
432	519.2134979	65.45298
433	520.4177309	65.4148254
434	521.6215037	65.3630218
435	522.8256445	65.2777557
436	524.0287765	65.1849746
437	525.2324689	65.0898284
438	526.4365237	65.0168304
439	527.640555	64.9149932
440	528.8443413	64.8472213
441	530.0488257	64.7933502
442	531.2528548	64.7322845
443	532.4568228	64.5776519
444	533.6606715	64.37751
445	534.8641214	64.1838836
446	536.0677186	64.0337295
447	537.2715581	63.869194
448	538.4756346	63.7065315
449	539.6801237	63.5976181

450	540.885325	63.4831962
451	542.0901629	63.378376
452	543.2940916	63.2896232
453	544.4984281	63.2966728
454	545.7033275	63.3638114
455	546.9079297	63.4165344
456	548.1127698	63.4300308
457	549.3164893	63.4478034
458	550.5210667	63.4639053
459	551.7262791	63.4601669
460	552.9311511	63.452629
461	554.1364973	63.465229
462	555.3410884	63.4640388
463	556.5446878	63.4600524
464	557.7499328	63.4607963
465	558.9545095	63.5082473
466	560.1589152	63.5734329
467	561.3632896	63.6521492
468	562.567789	63.7337875
469	563.7722494	63.8226394
470	564.9772099	63.9224433
471	566.1827938	64.033699
472	567.387953	64.1545028
473	568.5936603	64.2828826
474	569.7980116	64.4091033
475	571.001986	64.5364379
476	572.2068165	64.6583328
477	573.4123062	64.7751464
478	574.6166363	64.897232
479	575.8210527	65.0231628
480	577.0249004	65.147705
481	578.2296933	65.2604293
482	579.4349198	65.3623123
483	580.6393632	65.4556427
484	581.8442214	65.5512313
485	583.047757	65.6405792
486	584.2526058	65.7121429
487	585.4580589	65.7898712
488	586.6627739	65.8623504
489	587.8672025	65.9382476
490	589.071841	66.020874
491	590.2769533	66.1766433
492	591.4821845	66.3413009
493	592.6863712	66.5024642
494	593.8910626	66.6067047
495	595.0957105	66.7153549
496	596.2993663	66.869522
497	597.5049804	67.019165
498	598.7091372	67.1851882
499	599.9141797	67.3196258

500	601.118652	67.3965759
501	602.323061	67.4017486
502	603.5263474	67.4074478
503	604.7301562	67.4140853
504	605.9341164	67.4919281
505	607.1391002	67.5870819
506	608.3444281	67.668312
507	609.5486459	67.7652511
508	610.7530189	67.8644561
509	611.9574341	67.9869842
510	613.1616057	68.1343231
511	614.3664234	68.2749176
512	615.5721533	68.4083023
513	616.7769748	68.5376815
514	617.9812835	68.6536102
515	619.1859585	68.768486
516	620.3913028	68.8846893
517	621.5964814	68.9871826
518	622.8007689	69.0675125
519	624.0048335	69.1455383
520	625.2090039	69.2413558
521	626.4138008	69.3429565
522	627.6180045	69.4578094
523	628.8227095	69.5789108
524	630.0269702	69.6935577
525	631.2303971	69.7866363
526	632.4353056	69.8586425
527	633.6407434	69.9297943
528	634.8454209	69.9973678
529	636.0493667	70.0558471
530	637.2524939	70.1110382
531	638.4568709	70.1586456
532	639.6622149	70.1948165
533	640.8674914	70.2255859
534	642.073564	70.2537384
535	643.2769666	70.2823638
536	644.4814461	70.3098373
537	645.686886	70.3385314
538	646.8912879	70.3633193
539	648.096149	70.3865432
540	649.3009721	70.4088439
541	650.5049078	70.4324951
542	651.7088831	70.4574813
543	652.9127401	70.482727
544	654.1175213	70.5071258
545	655.3229385	70.5322418
546	656.5268963	70.556961
547	657.7311966	70.5787734
548	658.9353922	70.5901794
549	660.1395119	70.5991058

550	661.344097	70.5987472
551	662.5489588	70.5922698
552	663.7536415	70.580162
553	664.9573819	70.5641021
554	666.1614252	70.5512542
555	667.3652535	70.5375518
556	668.5694748	70.5243911
557	669.7739183	70.5085601
558	670.9782764	70.5065689
559	672.182073	70.5077896
560	673.386216	70.5145416
561	674.5904806	70.5275955
562	675.7944144	70.5431365
563	676.998235	70.5601119
564	678.2019685	70.5794677
565	679.4076359	70.5989227
566	680.6125985	70.6216583
567	681.817985	70.6493682
568	683.0213784	70.6716308
569	684.2252849	70.693077
570	685.4298502	70.7163009
571	686.6348293	70.7429962
572	687.8392968	70.779251
573	689.0448178	70.8127899
574	690.2487136	70.8484649
575	691.4520621	70.882492
576	692.6566907	70.9126358
577	693.861025	70.9390869
578	695.065935	70.967575
579	696.2702188	70.9955291
580	697.475211	71.0215377
581	698.6791355	71.0412292
582	699.8840263	71.0528488
583	701.0895257	71.0676422
584	702.2945424	71.0752029
585	703.4999875	71.0836181
586	704.7048185	71.0922546
587	705.9095714	71.10009
588	707.1144591	71.1080703
589	708.3194386	71.1157684
590	709.5248428	71.122467
591	710.7301734	71.1293716
592	711.9334561	71.1358108
593	713.1383703	71.1417999
594	714.343169	71.1474075
595	715.5480753	71.1520614
596	716.7529008	71.1562347
597	717.9576971	71.1609497
598	719.1626503	71.165802
599	720.3675719	71.1701049

600	721.572	71.1747665
601	722.7766016	71.1785125
602	723.9812563	71.1824188
603	725.1859229	71.1860198
604	726.3898269	71.1895294
605	727.5941439	71.1927032
606	728.7994062	71.1951904
607	730.004244	71.1970214
608	731.2087444	71.1980133
609	732.4128121	71.1989669
610	733.6178961	71.1993331
611	734.8232818	71.2006912
612	736.0279465	71.2010955
613	737.2321751	71.2019577
614	738.4355943	71.2018585
615	739.6397898	71.2015609
616	740.8448443	71.2014541
617	742.0493616	71.2003021
618	743.2546095	71.2002182
619	744.4594483	71.1994628
620	745.6648021	71.198738
621	746.869651	71.1966705
622	748.0745242	71.1950073
623	749.279398	71.1928558
624	750.4831292	71.1919479
625	751.6879997	71.1906814
626	752.8924414	71.1891479
627	754.0966559	71.1882019
628	755.3012846	71.1857986
629	756.5056051	71.1842193
630	757.7100506	71.182434
631	758.9134271	71.182167
632	760.1180634	71.1814956
633	761.3236001	71.179634
634	762.5282986	71.1779785
635	763.7320837	71.1753997
636	764.9365532	71.1724929
637	766.1406129	71.1692733
638	767.3446817	71.166397
639	768.5496523	71.162857
640	769.7545671	71.1594238
641	770.958482	71.1551742
642	772.1632956	71.1502685
643	773.3681666	71.1462554
644	774.5726841	71.1412429
645	775.7770825	71.1364974
646	776.9812421	71.1317596
647	778.1859573	71.1276702
648	779.3904401	71.1230392
649	780.5959974	71.118637

650	781.8015366	71.1138534
651	783.005517	71.1088409
652	784.2093148	71.1045761
653	785.4130434	71.100296
654	786.6174	71.0959014
655	787.822591	71.0918731
656	789.0272942	71.0877914
657	790.2326624	71.0833892
658	791.4368634	71.0794143
659	792.6405623	71.0755844
660	793.8456851	71.0708923
661	795.0508003	71.0662994
662	796.2556487	71.0610046
663	797.4596697	71.0553817
664	798.6631515	71.0495223
665	799.8674087	71.0440444
666	801.0724512	71.0383834
667	802.2766969	71.0322265
668	803.4808429	71.0257186
669	804.7609364	71.018547
670	805.9650177	71.0114898
671	807.1760225	71.0046768
672	808.381022	70.9979248
673	809.5857789	70.9914627
674	810.7895761	70.9851913
675	811.9946566	70.9775619
676	813.199237	70.9709701
677	814.4029215	70.9634323
678	815.6236956	70.9567108
679	816.8275368	70.9501113
680	818.0312894	70.9427566
681	819.2349903	70.9357452
682	820.4388471	70.9289016
683	821.6434701	70.9222183
684	822.8489228	70.9147796
685	824.0540533	70.9083709
686	825.2588515	70.9006347
687	826.4789725	70.893341
688	827.6846189	70.8860244
689	828.8886106	70.878067
690	830.0934277	70.8710632
691	831.2983219	70.8632125
692	832.5012714	70.8550872
693	833.7055148	70.8458709
694	834.9094121	70.8374481
695	836.1144743	70.8288269
696	837.33555	70.8205184
697	838.5407199	70.8121337
698	839.7453638	70.8033294
699	840.9501168	70.7944183

700	842.1548488	70.7855606
701	843.3594564	70.7766418
702	844.5641615	70.7682342
703	845.767295	70.7608108
704	846.972726	70.7526168
705	848.1939356	70.7440643
706	849.3988112	70.7350921
707	850.6036389	70.7274017
708	851.8077888	70.7186203
709	853.0130678	70.7107162
710	854.2175303	70.7027435
711	855.4223788	70.6946182
712	856.6282713	70.6858139
713	857.8326957	70.6757965
714	859.0515212	70.665863
715	860.2564169	70.656044
716	861.4613647	70.6459503
717	862.66645	70.6347427
718	863.8711242	70.6246948
719	865.0755402	70.6138458
720	866.2797659	70.602951
721	867.4845387	70.5921478
722	868.6896725	70.5826568
723	869.9101746	70.5737228
724	871.1145995	70.565628
725	872.3179207	70.5569686
726	873.5235342	70.5490036
727	874.7285873	70.5413208
728	875.9325514	70.5327148
729	877.1371635	70.524559
730	878.3422564	70.5163726
731	879.5462631	70.5081634
732	880.7669836	70.49897
733	881.9726941	70.4898452
734	883.1775828	70.4798583
735	884.3818334	70.4698257
736	885.5860464	70.4598236
737	886.7907422	70.4493865
738	887.9961787	70.4396209
739	889.2005902	70.4294586
740	890.4051564	70.4191207
741	891.6261344	70.408554
742	892.8299323	70.3977432
743	894.0344126	70.3871917
744	895.2395674	70.3764572
745	896.4447124	70.3657989
746	897.6488615	70.3547668
747	898.8534872	70.3439407
748	900.05822	70.3324356
749	901.2634295	70.3217239

750	902.4833179	70.3113861
751	903.6878807	70.300949
752	904.8917659	70.2910919
753	906.0958258	70.2809677
754	907.3008302	70.2707748
755	908.5052647	70.2613906
756	909.7102491	70.2518386
757	910.9146856	70.2425918
758	912.1190238	70.2333526
759	913.3388169	70.2234649
760	914.5439268	70.213562
761	915.7481579	70.2035293
762	916.9537518	70.1922836
763	918.1584731	70.1816864
764	919.3623241	70.1713027
765	920.5662395	70.1603851
766	921.771086	70.149887
767	922.975748	70.1382217
768	924.1963162	70.1266937
769	925.3999545	70.1152191
770	926.6045533	70.1039199
771	927.8092212	70.0922698
772	929.0139033	70.0815658
773	930.2189349	70.0703887
774	931.4226339	70.0593719
775	932.6267179	70.0484313
776	933.83105	70.0363616
777	935.0527025	70.0252075
778	936.2581596	70.0144958
779	937.462595	70.0038681
780	938.6671148	69.9924163
781	939.872982	69.9819183
782	941.0772398	69.9717254
783	942.282817	69.9606781
784	943.4879481	69.9492797
785	944.6921335	69.9373779
786	945.9123228	69.9263229
787	947.1172899	69.9150848
788	948.3224023	69.9034729
789	949.5274461	69.8918991
790	950.7322379	69.8804397
791	951.9364128	69.8685531
792	953.1404824	69.8564376
793	954.3456773	69.8445281
794	955.551626	69.8330078
795	956.7707679	69.8214263
796	957.9739989	69.8105239
797	959.178181	69.799118
798	960.3827622	69.7874755
799	961.587565	69.7763214

800	962.7925647	69.7653045
801	963.9970232	69.7543792
802	965.2010117	69.742691
803	966.405331	69.7315368
804	967.6283292	69.7200469
805	968.8329092	69.7082061
806	970.0377135	69.6957092
807	971.242625	69.684555
808	972.4461087	69.6729278
809	973.6520259	69.6603012
810	974.8561589	69.6482391
811	976.0612245	69.6357116
812	977.2659436	69.6240463
813	978.4848097	69.6127471
814	979.6891986	69.6021881
815	980.894333	69.5917358
816	982.0990021	69.5817947
817	983.3027867	69.5705337
818	984.5063435	69.560585
819	985.7095674	69.5512008
820	986.9133657	69.5415954
821	988.1177267	69.5325469
822	989.3404607	69.5234375
823	990.5460321	69.5301895
824	991.7508121	69.5368804
825	992.9549933	69.5440368
826	994.1591063	69.5513534
827	995.3641128	69.5587463
828	996.5685479	69.5668106
829	997.7730354	69.5751419
830	998.9771832	69.5843582
831	1000.1981	69.5936508
832	1001.403201	69.6032867
833	1002.60868	69.5972518
834	1003.813857	69.5917816
835	1005.088719	69.5875701
836	1006.292808	69.5822296
837	1007.498005	69.5780029
838	1008.703332	69.5734634
839	1009.908177	69.5691375
840	1011.128357	69.5645599
841	1012.332168	69.5603027
842	1013.536724	69.556076
843	1014.741316	69.5513534
844	1015.946153	69.5464324
845	1017.151401	69.5414352
846	1018.355836	69.5369644
847	1019.559769	69.5327911
848	1020.763138	69.5276641
849	1021.986655	69.522644

850	1023.192122	69.5176544
851	1024.396288	69.5127182
852	1025.600764	69.5076751
853	1026.804515	69.5025634
854	1028.009988	69.4967498
855	1029.214331	69.490776
856	1030.41911	69.4850997
857	1031.623732	69.479721
858	1032.842758	69.4747772
859	1034.047869	69.4696197
860	1035.253683	69.4643859
861	1036.45916	69.4589385
862	1037.663685	69.4528961
863	1038.867579	69.4474029
864	1040.072232	69.4421539
865	1041.277165	69.4366455
866	1042.482576	69.4312438
867	1043.702965	69.4247283
868	1044.907258	69.4177703
869	1046.111993	69.4106597
870	1047.316814	69.4029846
871	1048.521488	69.3954849
872	1049.725721	69.3873443
873	1050.930761	69.3791046
874	1052.134185	69.3703765
875	1053.338648	69.3613128
876	1054.561519	69.3519668
877	1055.767071	69.342575
878	1056.972372	69.3330841
879	1058.177713	69.3237152
880	1059.381545	69.3141403
881	1060.587183	69.3040924
882	1061.79217	69.2952117
883	1062.996772	69.2855224
884	1064.200746	69.2762069
885	1065.422293	69.2668304
886	1066.626902	69.2575836
887	1067.830788	69.2483062
888	1069.036534	69.2386322
889	1070.241839	69.2293167
890	1071.445263	69.219345
891	1072.649189	69.2096557
892	1073.854586	69.1994857
893	1075.05965	69.1888885
894	1076.279907	69.178215
895	1077.484489	69.1678314
896	1078.689089	69.1565322
897	1079.893887	69.1451263
898	1081.097937	69.1346206
899	1082.303353	69.1232604

900	1083.50785	69.1123275
901	1084.712909	69.1003799
902	1085.917577	69.0884933
903	1087.138611	69.0772705
904	1088.343764	69.0660629
905	1089.548746	69.0542144
906	1090.753596	69.0427474
907	1091.957724	69.0314636
908	1093.162648	69.0195312
909	1094.367887	69.0080032
910	1095.572755	68.9965896
911	1096.778257	68.9852752
912	1097.997418	68.9732971
913	1099.201806	68.9620132
914	1100.406866	68.9496994
915	1101.611839	68.9380264
916	1102.816044	68.9268493
917	1104.02079	68.9149398
918	1105.224927	68.903038
919	1106.429136	68.8903427
920	1107.634191	68.8782806
921	1108.855809	68.8671417
922	1110.060604	68.8569793
923	1111.264279	68.8457565
924	1112.469107	68.8351287
925	1113.672973	68.8240203
926	1114.877882	68.8122711
927	1116.08276	68.8010864
928	1117.28779	68.7901916
929	1118.490896	68.7790756
930	1119.712221	68.766983
931	1120.91617	68.7547988
932	1122.119763	68.7425231
933	1123.323609	68.7303771
934	1124.527367	68.717926
935	1125.73286	68.7056503
936	1126.937703	68.6936492
937	1128.142209	68.6816558
938	1129.347184	68.6684646
939	1130.568459	68.6560516
940	1131.772769	68.6436691
941	1132.977121	68.6309967
942	1134.181976	68.6175384
943	1135.386744	68.6042404
944	1136.591869	68.5915679
945	1137.796876	68.5788116
946	1139.001649	68.5660476
947	1140.205823	68.5523223
948	1141.427066	68.5401153
949	1142.632411	68.5267868

950	1143.836741	68.5142288
951	1145.040332	68.5010452
952	1146.245	68.4885635
953	1147.448963	68.4756164
954	1148.654479	68.4629516
955	1149.858854	68.4494247
956	1151.062656	68.4361419
957	1152.284715	68.4236907
958	1153.489494	68.41082
959	1154.693941	68.398735
960	1155.898443	68.3865814
961	1157.103234	68.3753433
962	1158.307954	68.3628311
963	1159.512517	68.3504486
964	1160.717204	68.3376617
965	1161.922122	68.3252716
966	1163.142735	68.3128356
967	1164.34715	68.2999038
968	1165.551374	68.2865753
969	1166.756227	68.2730865
970	1167.961117	68.2599334
971	1169.165824	68.2453231
972	1170.370435	68.2315521
973	1171.574866	68.2179336
974	1172.77882	68.2042236
975	1174.000767	68.1901626
976	1175.205863	68.1759796
977	1176.410929	68.1629638
978	1177.616341	68.1500244
979	1178.820105	68.136795
980	1180.023809	68.1228485
981	1181.228394	68.1097259
982	1182.433542	68.0967559
983	1183.638519	68.0834884
984	1184.859378	68.0703582
985	1186.063138	68.0577545
986	1187.267964	68.044075
987	1188.472992	68.0296859
988	1189.678236	68.0155105
989	1190.88277	68.0019683
990	1192.086564	67.9887008
991	1193.290584	67.9749221
992	1194.494945	67.9613342
993	1195.716887	67.9480056
994	1196.921116	67.9344711
995	1198.12552	67.9207763
996	1199.330207	67.9073333
997	1200.535225	67.8940582
998	1201.739729	67.8815078
999	1202.9453	67.8677978

1000	1204.149175	67.8539581
1001	1205.370677	67.8402023
1002	1206.639954	67.8256988
1003	1207.844466	67.8113021
1004	1209.049572	67.7969818
1005	1210.255039	67.7822113
1006	1211.459186	67.7681579
1007	1212.663475	67.7541656
1008	1213.868274	67.7390594
1009	1215.07385	67.7247467
1010	1216.278682	67.7100982
1011	1217.483607	67.6966934
1012	1218.688782	67.68441
1013	1219.892823	67.67173
1014	1221.097952	67.6586151
1015	1222.301977	67.6463623
1016	1223.506685	67.6332244
1017	1224.710968	67.6199188
1018	1225.914998	67.6065826
1019	1227.119659	67.5935668
1020	1228.324837	67.5801391
1021	1229.529789	67.5661926
1022	1230.734268	67.5508422
1023	1231.967275	67.5361175
1024	1233.170348	67.5215606
1025	1234.37472	67.5064849
1026	1235.579947	67.4924392
1027	1236.785382	67.4779739
1028	1237.990475	67.4631652
1029	1239.193918	67.4487762
1030	1240.399039	67.4346542
1031	1241.603189	67.4201354
1032	1242.807831	67.4056625
1033	1244.013259	67.3915023
1034	1245.217488	67.3768844
1035	1246.421459	67.3623275
1036	1247.626503	67.3476333
1037	1248.831685	67.3337326
1038	1250.036134	67.3202819
1039	1251.241782	67.3063583
1040	1252.446417	67.2928543
1041	1253.651242	67.2793045
1042	1254.854973	67.2662277
1043	1256.059451	67.252037
1044	1257.26498	67.2386016
1045	1258.469781	67.2248306
1046	1259.674147	67.2108535
1047	1260.878607	67.1961288
1048	1262.082201	67.1814727
1049	1263.286182	67.1665191

1050	1264.489815	67.1514053
1051	1265.693208	67.1364898
1052	1266.898104	67.1212463
1053	1268.101466	67.106575
1054	1269.306952	67.0917205
1055	1270.511612	67.0779953
1056	1271.719175	67.064331
1057	1272.923447	67.0511474
1058	1274.128087	67.037712
1059	1275.33292	67.024826
1060	1276.537866	67.0113906
1061	1277.743237	66.9980621
1062	1278.947763	66.9847488
1063	1280.151567	66.9714736
1064	1281.35648	66.9577789
1065	1282.561307	66.943367
1066	1283.766594	66.9292449
1067	1284.970825	66.9143524
1068	1286.174189	66.8997116
1069	1287.378937	66.8843078
1070	1288.583866	66.868988
1071	1289.789042	66.8541107
1072	1290.993436	66.8394317
1073	1292.197241	66.8235855
1074	1293.401787	66.8085098
1075	1294.60702	66.7930831
1076	1295.812322	66.7766647
1077	1297.016818	66.7612304
1078	1298.221739	66.7456054
1079	1299.42602	66.7299499
1080	1300.629832	66.7153015
1081	1301.834223	66.7001266
1082	1303.03966	66.6850051
1083	1304.244611	66.6707077
1084	1305.448613	66.6571884
1085	1306.652445	66.6433181
1086	1307.857215	66.6299972
1087	1309.06164	66.6163482
1088	1310.266391	66.6034698
1089	1311.471293	66.589981
1090	1312.676089	66.5762939
1091	1313.879524	66.5625152
1092	1315.084514	66.5483016
1093	1316.289095	66.5353546
1094	1317.494543	66.5205535
1095	1318.699052	66.5063934
1096	1319.903247	66.4924163
1097	1321.107691	66.4777603
1098	1322.312931	66.4627456
1099	1323.517997	66.4492568

1100	1324.722904	66.4348297
1101	1325.926756	66.4203948
1102	1327.130513	66.4062957
1103	1328.334078	66.3915786
1104	1329.538568	66.3774032
1105	1330.743019	66.3624801
1106	1331.948066	66.3477249
1107	1333.151627	66.3341369
1108	1334.35587	66.3202209
1109	1335.560628	66.3050384
1110	1336.766364	66.2903594
1111	1337.971338	66.2761993
1112	1339.176516	66.2622909
1113	1340.381007	66.2477569
1114	1341.58519	66.2332
1115	1342.78954	66.2185745
1116	1343.993268	66.2047576
1117	1345.197547	66.1904678
1118	1346.401673	66.1768035
1119	1347.605367	66.1634597
1120	1348.808928	66.1505889
1121	1350.013581	66.1368789
1122	1351.217132	66.1231155
1123	1352.421687	66.1095352
1124	1353.625495	66.0967407
1125	1354.829582	66.0847244
1126	1356.035269	66.0710906
1127	1357.240431	66.0575027
1128	1358.445698	66.0432815
1129	1359.650245	66.0289916
1130	1360.854647	66.0146942
1131	1362.059257	66.0006408
1132	1363.264271	65.9862518
1133	1364.467979	65.9725646
1134	1365.67234	65.9580993
1135	1366.876182	65.9433898
1136	1368.079954	65.9295654
1137	1369.284402	65.9152679
1138	1370.48895	65.9013366
1139	1371.693585	65.8877639
1140	1372.899183	65.8741073
1141	1374.102876	65.8602752
1142	1375.306466	65.8473358
1143	1376.510757	65.8329086
1144	1377.715677	65.818901
1145	1378.919978	65.804985
1146	1380.124039	65.790657
1147	1381.3286	65.7759552
1148	1382.534621	65.7612533
1149	1383.739471	65.7464523

1150	1384.943707	65.7313385
1151	1386.148446	65.7169647
1152	1387.352331	65.7013244
1153	1388.556853	65.6869277
1154	1389.762081	65.6727371
1155	1390.967243	65.6589126
1156	1392.172178	65.644577
1157	1393.377168	65.6314849
1158	1394.581759	65.6186676
1159	1395.785718	65.6054458
1160	1396.990685	65.5920257
1161	1398.195204	65.5781936
1162	1399.40028	65.5651397
1163	1400.604797	65.5512924
1164	1401.810185	65.5365676
1165	1403.01552	65.5220947
1166	1404.219632	65.5075836
1167	1405.424703	65.4923858
1168	1406.629327	65.4764862
1169	1407.834149	65.4615249
1170	1409.038585	65.4461288
1171	1410.24323	65.431137
1172	1411.448124	65.4159393
1173	1412.653063	65.4014129
1174	1413.856631	65.3870162
1175	1415.061726	65.3727264
1176	1416.266727	65.3588714
1177	1417.47141	65.3454437
1178	1418.676239	65.3316802
1179	1419.881371	65.3180694
1180	1421.08583	65.3049926
1181	1422.289506	65.2913665
1182	1423.494711	65.2773513
1183	1424.699228	65.2630615
1184	1425.90339	65.2492675
1185	1427.107182	65.2348785
1186	1428.310858	65.2208786
1187	1429.514747	65.2060699
1188	1430.718554	65.1920166
1189	1431.922942	65.1779403
1190	1433.127523	65.16333
1191	1434.331728	65.149002
1192	1435.535906	65.1352539
1193	1436.741781	65.1210174
1194	1437.946362	65.106575
1195	1439.151681	65.0925827
1196	1440.356016	65.0782928
1197	1441.55975	65.0642395
1198	1442.763606	65.0506057
1199	1443.968447	65.0364303

1200	1445.173856	65.0231475
1201	1446.378005	65.0097427
1202	1447.582608	64.9963684
1203	1448.78652	64.983963
1204	1449.990411	64.9710769
1205	1451.193447	64.9586715
1206	1452.398098	64.9462738
1207	1453.602707	64.9342193
1208	1454.807328	64.921318
1209	1456.012038	64.909233
1210	1457.217222	64.8966293
1211	1458.421677	64.8848495
1212	1459.625813	64.8725738
1213	1460.830296	64.8603973
1214	1462.034623	64.84906
1215	1463.239183	64.8371124
1216	1464.443818	64.8255538
1217	1465.648631	64.8144302
1218	1466.853041	64.8029937
1219	1468.05735	64.7915649
1220	1469.260762	64.780548
1221	1470.465443	64.7693176
1222	1471.670318	64.7594604
1223	1472.87476	64.7488632
1224	1474.079329	64.7385482
1225	1475.284557	64.7280654
1226	1476.488702	64.7180633
1227	1477.693289	64.7079925
1228	1478.898409	64.6991958
1229	1480.103351	64.6903915
1230	1481.307606	64.681343
1231	1482.51156	64.6727447
1232	1483.716434	64.6637496
1233	1484.921245	64.6551971
1234	1486.12595	64.6468353
1235	1487.331615	64.6386489
1236	1488.536559	64.6296234
1237	1489.74064	64.6209564
1238	1490.946369	64.6113433
1239	1492.151417	64.6016082
1240	1493.356193	64.5926666
1241	1494.560641	64.5827102
1242	1495.764888	64.5728302
1243	1496.969272	64.563858
1244	1498.173491	64.5541305
1245	1499.37858	64.5443725
1246	1500.583067	64.5348815
1247	1501.787032	64.5257263
1248	1502.991395	64.5172958
1249	1504.195925	64.5086441

1250	1505.400536	64.4997177
1251	1506.605214	64.4915313
1252	1507.809358	64.4828796
1253	1509.013786	64.4745254
1254	1510.219252	64.4667816
1255	1511.423953	64.4600982
1256	1512.628207	64.4537582
1257	1513.832795	64.4471893
1258	1515.037022	64.4409255
1259	1516.241034	64.435234
1260	1517.445935	64.4299545
1261	1518.65126	64.4252243
1262	1519.856099	64.4204254
1263	1521.060976	64.4142608
1264	1522.265846	64.4082183
1265	1523.470967	64.4019775
1266	1524.6755	64.3959808
1267	1525.87975	64.3894958
1268	1527.0851	64.3834686
1269	1528.290403	64.3772048
1270	1529.49419	64.3711776
1271	1530.699241	64.3646163
1272	1531.9046	64.3585433
1273	1533.109278	64.3539962
1274	1534.314167	64.3499603
1275	1535.51802	64.3452911
1276	1536.721953	64.3411712
1277	1537.926388	64.3379516
1278	1539.131133	64.3345794
1279	1540.335932	64.3312072
1280	1541.53936	64.3272857
1281	1542.743361	64.3240203
1282	1543.947682	64.3203125
1283	1545.152956	64.3163909
1284	1546.358157	64.312088
1285	1547.563519	64.3080673
1286	1548.768311	64.3039703
1287	1549.972502	64.2995452
1288	1551.177434	64.2949676
1289	1552.381547	64.290596
1290	1553.5868	64.2874298
1291	1554.791702	64.2837066
1292	1555.99608	64.2807159
1293	1557.200346	64.277359
1294	1558.404876	64.2741317
1295	1559.609737	64.2715606
1296	1560.814178	64.2681121
1297	1562.018389	64.2648086
1298	1563.223746	64.261856
1299	1564.428408	64.2589187

1300	1565.632812	64.2559814
1301	1566.837822	64.2525482
1302	1568.043463	64.2491226
1303	1569.246532	64.2459106
1304	1570.450954	64.2429122
1305	1571.655263	64.2394943
1306	1572.860947	64.2368316
1307	1574.06612	64.2343597
1308	1575.270176	64.2318115
1309	1576.474182	64.2293548
1310	1577.677965	64.2262954
1311	1578.882649	64.2240524
1312	1580.087791	64.2217864
1313	1581.292804	64.2189178
1314	1582.497422	64.2155609
1315	1583.70225	64.2128982
1316	1584.907432	64.2106781
1317	1586.112821	64.2071304
1318	1587.317841	64.2036437
1319	1588.522178	64.1998367
1320	1589.726317	64.1963119
1321	1590.930348	64.1925125
1322	1592.134785	64.1883392
1323	1593.339809	64.1850662
1324	1594.544538	64.1816711
1325	1595.748047	64.1780624
1326	1596.951318	64.1740493
1327	1598.156402	64.1707611
1328	1599.361221	64.1670761
1329	1600.564572	64.1640014
1330	1601.769188	64.1602172
1331	1602.973637	64.1561355
1332	1604.179095	64.1530609
1333	1605.384139	64.1490783
1334	1606.588682	64.1457366
1335	1607.793489	64.1422576
1336	1608.998521	64.1385192
1337	1610.202594	64.135086
1338	1611.406959	64.1320037
1339	1612.611146	64.1281433
1340	1613.816882	64.1249465
1341	1615.021568	64.1218109
1342	1616.226445	64.1185302
1343	1617.431064	64.1148681
1344	1618.635668	64.1115646
1345	1619.841008	64.1076507
1346	1621.046563	64.1040878
1347	1622.251427	64.100296
1348	1623.455522	64.0959243
1349	1624.660109	64.0919265

1350	1625.86518	64.0875167
1351	1627.070399	64.0835342
1352	1628.275598	64.0792083
1353	1629.480735	64.0751647
1354	1630.685168	64.0706939
1355	1631.888605	64.066574
1356	1633.093475	64.0619354
1357	1634.298309	64.0576934
1358	1635.502422	64.0534744
1359	1636.706566	64.0492401
1360	1637.911036	64.0447463
1361	1639.115588	64.0395202
1362	1640.320509	64.034996
1363	1641.525143	64.0302276
1364	1642.728994	64.0254898
1365	1643.933208	64.020729
1366	1645.13759	64.0157775
1367	1646.342568	64.0106506
1368	1647.548178	64.0060043
1369	1648.752918	64.0009994
1370	1649.957015	63.9960021
1371	1651.160905	63.9915657
1372	1652.366431	63.9863471
1373	1653.570822	63.9818038
1374	1654.776673	63.9764404
1375	1655.98103	63.9713058
1376	1657.18472	63.9668273
1377	1658.388787	63.9617919
1378	1659.593021	63.9565544
1379	1660.798252	63.9524536
1380	1662.003429	63.9482307
1381	1663.207365	63.9445686
1382	1664.415694	63.9406967
1383	1665.620511	63.9370536
1384	1666.82576	63.9335708
1385	1668.031285	63.9300613
1386	1669.235385	63.9268074
1387	1670.439598	63.9236831
1388	1671.644007	63.9200515
1389	1672.849858	63.9160385
1390	1674.05541	63.91259
1391	1675.260287	63.9087524
1392	1676.465081	63.9050903
1393	1677.669296	63.9009284
1394	1678.873799	63.8972663
1395	1680.078532	63.8931198
1396	1681.283265	63.8880805
1397	1682.487394	63.8834609
1398	1683.691272	63.8793754
1399	1684.895357	63.8743591

1400	1686.100129	63.868782
1401	1687.305857	63.862812
1402	1688.510812	63.8566894
1403	1689.714786	63.8508224
1404	1690.919731	63.844902
1405	1692.12318	63.838478
1406	1693.328715	63.833271
1407	1694.533408	63.8279914
1408	1695.738223	63.8230667
1409	1696.942543	63.8183059
1410	1698.146679	63.8141098
1411	1699.3532	63.8103103
1412	1700.557665	63.8063583
1413	1701.763089	63.8025627
1414	1702.967504	63.7990188
1415	1704.171505	63.7957382
1416	1705.376416	63.7914237
1417	1706.581994	63.7869873
1418	1707.786265	63.7820358
1419	1708.991049	63.777687
1420	1710.195556	63.7729682
1421	1711.399117	63.7682609
1422	1712.604703	63.763874
1423	1713.809358	63.7591476
1424	1715.013855	63.7535476
1425	1716.218357	63.7490158
1426	1717.423312	63.7441101
1427	1718.627459	63.7390937
1428	1719.832581	63.7345275
1429	1721.037505	63.7299728
1430	1722.242844	63.7253341
1431	1723.448253	63.7203254
1432	1724.652074	63.7149581
1433	1725.856826	63.7094116
1434	1727.061927	63.7040901
1435	1728.267255	63.6988639
1436	1729.47193	63.6942024
1437	1730.67635	63.6899795
1438	1731.880442	63.6853828
1439	1733.084154	63.6801223
1440	1734.287991	63.6751594
1441	1735.491725	63.6710395
1442	1736.696044	63.667488
1443	1737.899515	63.6639595
1444	1739.103801	63.6613845
1445	1740.308824	63.6582565
1446	1741.512757	63.6548881
1447	1742.717747	63.6515274
1448	1743.922705	63.6483383
1449	1745.127119	63.6456794

1450	1746.331392	63.642395
1451	1747.5364	63.6378479
1452	1748.741264	63.6326675
1453	1749.946431	63.6280937
1454	1751.150956	63.6229019
1455	1752.355088	63.6170845
1456	1753.559312	63.611824
1457	1754.764365	63.6065597
1458	1755.969332	63.6008224
1459	1757.174219	63.5948104
1460	1758.378725	63.5893936
1461	1759.582859	63.5849533
1462	1760.788075	63.5805969
1463	1761.99202	63.5761833
1464	1763.197463	63.5721054
1465	1764.401597	63.5682487
1466	1765.604728	63.5641288
1467	1766.809129	63.5597915
1468	1768.014257	63.5554313
1469	1769.218864	63.5505905
1470	1770.423741	63.5455856
1471	1771.628405	63.5400276
1472	1772.832496	63.534069
1473	1774.037262	63.527996
1474	1775.242685	63.5216789
1475	1776.447201	63.5152587
1476	1777.651862	63.5114936
1477	1778.856231	63.5077209
1478	1780.061192	63.5049438
1479	1781.265985	63.5023193
1480	1782.470056	63.5001716
1481	1783.674442	63.4983634
1482	1784.878638	63.4970893
1483	1786.083188	63.4957962
1484	1787.287376	63.4945182
1485	1788.490986	63.4925918
1486	1789.695885	63.487751
1487	1790.900185	63.4840393
1488	1792.103748	63.4787483
1489	1793.308368	63.4748611
1490	1794.512679	63.4702072
1491	1795.718049	63.4656982
1492	1796.922506	63.4608764
1493	1798.126917	63.4557571
1494	1799.330644	63.4505233
1495	1800.53539	63.4457283
1496	1801.740025	63.4414482
1497	1802.943903	63.4363098
1498	1804.14822	63.4319763
1499	1805.352407	63.4266891

1500	1806.556617	63.4219284
1501	1807.761385	63.4167709
1502	1808.965894	63.4120254
1503	1810.170737	63.40736
1504	1811.375537	63.4028244
1505	1812.579939	63.3986892
1506	1813.78421	63.3946914
1507	1814.98925	63.3899917
1508	1816.194502	63.3861389
1509	1817.398849	63.3817825
1510	1818.603037	63.3777847
1511	1819.806365	63.3730659
1512	1821.01152	63.3684692
1513	1822.216302	63.3649177
1514	1823.421026	63.3601074
1515	1824.62473	63.3555908
1516	1825.829579	63.3496818
1517	1827.034115	63.3446884
1518	1828.238484	63.3387565
1519	1829.443243	63.3331222
1520	1830.647612	63.3268508
1521	1831.852606	63.3211898
1522	1833.057032	63.3156967
1523	1834.261466	63.3098373
1524	1835.465807	63.3051834
1525	1836.670821	63.2996902
1526	1837.875844	63.2957382
1527	1839.080139	63.2911148
1528	1840.284769	63.2863159
1529	1841.489353	63.2822494
1530	1842.694503	63.2782592
1531	1843.899076	63.2743301
1532	1845.104493	63.2710647
1533	1846.308466	63.2666091
1534	1847.51404	63.2624397
1535	1848.719159	63.2588577
1536	1849.923707	63.2550048
1537	1851.129123	63.2508621
1538	1852.333514	63.247528
1539	1853.538037	63.2436943
1540	1854.742315	63.2399291
1541	1855.946367	63.236145
1542	1857.151326	63.231224
1543	1858.355779	63.2275009
1544	1859.559086	63.2227287
1545	1860.763465	63.2183914
1546	1861.968339	63.2132682
1547	1863.173026	63.2086791
1548	1864.377249	63.2033882
1549	1865.582555	63.19804

1550	1866.786679	63.1924057
1551	1867.990983	63.1870002
1552	1869.195522	63.1817436
1553	1870.400603	63.1760025
1554	1871.605419	63.1707992
1555	1872.809355	63.1652412
1556	1874.013631	63.160057
1557	1875.219276	63.1545295
1558	1876.42465	63.1494865
1559	1877.629716	63.1450004
1560	1878.834563	63.1404914
1561	1880.038122	63.1362533
1562	1881.242915	63.1321601
1563	1882.448175	63.1277351
1564	1883.653385	63.1229972
1565	1884.857066	63.1191291
1566	1886.061211	63.1152572
1567	1887.265296	63.1121025
1568	1888.469878	63.1076736
1569	1889.674304	63.1026535
1570	1890.879362	63.0975227
1571	1892.083136	63.0919609
1572	1893.288142	63.0865974
1573	1894.492354	63.0807876
1574	1895.697295	63.0757141
1575	1896.903008	63.0695495
1576	1898.107282	63.0638618
1577	1899.312919	63.0579643
1578	1900.516353	63.0525207
1579	1901.721096	63.0471305
1580	1902.926281	63.0421524
1581	1904.131828	63.0378189
1582	1905.337232	63.0328254
1583	1906.541418	63.0278511
1584	1907.74607	63.0228157
1585	1908.950977	63.0186157
1586	1910.156437	63.0132446
1587	1911.360936	63.0073661
1588	1912.565576	63.0024642
1589	1913.769172	62.9975395
1590	1914.974334	62.9925155
1591	1916.178879	62.9873046
1592	1917.384146	62.9824523
1593	1918.588479	62.9780502
1594	1919.793332	62.9728584
1595	1920.997168	62.9677543
1596	1922.200989	62.9634895
1597	1923.405695	62.9593734
1598	1924.611111	62.9551506
1599	1925.815886	62.9505615

1600	1927.019396	62.946434
1601	1928.224027	62.9418029
1602	1929.429866	62.9371261
1603	1930.635258	62.9326095
1604	1931.839945	62.9286422
1605	1933.045058	62.9243545
1606	1934.249535	62.9197082
1607	1935.453988	62.9150772
1608	1936.658439	62.9099807
1609	1937.863264	62.9055671
1610	1939.067077	62.9006538
1611	1940.271555	62.8955574
1612	1941.477143	62.8903732
1613	1942.682338	62.8848495
1614	1943.887368	62.8796577
1615	1945.092186	62.8744125
1616	1946.296145	62.8686599
1617	1947.500289	62.8631401
1618	1948.70461	62.8575134
1619	1949.909818	62.851696
1620	1951.115048	62.8456802
1621	1952.319076	62.8400993
1622	1953.523855	62.8360481
1623	1954.728535	62.8311958
1624	1955.933915	62.8265304
1625	1957.139077	62.8219795
1626	1958.343307	62.817646
1627	1959.547059	62.8140182
1628	1960.750898	62.8103103
1629	1961.954905	62.8064994
1630	1963.159729	62.802391
1631	1964.363996	62.7959671
1632	1965.568235	62.7880477
1633	1966.772259	62.782402
1634	1967.976665	62.7762374
1635	1969.180613	62.7700119
1636	1970.385239	62.7641906
1637	1971.599137	62.7576675
1638	1972.803491	62.7519416
1639	1974.008037	62.7465591
1640	1975.212228	62.741291
1641	1976.416579	62.7378654
1642	1977.621846	62.733757
1643	1978.826492	62.7292556
1644	1980.029475	62.7237472
1645	1981.232968	62.7177543
1646	1982.437821	62.7115287
1647	1983.642486	62.7051467
1648	1984.846546	62.6986312
1649	1986.05137	62.6914291

1650	1987.255809	62.6840438
1651	1988.460423	62.6775321
1652	1989.665125	62.6714706
1653	1990.870815	62.6645431
1654	1992.07602	62.6587638
1655	1993.280559	62.6530265
1656	1994.484859	62.6472167
1657	1995.68968	62.6412963
1658	1996.894234	62.6352958
1659	1998.099789	62.6289138
1660	1999.304117	62.6236648
1661	2000.507486	62.6186103
1662	2001.71137	62.613861
1663	2002.915713	62.6087646
1664	2004.12055	62.6034431
1665	2005.325783	62.5987052
1666	2006.531044	62.5936965
1667	2007.734768	62.588665
1668	2008.938702	62.5831794
1669	2010.143339	62.5781021
1670	2011.34816	62.5725822
1671	2012.553526	62.5659561
1672	2013.758052	62.5590133
1673	2014.963081	62.5527763
1674	2016.168488	62.5463371
1675	2017.373132	62.5390701
1676	2018.578336	62.5323905
1677	2019.783386	62.5258293
1678	2020.98725	62.5197982
1679	2022.191847	62.5139694
1680	2023.395488	62.5081596
1681	2024.60147	62.5023689
1682	2025.806512	62.4974899
1683	2027.010555	62.492073
1684	2028.214196	62.48637
1685	2029.418135	62.4817008
1686	2030.622144	62.4763565
1687	2031.82654	62.4709701
1688	2033.031852	62.4649734
1689	2034.236437	62.4594497
1690	2035.441109	62.4537429
1691	2036.645448	62.447895
1692	2037.849224	62.4416313
1693	2039.053103	62.4354972
1694	2040.256783	62.4297981
1695	2041.460686	62.4237289
1696	2042.664852	62.4179992
1697	2043.869204	62.4121017
1698	2045.07378	62.4063835
1699	2046.279338	62.4006652

1700	2047.483928	62.3945312
1701	2048.688607	62.3885078
1702	2049.892761	62.3818969
1703	2051.096727	62.3755378
1704	2052.300729	62.3692626
1705	2053.505076	62.3625068
1706	2054.70987	62.3564529
1707	2055.914255	62.3509597
1708	2057.118922	62.3450431
1709	2058.323333	62.3385543
1710	2059.528334	62.3322219
1711	2060.733753	62.3264274
1712	2061.937759	62.3208427
1713	2063.142074	62.3148498
1714	2064.34713	62.3085556
1715	2065.551542	62.3021965
1716	2066.755468	62.2949829
1717	2067.960122	62.2873306
1718	2069.163851	62.2805175
1719	2070.367939	62.2730407
1720	2071.572615	62.2659225
1721	2072.777598	62.2582588
1722	2073.982161	62.2506408
1723	2075.186058	62.2440414
1724	2076.390254	62.2374877
1725	2077.594946	62.2307662
1726	2078.799704	62.2252998
1727	2080.003677	62.2189826
1728	2081.208564	62.2123527
1729	2082.413497	62.2059745
1730	2083.619697	62.1996459
1731	2084.824924	62.1935615
1732	2086.029821	62.187828
1733	2087.233618	62.1816101
1734	2088.437841	62.1750984
1735	2089.642901	62.1687736
1736	2090.84857	62.1616935
1737	2092.052905	62.1555099
1738	2093.257719	62.14859
1739	2094.462006	62.142189
1740	2095.666415	62.1358413
1741	2096.871411	62.1294746
1742	2098.076156	62.1227302
1743	2099.280811	62.1150932
1744	2100.485607	62.1084709
1745	2101.690387	62.1021919
1746	2102.895105	62.0956115
1747	2104.098864	62.0892028
1748	2105.304255	62.083702
1749	2106.509261	62.0780525

1750	2107.713151	62.0717849
1751	2108.918325	62.0653381
1752	2110.122462	62.0590515
1753	2111.326058	62.0531311
1754	2112.530375	62.046791
1755	2113.734345	62.0396232
1756	2114.938974	62.0329055
1757	2116.142968	62.0259628
1758	2117.347379	62.0181274
1759	2118.551861	62.0108451
1760	2119.757037	62.0040893
1761	2120.961313	61.9976577
1762	2122.165264	61.9910163
1763	2123.370116	61.9841804
1764	2124.574265	61.9772872
1765	2125.778121	61.9710502
1766	2126.983007	61.9636459
1767	2128.187762	61.9558105
1768	2129.390912	61.9490737
1769	2130.595098	61.9416007
1770	2131.799339	61.9337997
1771	2133.004429	61.9258651
1772	2134.20937	61.9179916
1773	2135.41371	61.9101943
1774	2136.617367	61.9022827
1775	2137.822353	61.8944625
1776	2139.02596	61.887207
1777	2140.230987	61.8801803
1778	2141.435863	61.8721694
1779	2142.639474	61.8640861
1780	2143.843927	61.8559761
1781	2145.049334	61.8481979
1782	2146.254415	61.8395004
1783	2147.459075	61.831562
1784	2148.663652	61.8235168
1785	2149.867444	61.8148727
1786	2151.072461	61.8063354
1787	2152.276392	61.7983169
1788	2153.480986	61.7898864
1789	2154.686443	61.782135
1790	2155.890259	61.7744331
1791	2157.094694	61.7661247
1792	2158.299941	61.7589797
1793	2159.504705	61.7509346
1794	2160.708328	61.7432632
1795	2161.911959	61.7356567
1796	2163.116517	61.7287902
1797	2164.321534	61.7219352
1798	2165.526504	61.715435
1799	2166.731396	61.7087821

1800	2167.936181	61.7014884
1801	2169.140934	61.6945381
1802	2170.3451	61.6870613
1803	2171.549974	61.6796798
1804	2172.755291	61.6729545
1805	2173.960369	61.6653137
1806	2175.163838	61.6570014
1807	2176.367933	61.6485824
1808	2177.571449	61.6407165
1809	2178.775469	61.6322021
1810	2179.980495	61.6240348
1811	2181.185778	61.6155281
1812	2182.390134	61.6067924
1813	2183.595169	61.5979728
1814	2184.799148	61.5882644
1815	2186.002677	61.5786552
1816	2187.207342	61.5689697
1817	2188.411547	61.558155
1818	2189.615581	61.5476303
1819	2190.819654	61.5372238
1820	2192.024935	61.527172
1821	2193.229411	61.5169906
1822	2194.434125	61.5068969
1823	2195.639107	61.4961776
1824	2196.843603	61.485279
1825	2198.048395	61.4755973
1826	2199.252742	61.4658203
1827	2200.456917	61.4562377
1828	2201.661721	61.4463615
1829	2202.866129	61.4362678
1830	2204.069382	61.425621
1831	2205.273908	61.4159164
1832	2206.478988	61.4059982
1833	2207.684143	61.3971939
1834	2208.917229	61.3878479
1835	2210.121523	61.3783645
1836	2211.325649	61.3683662
1837	2212.531367	61.3589286
1838	2213.736261	61.3496322
1839	2214.940694	61.3406906
1840	2216.144722	61.3320236
1841	2217.349145	61.3226699
1842	2218.553431	61.3129882
1843	2219.758089	61.3031082
1844	2220.96312	61.2935562
1845	2222.167513	61.282936
1846	2223.371568	61.2736206
1847	2224.57684	61.2639236
1848	2225.782043	61.2534713
1849	2226.987344	61.2432174

1850	2228.192149	61.2331352
1851	2229.396148	61.2225646
1852	2230.60055	61.2125663
1853	2231.80544	61.2021102
1854	2233.010787	61.1918716
1855	2234.216119	61.1825675
1856	2235.421003	61.1721801
1857	2236.625558	61.161499
1858	2237.830082	61.1516799
1859	2239.034753	61.1413345
1860	2240.239221	61.1313896
1861	2241.443886	61.1218757
1862	2242.64829	61.1118583
1863	2243.8527	61.1018066
1864	2245.057344	61.0924797
1865	2246.262337	61.0828781
1866	2247.466122	61.0727462
1867	2248.670679	61.0631217
1868	2249.874837	61.0529899
1869	2251.07837	61.0431594
1870	2252.282721	61.0327224
1871	2253.487221	61.0220222
1872	2254.692326	61.0113601
1873	2255.896391	61.0010566
1874	2257.100301	60.9894332
1875	2258.304801	60.9786796
1876	2259.510056	60.96809
1877	2260.714646	60.9575424
1878	2261.920425	60.9471855
1879	2263.126049	60.9369201
1880	2264.330616	60.9267959
1881	2265.535481	60.9163093
1882	2266.740959	60.9058609
1883	2267.945552	60.8955459
1884	2269.149755	60.8857727
1885	2270.353367	60.8750076
1886	2271.557211	60.8648605
1887	2272.760993	60.854103
1888	2273.964729	60.8430557
1889	2275.1697	60.8315544
1890	2276.374208	60.8196411
1891	2277.577856	60.8082504
1892	2278.782267	60.7974662
1893	2279.987237	60.7862777
1894	2281.191843	60.7751655
1895	2282.397178	60.7641181
1896	2283.60232	60.7525749
1897	2284.805885	60.7418022
1898	2286.010953	60.7310256
1899	2287.216277	60.7205734

1900	2288.420134	60.7102279
1901	2289.623901	60.6990852
1902	2290.828827	60.6882743
1903	2292.032797	60.6775741
1904	2293.237937	60.6657714
1905	2294.442242	60.6546096
1906	2295.647286	60.6439399
1907	2296.851732	60.6328811
1908	2298.05596	60.621437
1909	2299.26103	60.6101799
1910	2300.466036	60.5989761
1911	2301.670896	60.5880622
1912	2302.874252	60.577156
1913	2304.078744	60.5658645
1914	2305.283071	60.5552787
1915	2306.488323	60.5443
1916	2307.692565	60.5327835
1917	2308.897727	60.5213127
1918	2310.102929	60.510765
1919	2311.306352	60.4995536
1920	2312.511119	60.4882698
1921	2313.715602	60.4777145
1922	2314.920912	60.466381
1923	2316.123981	60.4553527
1924	2317.328543	60.4439773
1925	2318.534157	60.4320182
1926	2319.739082	60.4207382
1927	2320.943826	60.4093322
1928	2322.148245	60.3970413
1929	2323.352275	60.3853263
1930	2324.555882	60.3730773
1931	2325.760541	60.3603324
1932	2326.966622	60.3472785
1933	2328.171529	60.3344306
1934	2329.376429	60.3218765
1935	2330.581189	60.3093261
1936	2331.785986	60.2968368
1937	2332.991156	60.2841415
1938	2334.196291	60.2712287
1939	2335.401135	60.2578201
1940	2336.60558	60.2448234
1941	2337.809676	60.2319869
1942	2339.014462	60.2194938
1943	2340.218399	60.2065811
1944	2341.42811	60.1936836
1945	2342.632518	60.1811408
1946	2343.836511	60.168415
1947	2345.040277	60.1550521
1948	2346.243921	60.1429328
1949	2347.447599	60.1311225

1950	2348.651404	60.1193161
1951	2349.856198	60.1075057
1952	2351.059995	60.0958328
1953	2352.264869	60.0841903
1954	2353.469184	60.0724716
1955	2354.673482	60.0606307
1956	2355.878953	60.0482711
1957	2357.083703	60.0366592
1958	2358.287548	60.0239181
1959	2359.492437	60.010601
1960	2360.69778	59.9974479
1961	2361.902205	59.9842453
1962	2363.107174	59.9711532
1963	2364.312603	59.9578247
1964	2365.516519	59.9449348
1965	2366.721888	59.9317016
1966	2367.927532	59.9192504
1967	2369.13306	59.9067993
1968	2370.338592	59.8947372
1969	2371.543586	59.882698
1970	2372.747392	59.8708114
1971	2373.95201	59.8583908
1972	2375.156813	59.8455734
1973	2376.361532	59.8334426
1974	2377.566299	59.8204879
1975	2378.77105	59.8079948
1976	2379.975361	59.7948303
1977	2381.180108	59.7811813
1978	2382.385096	59.7679634
1979	2383.589795	59.755146
1980	2384.794286	59.7411613
1981	2385.998425	59.7280807
1982	2387.203099	59.7151985
1983	2388.407882	59.7011337
1984	2389.613081	59.6877822
1985	2390.817526	59.6739501
1986	2392.021908	59.6598587
1987	2393.226989	59.6464843
1988	2394.431226	59.6327285
1989	2395.63558	59.6187324
1990	2396.840515	59.6052856
1991	2398.044338	59.5917053
1992	2399.24888	59.5778503
1993	2400.453947	59.5645446
1994	2401.659012	59.5507392
1995	2402.864161	59.5372619
1996	2404.068081	59.5241508
1997	2405.272172	59.5105171
1998	2406.47601	59.4969367
1999	2407.680748	59.483406

2000	2408.886189	59.4698791
2001	2410.091184	59.4555587
2002	2411.295647	59.4415588
2003	2412.499577	59.4276237
2004	2413.704078	59.4134445
2005	2414.909118	59.3980407
2006	2416.114012	59.383068
2007	2417.318855	59.368225
2008	2418.522997	59.353569
2009	2419.726258	59.3382759
2010	2420.931247	59.323143
2011	2422.136349	59.3094558
2012	2423.340949	59.2959289
2013	2424.545301	59.2827033
2014	2425.749885	59.2697753
2015	2426.954475	59.2583084
2016	2428.15845	59.2464599
2017	2429.363396	59.2341308
2018	2430.567995	59.22126
2019	2431.772908	59.2090568
2020	2432.976843	59.1969108
2021	2434.181342	59.1837348
2022	2435.38635	59.1699333
2023	2436.591532	59.1551399
2024	2437.796536	59.1405563
2025	2439.001617	59.1259231
2026	2440.206049	59.1110839
2027	2441.411181	59.0964431
2028	2442.61586	59.0822219
2029	2443.821479	59.0669174
2030	2445.025671	59.051445
2031	2446.230632	59.0364608
2032	2447.43534	59.0217704
2033	2448.640065	59.0072135
2034	2449.845426	58.992073
2035	2451.050431	58.9771003
2036	2452.254544	58.9617919
2037	2453.458731	58.9467239
2038	2454.66365	58.9314384
2039	2455.868731	58.9173507
2040	2457.073395	58.9029159
2041	2458.276783	58.8882026
2042	2459.480839	58.8730697
2043	2460.685478	58.8586235
2044	2461.890175	58.8442268
2045	2463.094425	58.8288345
2046	2464.299471	58.8141059
2047	2465.506174	58.7991523
2048	2466.710793	58.7838668
2049	2467.91464	58.7691535

2050	2469.119691	58.7538337
2051	2470.32528	58.7380294
2052	2471.529926	58.7226638
2053	2472.735863	58.7073249
2054	2473.940814	58.691883
2055	2475.146327	58.6769332
2056	2476.350603	58.6620712
2057	2477.554962	58.6471786
2058	2478.759253	58.632183
2059	2479.963462	58.6166267
2060	2481.167017	58.6022872
2061	2482.371031	58.5882453
2062	2483.57569	58.5741081
2063	2484.780129	58.5592117
2064	2485.98438	58.544281
2065	2487.189039	58.5285949
2066	2488.393436	58.5125045
2067	2489.598664	58.4965515
2068	2490.803187	58.480957
2069	2492.008286	58.4649772
2070	2493.212772	58.4484443
2071	2494.41718	58.4319152
2072	2495.621753	58.4156265
2073	2496.82688	58.3992919
2074	2498.031898	58.3836555
2075	2499.235159	58.3681945
2076	2500.439087	58.3525314
2077	2501.643922	58.3362045
2078	2502.847812	58.3194694
2079	2504.05261	58.304161
2080	2505.257335	58.2886123
2081	2506.461184	58.272499
2082	2507.665408	58.2558097
2083	2508.869335	58.2389907
2084	2510.073789	58.2219467
2085	2511.278362	58.2048225
2086	2512.482803	58.1878585
2087	2513.686669	58.1717605
2088	2514.890737	58.1553764
2089	2516.096061	58.1384773
2090	2517.300333	58.1217269
2091	2518.505596	58.1049118
2092	2519.709838	58.0883064
2093	2520.913972	58.0728225
2094	2522.118865	58.057579
2095	2523.323824	58.0424728
2096	2524.528503	58.0272521
2097	2525.733387	58.0119705
2098	2526.937206	57.9971694
2099	2528.142352	57.9811935

2100	2529.347456	57.9654617
2101	2530.552325	57.9504585
2102	2531.756985	57.9352531
2103	2532.960843	57.9190254
2104	2534.165726	57.9027824
2105	2535.370153	57.8868942
2106	2536.575346	57.8714675
2107	2537.779954	57.8560981
2108	2538.98444	57.8400039
2109	2540.188685	57.8241767
2110	2541.392744	57.8081245
2111	2542.597228	57.7919311
2112	2543.802838	57.7755622
2113	2545.007948	57.760292
2114	2546.211124	57.7446365
2115	2547.415426	57.7284965
2116	2548.620038	57.7123489
2117	2549.825026	57.6956176
2118	2551.029353	57.6793823
2119	2552.233788	57.6630477
2120	2553.438762	57.6469612
2121	2554.642285	57.6306533
2122	2555.846841	57.6149177
2123	2557.052226	57.5977897
2124	2558.25746	57.5803337
2125	2559.462084	57.5632133
2126	2560.665406	57.5461006
2127	2561.872396	57.5291633
2128	2563.075931	57.5115966
2129	2564.280935	57.4947929
2130	2565.485331	57.4777107
2131	2566.689119	57.4608688
2132	2567.892657	57.4437789
2133	2569.097518	57.4271011
2134	2570.301534	57.4109115
2135	2571.506298	57.3944816
2136	2572.711541	57.3780212
2137	2573.915627	57.3605766
2138	2575.120284	57.3437271
2139	2576.324927	57.326477
2140	2577.52862	57.3092727
2141	2578.734424	57.2919425
2142	2579.938397	57.2740058
2143	2581.142749	57.2567977
2144	2582.347499	57.2389831
2145	2583.553233	57.2212295
2146	2584.758565	57.2033576
2147	2585.963875	57.1859817
2148	2587.168776	57.1685295
2149	2588.372662	57.150444

2150	2589.578148	57.1326179
2151	2590.783882	57.1146354
2152	2591.988933	57.0974731
2153	2593.19407	57.0794486
2154	2594.398682	57.0613708
2155	2595.602654	57.0439643
2156	2596.806909	57.026268
2157	2598.01205	57.0090713
2158	2599.216537	56.9918937
2159	2600.420897	56.9750671
2160	2601.625266	56.9582405
2161	2602.829899	56.9413528
2162	2604.034981	56.9235115
2163	2605.238773	56.9060134
2164	2606.443156	56.8894119
2165	2607.647237	56.8721542
2166	2608.85199	56.8545913
2167	2610.056848	56.8366775
2168	2611.261842	56.8187675
2169	2612.466716	56.8006782
2170	2613.670057	56.7831115
2171	2614.874889	56.7644882
2172	2616.079295	56.7472305
2173	2617.283674	56.7294006
2174	2618.487958	56.7117919
2175	2619.691957	56.6935958
2176	2620.8969	56.6763916
2177	2622.100555	56.6591186
2178	2623.305175	56.6423149
2179	2624.51039	56.6251754
2180	2625.714887	56.607563
2181	2626.91999	56.5905952
2182	2628.124556	56.5731811
2183	2629.328807	56.5564498
2184	2630.533686	56.5390014
2185	2631.738337	56.5223083
2186	2632.94254	56.5050125
2187	2634.146089	56.4878311
2188	2635.350458	56.4702262
2189	2636.554858	56.4531555
2190	2637.759222	56.4358329
2191	2638.964325	56.4189071
2192	2640.169413	56.401184
2193	2641.373774	56.3842468
2194	2642.578329	56.3670578
2195	2643.781938	56.349327
2196	2644.985702	56.3310012
2197	2646.190566	56.3133354
2198	2647.395756	56.2960128
2199	2648.599229	56.2783126

2200	2649.803154	56.2608146
2201	2651.006825	56.2428054
2202	2652.210808	56.2253723
2203	2653.414466	56.2064704
2204	2654.618389	56.1882019
2205	2655.822758	56.1701965
2206	2657.027534	56.152214
2207	2658.231918	56.1341056
2208	2659.435969	56.1157341
2209	2660.639728	56.097351
2210	2661.843612	56.0785102
2211	2663.047301	56.0598869
2212	2664.250883	56.040699
2213	2665.454961	56.0219345
2214	2666.658971	56.0030899
2215	2667.862637	55.9852409
2216	2669.066451	55.9670791
2217	2670.27016	55.9488029
2218	2671.474076	55.9304733
2219	2672.677788	55.9120101
2220	2673.881626	55.8935165
2221	2675.08541	55.8754196
2222	2676.289118	55.8579788
2223	2677.493189	55.8407821
2224	2678.696723	55.8233146
2225	2679.900546	55.804924
2226	2681.104321	55.7869453
2227	2682.308086	55.7689094
2228	2683.511831	55.7499198
2229	2684.715765	55.7325134
2230	2685.920037	55.7141304
2231	2687.125172	55.6956291
2232	2688.329556	55.6773796
2233	2689.533481	55.6586112
2234	2690.738063	55.6395378
2235	2691.943378	55.6211051
2236	2693.148539	55.6027641
2237	2694.353609	55.5841484
2238	2695.558005	55.5662765
2239	2696.762202	55.5472221
2240	2697.966778	55.5292892
2241	2699.171748	55.5120735
2242	2700.375669	55.4938659
2243	2701.580198	55.4755744
2244	2702.784751	55.4573898
2245	2703.989207	55.4396133
2246	2705.193646	55.4213638
2247	2706.398776	55.4041061
2248	2707.604024	55.3861274
2249	2708.807413	55.3684921

2250	2710.012458	55.3506698
2251	2711.217534	55.3327217
2252	2712.422039	55.314949
2253	2713.627	55.2974472
2254	2714.831911	55.279602
2255	2716.035634	55.2615585
2256	2717.240135	55.2435607
2257	2718.445393	55.2240715
2258	2719.650625	55.2058601
2259	2720.855499	55.1869583
2260	2722.060492	55.1687202
2261	2723.264956	55.149353
2262	2724.46991	55.1306762
2263	2725.674578	55.1123847
2264	2726.879585	55.0942001
2265	2728.085207	55.0754013
2266	2729.288859	55.0574264
2267	2730.492758	55.0402946
2268	2731.697368	55.0226135
2269	2732.902589	55.0059585
2270	2734.107145	54.9888992
2271	2735.311693	54.9720764
2272	2736.516492	54.9552993
2273	2737.721495	54.9382095
2274	2738.926228	54.9208488
2275	2740.130976	54.903553
2276	2741.335431	54.8859786
2277	2742.539483	54.8683357
2278	2743.743728	54.8508071
2279	2744.948283	54.8326644
2280	2746.153196	54.8144226
2281	2747.358812	54.795948
2282	2748.563472	54.7775077
2283	2749.767205	54.7583427
2284	2750.971475	54.7398681
2285	2752.176316	54.7214279
2286	2753.381233	54.7030258
2287	2754.584749	54.6845397
2288	2755.78946	54.6656723
2289	2756.994374	54.6467285
2290	2758.198765	54.6283073
2291	2759.403619	54.6106491
2292	2760.608626	54.5922622
2293	2761.813287	54.5751266
2294	2763.018924	54.5577926
2295	2764.224866	54.5405464
2296	2765.429743	54.5230712
2297	2766.635114	54.5053405
2298	2767.840403	54.4879379
2299	2769.045214	54.470375

2300	2770.24894	54.4528503
2301	2771.453213	54.4349784
2302	2772.658463	54.4172592
2303	2773.86185	54.3989181
2304	2775.065868	54.3802604
2305	2776.270228	54.3614883
2306	2777.474393	54.3427047
2307	2778.677655	54.3248786
2308	2779.88247	54.3070907
2309	2781.087156	54.2898368
2310	2782.291789	54.2713012
2311	2783.496845	54.2531967
2312	2784.700962	54.235321
2313	2785.905872	54.2182388
2314	2787.111333	54.2012825
2315	2788.316914	54.183876
2316	2789.522236	54.1667861
2317	2790.726533	54.1483535
2318	2791.93162	54.1302185
2319	2793.135846	54.1112594
2320	2794.341528	54.0935668
2321	2795.546672	54.0752868
2322	2796.750848	54.0568771
2323	2797.954818	54.0382614
2324	2799.158832	54.0194816
2325	2800.363317	54.0011825
2326	2801.56864	53.9827079
2327	2802.77339	53.9655876
2328	2803.97786	53.947998
2329	2805.187209	53.9307174
2330	2806.392236	53.9131965
2331	2807.597166	53.8956527
2332	2808.80176	53.8783645
2333	2810.043643	53.8611984
2334	2811.248037	53.8447952
2335	2812.452782	53.8275566
2336	2813.657701	53.810688
2337	2814.862273	53.7931213
2338	2816.066929	53.7758064
2339	2817.270916	53.7588577
2340	2818.475192	53.7417373
2341	2819.678979	53.7250862
2342	2820.883933	53.7082138
2343	2822.088776	53.690998
2344	2823.292841	53.6734428
2345	2824.496991	53.6565361
2346	2825.701818	53.6392517
2347	2826.90706	53.6220474
2348	2828.111856	53.6039314
2349	2829.316729	53.5857658

2350	2830.521748	53.5674209
2351	2831.725115	53.5495605
2352	2832.929679	53.5313835
2353	2834.13498	53.5132865
2354	2835.339621	53.4950256
2355	2836.543792	53.4767036
2356	2837.74801	53.4579772
2357	2838.952337	53.4393081
2358	2840.156546	53.4216918
2359	2841.360587	53.4044799
2360	2842.56524	53.3874626
2361	2843.77031	53.3698043
2362	2844.973547	53.3531875
2363	2846.177608	53.3364334
2364	2847.382686	53.3199386
2365	2848.588023	53.3040046
2366	2849.792432	53.288166
2367	2850.996516	53.2729072
2368	2852.200316	53.2571792
2369	2853.404961	53.2412643
2370	2854.609553	53.2254333
2371	2855.814174	53.2096366
2372	2857.018561	53.1929817
2373	2858.222752	53.1768112
2374	2859.427604	53.1604232
2375	2860.633202	53.1435432
2376	2861.837988	53.12788
2377	2863.042792	53.1114578
2378	2864.246906	53.0945053
2379	2865.450561	53.0776977
2380	2866.655275	53.0608139
2381	2867.859705	53.0439376
2382	2869.065151	53.0271606
2383	2870.269526	53.0103569
2384	2871.472896	52.9934577
2385	2872.677344	52.9767951
2386	2873.882107	52.9598579
2387	2875.087484	52.9428901
2388	2876.292592	52.9271049
2389	2877.497959	52.9108505
2390	2878.701687	52.8950424
2391	2879.906988	52.8791198
2392	2881.111358	52.8633499
2393	2882.316363	52.8469924
2394	2883.521535	52.8319778
2395	2884.725627	52.8161849
2396	2885.930198	52.7999191
2397	2887.134406	52.783184
2398	2888.339798	52.7662963
2399	2889.545467	52.749916

2400	2890.750279	52.7331123
2401	2891.954879	52.7160148
2402	2893.159842	52.6987113
2403	2894.364684	52.6827163
2404	2895.569405	52.6655197
2405	2896.773954	52.6486434
2406	2897.978483	52.632122
2407	2899.182335	52.6165161
2408	2900.387343	52.6007003
2409	2901.591812	52.5853958
2410	2902.796648	52.5695877
2411	2904.000672	52.5544204
2412	2905.204017	52.5391311
2413	2906.409014	52.5230522
2414	2907.613504	52.5078506
2415	2908.8187	52.4923286
2416	2910.023426	52.4776496
2417	2911.227068	52.4629936
2418	2912.431474	52.4479751
2419	2913.636801	52.4322586
2420	2914.841391	52.4179458
2421	2916.046552	52.4032478
2422	2917.250388	52.3888168
2423	2918.455016	52.374298
2424	2919.659863	52.3586311
2425	2920.863975	52.3436355
2426	2922.06882	52.3276138
2427	2923.274026	52.3115158
2428	2924.478708	52.29496
2429	2925.682874	52.278759
2430	2926.887205	52.2613906
2431	2928.092814	52.2442512
2432	2929.29758	52.2269973
2433	2930.501168	52.2099494
2434	2931.70487	52.1934623
2435	2932.908855	52.1771621
2436	2934.112656	52.1610107
2437	2935.317323	52.1452713
2438	2936.522723	52.1299819
2439	2937.727173	52.1142692
2440	2938.93204	52.0989303
2441	2940.137712	52.083435
2442	2941.342913	52.067913
2443	2942.548076	52.0518493
2444	2943.752911	52.0358695
2445	2944.957472	52.0196723
2446	2946.161173	52.0034484
2447	2947.365833	51.9859924
2448	2948.57222	51.969139
2449	2949.776874	51.9525527

2450	2950.981313	51.9360542
2451	2952.185229	51.9198722
2452	2953.390161	51.9047851
2453	2954.593886	51.8901443
2454	2955.797697	51.8756027
2455	2957.001431	51.8615188
2456	2958.205239	51.8470458
2457	2959.409069	51.8341064
2458	2960.613661	51.821186
2459	2961.818107	51.8075942
2460	2963.023121	51.7939033
2461	2964.227649	51.7796096
2462	2965.432584	51.7651252
2463	2966.636764	51.7497024
2464	2967.840734	51.7339401
2465	2969.045505	51.718151
2466	2970.24934	51.7032699
2467	2971.453545	51.6873054
2468	2972.657595	51.6708564
2469	2973.861497	51.6547164
2470	2975.065817	51.6397018
2471	2976.270714	51.6251945
2472	2977.475491	51.6102218
2473	2978.680321	51.5962524
2474	2979.884192	51.5825309
2475	2981.089026	51.5692024
2476	2982.294139	51.5549087
2477	2983.498799	51.5400886
2478	2984.704326	51.5263366
2479	2985.909017	51.5126533
2480	2987.113221	51.4985656
2481	2988.318957	51.484169
2482	2989.523869	51.4701156
2483	2990.729574	51.4558334
2484	2991.934315	51.4416007
2485	2993.140482	51.426342
2486	2994.344476	51.4113693
2487	2995.549414	51.3971023
2488	2996.753725	51.382225
2489	2997.957667	51.3671646
2490	2999.161855	51.3520507
2491	3000.365972	51.3368949
2492	3001.569785	51.3212966
2493	3002.774886	51.3057022
2494	3003.980044	51.2904243
2495	3005.185252	51.2754592
2496	3006.389917	51.2605667
2497	3007.594019	51.2463912
2498	3008.798815	51.2321052
2499	3010.004107	51.2188529

2500	3011.208663	51.2048034
2501	3012.412665	51.1910591
2502	3013.616804	51.1776237
2503	3014.821179	51.1644477
2504	3016.026885	51.1509361
2505	3017.232525	51.137886
2506	3018.436937	51.1248245
2507	3019.641236	51.1108093
2508	3020.845489	51.0967788
2509	3022.049666	51.0818405
2510	3023.254453	51.0675048
2511	3024.460164	51.05373
2512	3025.664904	51.0399513
2513	3026.869019	51.0255622
2514	3028.073441	51.011177
2515	3029.278493	50.9965133
2516	3030.483042	50.9822502
2517	3031.687738	50.9678115
2518	3032.892417	50.9538192
2519	3034.096322	50.9404029
2520	3035.299933	50.9266166
2521	3036.504473	50.9121704
2522	3037.709677	50.8969192
2523	3038.915234	50.8826026
2524	3040.119638	50.86837
2525	3041.323916	50.8542976
2526	3042.529128	50.8398933
2527	3043.734111	50.8267745
2528	3044.939044	50.8135299
2529	3046.143923	50.7998275
2530	3047.350144	50.7856178
2531	3048.55438	50.7722244
2532	3049.759332	50.7591819
2533	3050.96387	50.7461624
2534	3052.168381	50.7329826
2535	3053.371456	50.7199935
2536	3054.576597	50.7069702
2537	3055.781128	50.6935806
2538	3056.985954	50.6803169
2539	3058.191048	50.6668014
2540	3059.395539	50.654335
2541	3060.599704	50.6414451
2542	3061.80504	50.6291084
2543	3063.009901	50.6159591
2544	3064.214172	50.603733
2545	3065.418591	50.5903854
2546	3066.623545	50.577095
2547	3067.827283	50.5640792
2548	3069.032095	50.550354
2549	3070.237025	50.5367622

2550	3071.441883	50.5232009
2551	3072.646337	50.509552
2552	3073.850409	50.4958686
2553	3075.053798	50.4822502
2554	3076.258475	50.467453
2555	3077.463605	50.4532623
2556	3078.668965	50.4397163
2557	3079.873641	50.4259757
2558	3081.078394	50.4122352
2559	3082.28378	50.3993606
2560	3083.488769	50.386959
2561	3084.69451	50.3748626
2562	3085.899509	50.3629341
2563	3087.104712	50.3516082
2564	3088.309838	50.3408432
2565	3089.515212	50.3301696
2566	3090.719896	50.3192634
2567	3091.924977	50.3079376
2568	3093.1294	50.2971229
2569	3094.332555	50.2863159
2570	3095.538405	50.2742691
2571	3096.742503	50.2622909
2572	3097.947927	50.2498893
2573	3099.152904	50.2377471
2574	3100.357133	50.2255439
2575	3101.560807	50.2136993
2576	3102.765027	50.2019805
2577	3103.968823	50.1903152
2578	3105.173551	50.1776657
2579	3106.378409	50.1648178
2580	3107.582629	50.152523
2581	3108.787195	50.138916
2582	3109.99144	50.1256027
2583	3111.196049	50.1118621
2584	3112.400801	50.0984764
2585	3113.605358	50.085186
2586	3114.809977	50.0717544
2587	3116.01469	50.0579833
2588	3117.21888	50.0450592
2589	3118.424349	50.0323486
2590	3119.628595	50.0193557
2591	3120.832537	50.0066299
2592	3122.036979	49.9938812
2593	3123.241797	49.9815063
2594	3124.446996	49.9691963
2595	3125.652044	49.9555549
2596	3126.857088	49.9419479
2597	3128.060805	49.9294929
2598	3129.264595	49.9176139
2599	3130.469908	49.9046707

2600	3131.673709	49.8925781
2601	3132.878829	49.8811187
2602	3134.083223	49.8695602
2603	3135.288511	49.8578758
2604	3136.4923	49.8459434
2605	3137.69676	49.8358078
2606	3138.902289	49.8248519
2607	3140.106811	49.8135604
2608	3141.311341	49.8025169
2609	3142.516398	49.7917251
2610	3143.721049	49.7806625
2611	3144.925742	49.7699775
2612	3146.130419	49.7597427
2613	3147.334961	49.748455
2614	3148.53963	49.7375564
2615	3149.744464	49.7259902
2616	3150.949099	49.7159156
2617	3152.152844	49.7052078
2618	3153.358245	49.6943359
2619	3154.561663	49.683815
2620	3155.76684	49.6730842
2621	3156.970791	49.6628189
2622	3158.175631	49.6515579
2623	3159.381034	49.6410293
2624	3160.584653	49.6298713
2625	3161.789203	49.6187934
2626	3162.99303	49.6065826
2627	3164.196547	49.5943717
2628	3165.400497	49.5823364
2629	3166.604722	49.5698127
2630	3167.80859	49.5579299
2631	3169.012977	49.5455055
2632	3170.217501	49.5335235
2633	3171.422147	49.5218124
2634	3172.62632	49.5100021
2635	3173.831543	49.497734
2636	3175.036419	49.4861106
2637	3176.240277	49.4752693
2638	3177.444244	49.4633407
2639	3178.648059	49.4523849
2640	3179.851585	49.4399108
2641	3181.056816	49.4285354
2642	3182.261218	49.4165802
2643	3183.465398	49.4050407
2644	3184.669486	49.3931045
2645	3185.874061	49.3820228
2646	3187.078172	49.3696784
2647	3188.281806	49.3574333
2648	3189.485983	49.345581
2649	3190.690774	49.3335266

2650	3191.8949	49.3225097
2651	3193.098762	49.3105354
2652	3194.303099	49.2993659
2653	3195.507608	49.2878494
2654	3196.712122	49.2769432
2655	3197.917693	49.2651901
2656	3199.122683	49.2543182
2657	3200.327344	49.2436408
2658	3201.531193	49.2328338
2659	3202.735135	49.2209892
2660	3203.940586	49.209259
2661	3205.145047	49.1970596
2662	3206.349035	49.1845092
2663	3207.553619	49.1718673
2664	3208.758052	49.1590194
2665	3209.962174	49.1466979
2666	3211.166285	49.1349716
2667	3212.370978	49.1224746
2668	3213.575285	49.1106262
2669	3214.780383	49.0996665
2670	3215.98519	49.0889244
2671	3217.190441	49.0783271
2672	3218.394677	49.068264
2673	3219.598498	49.0584983
2674	3220.803885	49.0491676
2675	3222.00722	49.0401344
2676	3223.210833	49.0300331
2677	3224.415113	49.019947
2678	3225.619913	49.0100593
2679	3226.825397	48.9999504
2680	3228.030185	48.9892158
2681	3229.234684	48.9789428
2682	3230.439559	48.9679679
2683	3231.644528	48.9575996
2684	3232.848492	48.9472236
2685	3234.052382	48.9368972
2686	3235.257252	48.9278984
2687	3236.462141	48.9191131
2688	3237.66726	48.9102859
2689	3238.872243	48.9017982
2690	3240.07675	48.8944664
2691	3241.281194	48.8871231
2692	3242.485592	48.8807449
2693	3243.68928	48.8736457
2694	3244.894058	48.8666801
2695	3246.099418	48.860157
2696	3247.304562	48.852951
2697	3248.508485	48.845932
2698	3249.71269	48.8397254
2699	3250.917443	48.8337326

2700	3252.122659	48.827053
2701	3253.327788	48.8203353
2702	3254.531636	48.8144607
2703	3255.736205	48.8090744
2704	3256.940875	48.8038787
2705	3258.144334	48.7990646
2706	3259.348329	48.7945823
2707	3260.55207	48.7904129
2708	3261.755732	48.786518
2709	3262.959744	48.7829017
2710	3264.163454	48.7799377
2711	3265.367223	48.7772636
2712	3266.572536	48.7744789
2713	3267.777157	48.7723388
2714	3268.981537	48.7712287
2715	3270.185746	48.7697067
2716	3271.390822	48.7692413
2717	3272.596211	48.7693252
2718	3273.801103	48.7690811
2719	3275.005822	48.7696113
2720	3276.210172	48.7706909
2721	3277.41344	48.7716445
2722	3278.618392	48.7728195
2723	3279.822995	48.7742691
2724	3281.027996	48.7753105
2725	3282.232698	48.7762527
2726	3283.435956	48.7772483
2727	3284.640526	48.7787094
2728	3285.845348	48.7799606
2729	3287.050656	48.780651
2730	3288.255446	48.7820663
2731	3289.458659	48.7834091
2732	3290.663708	48.7848014
2733	3291.868222	48.7857627
2734	3293.072858	48.7867927
2735	3294.277517	48.7888755
2736	3295.481291	48.7907714
2737	3296.68555	48.7925949
2738	3297.889793	48.7946205
2739	3299.093142	48.797264
2740	3300.298326	48.7984733
2741	3301.502853	48.8007583
2742	3302.707391	48.8027343
2743	3303.911393	48.8047561
2744	3305.116109	48.8077392
2745	3306.320981	48.8094139
2746	3307.525859	48.8111991
2747	3308.73147	48.813446
2748	3309.936766	48.8160324
2749	3311.141735	48.8183517

2750	3312.345687	48.8210411
2751	3313.55072	48.8234939
2752	3314.755739	48.8265762
2753	3315.960702	48.8292045
2754	3317.164591	48.8306961
2755	3318.369455	48.8329124
2756	3319.575218	48.83535
2757	3320.779947	48.8364791
2758	3321.984412	48.8370895
2759	3323.188541	48.8376235
2760	3324.393764	48.8379745
2761	3325.598486	48.8387832
2762	3326.803354	48.8387527
2763	3328.007998	48.838665
2764	3329.212626	48.8384399
2765	3330.416643	48.8386535
2766	3331.621843	48.8382644
2767	3332.826641	48.8389358
2768	3334.031969	48.8394546
2769	3335.237217	48.8399391
2770	3336.441667	48.8401336
2771	3337.645577	48.8399353
2772	3338.849501	48.8405227
2773	3340.054608	48.8409309
2774	3341.259938	48.8419609
2775	3342.464144	48.8420639
2776	3343.668285	48.8421859
2777	3344.872945	48.8419761
2778	3346.07822	48.8416671
2779	3347.282578	48.8413314
2780	3348.487849	48.8415298
2781	3349.692038	48.8411369
2782	3350.896502	48.8401756
2783	3352.10148	48.8392677
2784	3353.305825	48.838417
2785	3354.510032	48.8365592
2786	3355.714663	48.8346519
2787	3356.918184	48.8323135
2788	3358.122726	48.829998
2789	3359.326509	48.8264541
2790	3360.530625	48.823738
2791	3361.734678	48.8202972
2792	3362.939853	48.8171157
2793	3364.144323	48.8142776
2794	3365.348483	48.8107948
2795	3366.552997	48.808319
2796	3367.757638	48.8058204
2797	3368.962214	48.8030509
2798	3370.167181	48.8004264
2799	3371.37127	48.7989578

2800	3372.575642	48.7961387
2801	3373.780363	48.7940254
2802	3374.985334	48.7910385
2803	3376.189725	48.7877311
2804	3377.394648	48.7850761
2805	3378.599055	48.7830123
2806	3379.803818	48.7797737
2807	3381.007997	48.7765083
2808	3382.213011	48.773014
2809	3383.417747	48.7688789
2810	3384.621884	48.7649574
2811	3385.826756	48.7609977
2812	3387.031793	48.7577629
2813	3388.236589	48.7544975
2814	3389.441288	48.7503738
2815	3390.6449	48.7457885
2816	3391.849192	48.7420043
2817	3393.054544	48.7381439
2818	3394.259047	48.7343788
2819	3395.463209	48.7314529
2820	3396.667163	48.7278633
2821	3397.871867	48.7242393
2822	3399.076153	48.7201614
2823	3400.280689	48.7157363
2824	3401.485124	48.7107276
2825	3402.689872	48.7058067
2826	3403.892967	48.7002449
2827	3405.097888	48.6951866
2828	3406.302256	48.6896781
2829	3407.506009	48.6841087
2830	3408.710745	48.6786346
2831	3409.915809	48.6735229
2832	3411.119946	48.6685981
2833	3412.323837	48.6637725
2834	3413.528916	48.6596374
2835	3414.733995	48.6548423
2836	3415.937325	48.6503639
2837	3417.14137	48.6462059
2838	3418.3459	48.6421928
2839	3419.550845	48.6370086
2840	3420.755698	48.6323623
2841	3421.960422	48.6270561
2842	3423.164703	48.6216812
2843	3424.369255	48.6164436
2844	3425.573149	48.6107482
2845	3426.778208	48.6056861
2846	3427.982856	48.6011085
2847	3429.187429	48.5953674
2848	3430.391332	48.5900917
2849	3431.596154	48.5852584

2850	3432.800107	48.5807876
2851	3434.004599	48.5761222
2852	3435.209607	48.5715446
2853	3436.414546	48.5666923
2854	3437.618884	48.5624427
2855	3438.822958	48.5573463
2856	3440.026279	48.5525703
2857	3441.23049	48.5476989
2858	3442.434498	48.5420227
2859	3443.638555	48.5356674
2860	3444.842672	48.5292129
2861	3446.047795	48.5226211
2862	3447.252293	48.515892
2863	3448.457846	48.5098075
2864	3449.662918	48.502922
2865	3450.867408	48.4964332
2866	3452.072074	48.4891357
2867	3453.276423	48.4820938
2868	3454.480887	48.4756507
2869	3455.685886	48.4699363
2870	3456.890666	48.4638442
2871	3458.095272	48.4580917
2872	3459.299196	48.4519424
2873	3460.503814	48.4448699
2874	3461.708255	48.4383239
2875	3462.913054	48.4320449
2876	3464.117073	48.4267158
2877	3465.32122	48.421154
2878	3466.524836	48.4158248
2879	3467.728744	48.4098777
2880	3468.933264	48.4042091
2881	3470.137779	48.3987998
2882	3471.342928	48.3934249
2883	3472.547556	48.3884201
2884	3473.752125	48.3835144
2885	3474.957497	48.3778343
2886	3476.162172	48.3719253
2887	3477.366177	48.3652458
2888	3478.570346	48.3585433
2889	3479.77519	48.3526687
2890	3480.980534	48.3458366
2891	3482.185251	48.3387222
2892	3483.389847	48.3319053
2893	3484.593212	48.325386
2894	3485.797858	48.3185958
2895	3487.001584	48.3129692
2896	3488.206159	48.3062629
2897	3489.412027	48.3012771
2898	3490.6166	48.2950019
2899	3491.820808	48.2885818

2900	3493.024692	48.2830085
2901	3494.229899	48.2778778
2902	3495.433811	48.2718429
2903	3496.639464	48.2667274
2904	3497.84437	48.2612686
2905	3499.048278	48.2548637
2906	3500.252847	48.2486801
2907	3501.458268	48.2421798
2908	3502.662218	48.2364807
2909	3503.867086	48.2300796
2910	3505.071621	48.2239074
2911	3506.275871	48.2171249
2912	3507.480395	48.2107391
2913	3508.686056	48.2038192
2914	3509.890827	48.1978149
2915	3511.095236	48.1924476
2916	3512.299018	48.1865921
2917	3513.502745	48.1808319
2918	3514.706775	48.1747055
2919	3515.910916	48.1686134
2920	3517.115531	48.1617698
2921	3518.320043	48.1554832
2922	3519.524946	48.1495513
2923	3520.730127	48.1428489
2924	3521.934583	48.1359939
2925	3523.140091	48.1280708
2926	3524.344259	48.1215896
2927	3525.548183	48.1140632
2928	3526.753608	48.1069183
2929	3527.958013	48.1007461
2930	3529.162918	48.0943183
2931	3530.367927	48.0876998
2932	3531.57214	48.081295
2933	3532.777273	48.0748901
2934	3533.982428	48.0684356
2935	3535.187204	48.0622596
2936	3536.391595	48.0552902
2937	3537.596128	48.0491828
2938	3538.800507	48.0427322
2939	3540.00459	48.0359992
2940	3541.210306	48.0290412
2941	3542.415509	48.0220718
2942	3543.620994	48.0148735
2943	3544.824844	48.0074882
2944	3546.028411	47.9997024
2945	3547.234265	47.9923477
2946	3548.438107	47.9853897
2947	3549.64271	47.9783401
2948	3550.847685	47.9711914
2949	3552.052684	47.9634628

2950	3553.256927	47.9565811
2951	3554.461739	47.9497222
2952	3555.666832	47.9426155
2953	3556.872209	47.9357566
2954	3558.077574	47.9290657
2955	3559.282063	47.9220619
2956	3560.485963	47.9145927
2957	3561.68987	47.9070968
2958	3562.893674	47.8998718
2959	3564.097391	47.8933372
2960	3565.301112	47.8863754
2961	3566.504927	47.8797836
2962	3567.708858	47.8731498
2963	3568.912575	47.8662719
2964	3570.116306	47.8588943
2965	3571.32004	47.8523521
2966	3572.524194	47.8457145
2967	3573.727826	47.8386306
2968	3574.9316	47.8318939
2969	3576.135165	47.8247833
2970	3577.338936	47.8173141
2971	3578.543157	47.8096389
2972	3579.746937	47.8024368
2973	3580.951505	47.7951431
2974	3582.156156	47.7880325
2975	3583.359921	47.7806243
2976	3584.56386	47.7728042
2977	3585.767515	47.7658195
2978	3586.971549	47.7582092
2979	3588.176431	47.7498741
2980	3589.381364	47.7424621
2981	3590.586353	47.7343864
2982	3591.790656	47.7258377
2983	3592.997752	47.7182083
2984	3594.201808	47.7107772
2985	3595.407553	47.7028923
2986	3596.613227	47.6958198
2987	3597.817587	47.6879997
2988	3599.022104	47.6803169
2989	3600.225282	47.6740379
2990	3601.429077	47.6673622
2991	3602.633437	47.6608352
2992	3603.838477	47.6540603
2993	3605.04347	47.6463623
2994	3606.247675	47.6387329
2995	3607.451227	47.6305427
2996	3608.655657	47.6225662
2997	3609.86103	47.6149902
2998	3611.065957	47.6070365
2999	3612.271672	47.5983963

3000	3613.475944	47.5897674
3001	3614.679132	47.5819587
3002	3615.883904	47.5740814
3003	3617.08937	47.5667762
3004	3618.29371	47.5587692
3005	3619.498279	47.5522651
3006	3620.701835	47.5448684
3007	3621.907118	47.536663
3008	3623.112187	47.5291976
3009	3624.316979	47.5219955
3010	3625.522055	47.514656
3011	3626.727282	47.5067291
3012	3627.932642	47.4991569
3013	3629.136632	47.4912986
3014	3630.341597	47.4836921
3015	3631.546156	47.4755935
3016	3632.751353	47.4681167
3017	3633.95531	47.4610824
3018	3635.160345	47.4533386
3019	3636.36488	47.4449653
3020	3637.570135	47.437004
3021	3638.774662	47.4287567
3022	3639.97896	47.4202995
3023	3641.182532	47.4116096
3024	3642.387487	47.4033317
3025	3643.591904	47.3942985
3026	3644.796114	47.3852348
3027	3646.000744	47.3767509
3028	3647.205565	47.368309
3029	3648.409716	47.3605461
3030	3649.614072	47.3519363
3031	3650.819257	47.343563
3032	3652.023955	47.3358345
3033	3653.228131	47.328041
3034	3654.431367	47.3200149
3035	3655.638622	47.3126564
3036	3656.8431	47.3044509
3037	3658.047985	47.2962455
3038	3659.252815	47.2896194
3039	3660.456582	47.2823715
3040	3661.660454	47.2756996
3041	3662.866315	47.2690353
3042	3664.071563	47.2621879
3043	3665.275794	47.2553062
3044	3666.480578	47.2484207
3045	3667.685633	47.240921
3046	3668.889122	47.2339668
3047	3670.093202	47.2265205
3048	3671.297287	47.217205
3049	3672.502022	47.2075576

3050	3673.705116	47.1980705
3051	3674.909184	47.18787
3052	3676.113488	47.1777343
3053	3677.318902	47.168003
3054	3678.523611	47.1585731
3055	3679.72842	47.1498603
3056	3680.93185	47.1413955
3057	3682.135666	47.1322746
3058	3683.34101	47.1241149
3059	3684.546184	47.1167297
3060	3685.751909	47.1085739
3061	3686.957155	47.1009902
3062	3688.160799	47.0933494
3063	3689.365352	47.0856246
3064	3690.570112	47.0773696
3065	3691.773877	47.0686492
3066	3692.977945	47.059658
3067	3694.181812	47.0508117
3068	3695.385458	47.0418586
3069	3696.590411	47.0329627
3070	3697.796163	47.0238265
3071	3699.000762	47.0152397
3072	3700.20547	47.0059852
3073	3701.409773	46.9970169
3074	3702.61416	46.9881172
3075	3703.817641	46.9800796
3076	3705.022051	46.9717292
3077	3706.227266	46.9641723
3078	3707.430925	46.9568634
3079	3708.634609	46.9490127
3080	3709.839306	46.9408607
3081	3711.043672	46.9329452
3082	3712.248593	46.9248809
3083	3713.453031	46.91716
3084	3714.659178	46.9093437
3085	3715.863466	46.900753
3086	3717.067931	46.8923988
3087	3718.273146	46.8836708
3088	3719.477594	46.8751144
3089	3720.681387	46.8665237
3090	3721.886115	46.8585548
3091	3723.090119	46.850151
3092	3724.294776	46.8419303
3093	3725.498839	46.8332328
3094	3726.702878	46.8248977
3095	3727.908152	46.8166542
3096	3729.112623	46.8086929
3097	3730.316862	46.8006668
3098	3731.521193	46.7915573
3099	3732.726221	46.7827835

3100	3733.929731	46.7742424
3101	3735.133894	46.7656135
3102	3736.338534	46.7571983
3103	3737.543531	46.7480812
3104	3738.748066	46.7385025
3105	3739.9535	46.7290878
3106	3741.157699	46.7193374
3107	3742.361069	46.7100524
3108	3743.566169	46.7010459
3109	3744.770942	46.6921195
3110	3745.976551	46.6837844
3111	3747.180188	46.6754608
3112	3748.385018	46.6669692
3113	3749.588396	46.6589736
3114	3750.793432	46.6507186
3115	3751.998212	46.6423873
3116	3753.20364	46.6343383
3117	3754.408633	46.6261825
3118	3755.613265	46.617897
3119	3756.81768	46.6099166
3120	3758.022358	46.6003952
3121	3759.227663	46.5901412
3122	3760.432318	46.5807723
3123	3761.635913	46.5717544
3124	3762.839671	46.5630302
3125	3764.044503	46.5537261
3126	3765.248941	46.544342
3127	3766.453434	46.535942
3128	3767.657209	46.5274391
3129	3768.861921	46.5186386
3130	3770.066933	46.5108032
3131	3771.271014	46.5034942
3132	3772.475481	46.4954338
3133	3773.6805	46.4873619
3134	3774.884717	46.4798851
3135	3776.088342	46.4726562
3136	3777.293265	46.4660301
3137	3778.497794	46.4578704
3138	3779.70253	46.4493179
3139	3780.907177	46.4407882
3140	3782.110484	46.4320907
3141	3783.314436	46.4239387
3142	3784.519693	46.4165115
3143	3785.723841	46.4080467
3144	3786.927434	46.3997421
3145	3788.131935	46.390995
3146	3789.336279	46.3815536
3147	3790.539902	46.3724403
3148	3791.743946	46.3640022
3149	3792.948841	46.3558616

3150	3794.153478	46.3479728
3151	3795.356932	46.339241
3152	3796.56113	46.3298416
3153	3797.76575	46.3212356
3154	3798.9705	46.3126029
3155	3800.175713	46.3040084
3156	3801.380073	46.296875
3157	3802.584309	46.2884635
3158	3803.789898	46.2806701
3159	3804.994965	46.2719535
3160	3806.199938	46.2637634
3161	3807.404412	46.2556915
3162	3808.609232	46.2479782
3163	3809.81369	46.2391891
3164	3811.018541	46.2308578
3165	3812.222629	46.2228469
3166	3813.426774	46.2127494
3167	3814.631067	46.2040901
3168	3815.835371	46.1946792
3169	3817.039552	46.1863098
3170	3818.244482	46.1770553
3171	3819.44883	46.1680374
3172	3820.653106	46.1588363
3173	3821.856813	46.1506652
3174	3823.060965	46.1414604
3175	3824.265957	46.1321411
3176	3825.470196	46.122734
3177	3826.673508	46.1131362
3178	3827.878131	46.1041755
3179	3829.082587	46.0951576
3180	3830.287462	46.0866127
3181	3831.492162	46.0782241
3182	3832.69791	46.0701789
3183	3833.902999	46.061737
3184	3835.107623	46.0537872
3185	3836.311665	46.0460777
3186	3837.516398	46.0392456
3187	3838.720465	46.0327262
3188	3839.925182	46.0255928
3189	3841.130092	46.0177078
3190	3842.334457	46.0092773
3191	3843.538654	46.0013046
3192	3844.743518	45.9929885
3193	3845.948082	45.9842453
3194	3847.152995	45.975708
3195	3848.357557	45.9666824
3196	3849.562382	45.9577255
3197	3850.765972	45.9483718
3198	3851.970847	45.9397315
3199	3853.175421	45.9298782

3200	3854.38095	45.9204483
3201	3855.584614	45.9109649
3202	3856.788608	45.9014549
3203	3857.992296	45.8923759
3204	3859.196697	45.882759
3205	3860.401359	45.8736648
3206	3861.606204	45.8644828
3207	3862.810656	45.8552284
3208	3864.014739	45.8460159
3209	3865.218662	45.8387184
3210	3866.423114	45.8320083
3211	3867.628056	45.8246345
3212	3868.832986	45.8168258
3213	3870.036905	45.8089981
3214	3871.24165	45.8012542
3215	3872.445351	45.7938079
3216	3873.649314	45.7859039
3217	3874.853006	45.7781448
3218	3876.056841	45.7703781
3219	3877.261215	45.7625503
3220	3878.464883	45.7538261
3221	3879.669967	45.7450141
3222	3880.875711	45.7363891
3223	3882.080216	45.7281608
3224	3883.28381	45.720169
3225	3884.487178	45.711956
3226	3885.691578	45.7045135
3227	3886.896286	45.697174
3228	3888.10105	45.6893692
3229	3889.305456	45.6812629
3230	3890.509418	45.6735191
3231	3891.714339	45.665306
3232	3892.919265	45.6578559
3233	3894.124297	45.6503295
3234	3895.328983	45.6428337
3235	3896.533581	45.6356391
3236	3897.737398	45.6277694
3237	3898.942106	45.6202735
3238	3900.14689	45.6126289
3239	3901.351835	45.6041336
3240	3902.557012	45.5967025
3241	3903.760891	45.5892715
3242	3904.965711	45.5817604
3243	3906.170082	45.5743522
3244	3907.374517	45.5660629
3245	3908.579666	45.5583229
3246	3909.784193	45.550476
3247	3910.988134	45.5417366
3248	3912.193274	45.5341415
3249	3913.398557	45.5270462

3250	3914.603276	45.5187759
3251	3915.807797	45.5108566
3252	3917.012809	45.5023803
3253	3918.21707	45.4938392
3254	3919.420682	45.4855957
3255	3920.625531	45.4766616
3256	3921.83063	45.468296
3257	3923.034187	45.4606513
3258	3924.237912	45.4519577
3259	3925.441904	45.4432029
3260	3926.646846	45.4345893
3261	3927.852266	45.426979
3262	3929.057374	45.4190292
3263	3930.261105	45.4112243
3264	3931.465726	45.403778
3265	3932.670404	45.3963928
3266	3933.874718	45.3887023
3267	3935.079301	45.3805885
3268	3936.284227	45.3734436
3269	3937.488427	45.3660469
3270	3938.692928	45.358982
3271	3939.897653	45.3509521
3272	3941.102538	45.3432846
3273	3942.307123	45.3354492
3274	3943.512211	45.3273696
3275	3944.716743	45.3193397
3276	3945.921007	45.3111648
3277	3947.125315	45.3036575
3278	3948.330006	45.2954826
3279	3949.534082	45.2879333
3280	3950.738576	45.2804374
3281	3951.942693	45.2737617
3282	3953.147383	45.2671432
3283	3954.352396	45.2612686
3284	3955.556352	45.2548675
3285	3956.761167	45.248455
3286	3957.965967	45.242485
3287	3959.169729	45.2358245
3288	3960.374957	45.228981
3289	3961.579879	45.2223434
3290	3962.784371	45.2152557
3291	3963.98829	45.2081832
3292	3965.192192	45.200283
3293	3966.396893	45.1916503
3294	3967.602009	45.1841583
3295	3968.807638	45.1758384
3296	3970.012203	45.1683235
3297	3971.216158	45.1616172
3298	3972.421473	45.1537361
3299	3973.626042	45.1454162

3300	3974.831066	45.1368408
3301	3976.035799	45.1270408
3302	3977.240188	45.1184005
3303	3978.445586	45.1096496
3304	3979.649232	45.1006889
3305	3980.853404	45.0923423
3306	3982.058833	45.0831604
3307	3983.264345	45.0742225
3308	3984.467622	45.0662231
3309	3985.672435	45.0588798
3310	3986.877194	45.0517921
3311	3988.082052	45.0456314
3312	3989.286	45.0391044
3313	3990.491294	45.0325965
3314	3991.695001	45.0260467
3315	3992.899987	45.0193901
3316	3994.104637	45.013031
3317	3995.309629	45.0054397
3318	3996.513782	44.9979553
3319	3997.717532	44.9895973
3320	3998.921585	44.9816665
3321	4000.126239	44.9738082
3322	4001.33114	44.9657173
3323	4002.535969	44.9575119
3324	4003.740862	44.949337
3325	4004.945466	44.9406738
3326	4006.150369	44.9322967
3327	4007.355413	44.923069
3328	4008.560004	44.914833
3329	4009.764468	44.9067878
3330	4010.968396	44.8986434
3331	4012.173249	44.8898429
3332	4013.377697	44.880516
3333	4014.582592	44.8717613
3334	4015.787381	44.8631858
3335	4016.991677	44.8552169
3336	4018.19646	44.8470573
3337	4019.399877	44.8400955
3338	4020.604517	44.8314361
3339	4021.808643	44.8237533
3340	4023.013598	44.8154335
3341	4024.217054	44.8078575
3342	4025.421946	44.801197
3343	4026.626835	44.7937889
3344	4027.831292	44.785614
3345	4029.035907	44.777584
3346	4030.240334	44.7692413
3347	4031.445425	44.7607955
3348	4032.648991	44.7533798
3349	4033.854268	44.744812

3350	4035.058949	44.7367134
3351	4036.263375	44.7281036
3352	4037.467294	44.7194404
3353	4038.671203	44.7110137
3354	4039.875058	44.703598
3355	4041.079427	44.6965637
3356	4042.284418	44.6888923
3357	4043.489786	44.6818847
3358	4044.693227	44.6736335
3359	4045.897924	44.6666259
3360	4047.10331	44.6594276
3361	4048.307942	44.6516914
3362	4049.512558	44.6436271
3363	4050.717783	44.6361694
3364	4051.921756	44.6276321
3365	4053.126326	44.6186141
3366	4054.331155	44.6099929
3367	4055.536157	44.6007614
3368	4056.740795	44.5931739
3369	4057.946102	44.5847969
3370	4059.149734	44.5753822
3371	4060.354786	44.5672302
3372	4061.559023	44.5589523
3373	4062.763969	44.5500411
3374	4063.969044	44.5418472
3375	4065.174346	44.5331535
3376	4066.377924	44.5252799
3377	4067.582874	44.5167083
3378	4068.788199	44.5073471
3379	4069.992482	44.4977149
3380	4071.196901	44.4893913
3381	4072.401253	44.4805335
3382	4073.604258	44.471302
3383	4074.808053	44.4623947
